# Synthesis of protected precursors of chitin oligosaccharides by electrochemical polyglycosylation of thioglycosides

**DOI:** 10.3762/bjoc.18.117

**Published:** 2022-08-30

**Authors:** Md Azadur Rahman, Kana Kuroda, Hirofumi Endo, Norihiko Sasaki, Tomoaki Hamada, Hiraku Sakai, Toshiki Nokami

**Affiliations:** 1 Department of Chemistry and Biotechnology, Tottori University, 4-101 Koyamacho-minami, Tottori City, 680-8552 Tottori, Japan; 2 Center for Research on Green Sustainable Chemistry, Faculty of Engineering, Tottori University, 4-101 Koyamacho-minami, Tottori City, 680-8552 Tottori, Japan; 3 Koganei Corporation, 3-11-28 Midorimachi, Koganei City, 184-8533 Tokyo, Japan

**Keywords:** electrochemical glycosylation, glucosamine, oligosaccharide, oxidation potential, polyglycosylation

## Abstract

The synthesis of protected precursors of chitin oligosaccharides by electrochemical polyglycosylation of thioglycosides as monomer is described. Oligosaccharides up to the hexasaccharide were synthesized under optimized reaction conditions. Further, a modified method enabled the synthesis of oligosaccharides up to the octasaccharide by repeating electrolysis with additional monomers. The mechanism of the electrochemical polyglycosylation is also discussed, based on the oxidation potential of the monomer and oligosaccharides.

## Introduction

**Figure 1:**
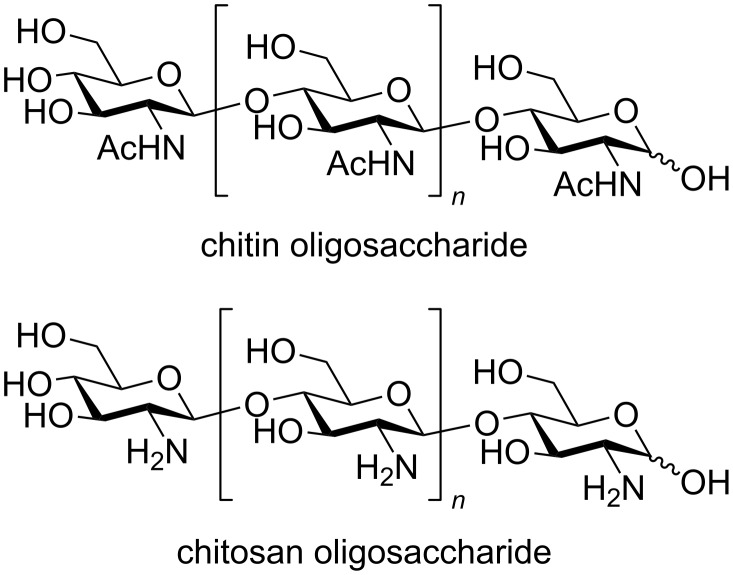
Structures of chitin and chitosan oligosaccharides.

Chitin oligosaccharides are partial structures of chitin, which is an abundant β-1,4-linked polysaccharide composed of *N*-acetylglucosamine as repeating unit (Figure 1) [1]. Biological activities of longer oligosaccharides, such as octasaccharide, have been paid much attention for many years. However, it is difficult to obtain pure oligosaccharides by isolation from natural sources or by synthesis via chemical glycosylation [2]. Total syntheses of chitin and chitosan oligosaccharides based on conventional chemical glycosylation of protected monosaccharides as building blocks have already been reported. Convergent synthesis using oligosaccharide building blocks can reduce the number of steps in the total synthesis. However, it requires manipulation of the anomeric leaving groups and deprotection of the protected hydroxy group at the 4-position prior to glycosylation. Although automated electrochemical assembly, which is a one-pot iterative synthesis of oligosaccharides based on electrochemical preactivation of building blocks, is an alternative method for the synthesis of chitin oligosaccharides [3–4], it is also time-consuming and too sophisticated to prepare oligosaccharides composed of a single repeating structure. Thus, we assume that the electrochemical polyglycosylation via the electrochemical activation of thioglycosides is a practical approach for the preparation of chitin oligosaccharides. Hashimoto and co-workers have already reported the synthesis of protected precursors of chitin oligosaccharides by polyglycosylation of thioglycosides [5]. However, this is one of a few examples of chemical synthesis of chitin oligosaccharides through polyglycosylation of a glucosamine monosaccharide [6]. Recently, we have reported electrochemical polyglycosylation using a glucosamine derivative as a monomer [7]. This is another example of polyglycosylation of a glucosamine monosaccharide. However, *N*-acetylglucosamines are linked by α-1,4-glycosidic bonds. Here, we report electrochemical polyglycosylation of thioglycosides to produce protected precursors of chitin oligosaccharides.

## Results and Discussion

### Optimization of electrochemical polyglycosylation

**Figure 2:**
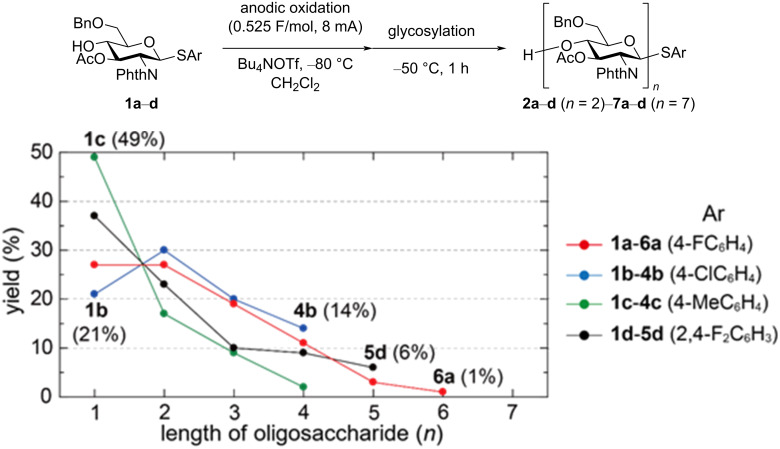
Effect of the anomeric leaving group on the yield of oligosaccharides.

We initiated our study with the optimization of the arylthio group of thioglycoside **1**, carrying an unprotected 4-OH group, an acetyl-protected 3-OH unit, a benzyl-protected 6-OH group, and a phthaloyl-protected 2-NH_2_ unit (Figure 2) [3]. Electrochemical polyglycosylation was performed by a sequential two-step process, which involved anodic oxidation at −80 °C and glycosylation at −50 °C. The crude product of the reaction was purified by gel permeation chromatography (GPC), and the monosaccharides **1a**–**d** and oligosaccharides **2a**–**d** (*n* = 2)–**7a**–**d** (*n* = 7) were isolated. Only thioglycoside **1a** (Ar = 4-FC_6_H_4_, *E*_ox_ = 1.70 V vs SCE) gave oligosaccharides up to hexasaccharide **6a**, although the yield of pentasaccharide **5a** (3%) and hexasaccharide **6a** (1%) was very low. For thioglycoside **1b** (Ar = 4-ClC_6_H_4_, *E*_ox_ = 1.68 V vs SCE), the highest conversion (79%) and the highest yield of tetrasaccharide **4b** (14%) were observed. Contrary, thioglycoside **1c** (Ar = 4-MeC_6_H_4_, *E*_ox_ = 1.47 V vs SCE), which had the lowest oxidation potential, showed the lowest conversion (51%) and the lowest yield of tetrasaccharide **4c** (2%) [8]. This being the case, lower conversion of the building block **1c** and lower yield of oligosaccharides **2c**–**4c** indicated that thus-generated oligosaccharides **2c**–**4c**, with a lower oxidation potential, also consumed electricity and converted to the corresponding hydroxy-substituted sugars, as observed by MS analysis. Thioglycoside **1d** (Ar = 2,4-F_2_C_6_H_3_, *E*_ox_ = 1.73 V vs SCE), which had the highest oxidation potential, also showed low conversion (63%). However, it gave pentasaccharide **5d** in the highest yield (6%) among these four thioglycosides. Based on these results, we optimized the reaction using thioglycoside **1a**, which afforded oligosaccharides **2a**–**6a** and recovered monosaccharide **1a** in the highest total yield (88%).

Reaction parameters of the electrochemical polyglycosylation, such as amount of electricity and electrolyte, were also optimized using thioglycoside **1a** (see Supporting Information File 1 for details). The complete conversion of monosaccharide **1a** was observed with 0.6 F/mol. However, 0.525 F/mol was chosen as the optimal amount of electricity to prevent formation of byproducts, such as hydroxy-substituted sugars that carry an anomeric hydroxy group instead of the ArS group. Although we tested other ammonium triflates, such as tetraethylammonium triflate and 1-butyl-1-methylpyrrolidinium triflate as electrolytes, both electrolytes gave oligosaccharides in lower yield.

**Figure 3:**
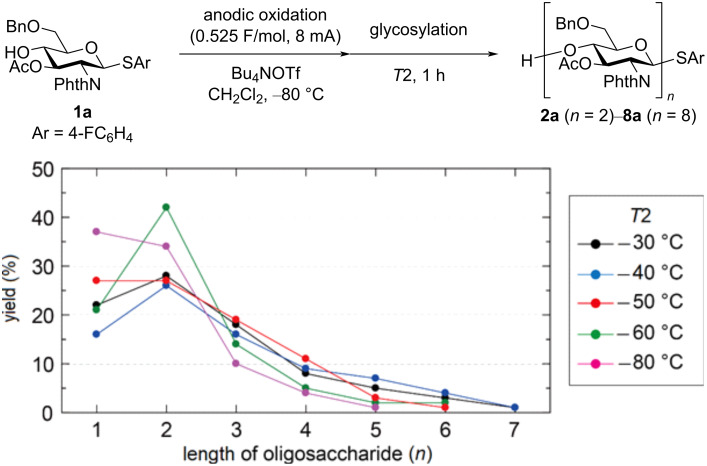
Influence of the glycosylation temperature (*T*2) on the yield of oligosaccharides.

Next, we investigated the influence of the glycosylation temperature (*T*2), and it was revealed that glycosylation proceeded even at −80 °C (Figure 3, in pink). Although higher conversion of thioglycoside **1a** was observed at higher temperature, we did not test a glycosylation temperature above −30 °C because of the low stability of the glycosylation intermediate at an elevated temperature [9]. It is important to note that heptasaccharide **7a**, which was never obtained at −50 °C, was produced at −40 °C (in blue) and −30 °C (in black), although the yield of **7a** was very low (1%). These results indicated that the glycosylation temperature was an important parameter for obtaining longer oligosaccharides, and glycosylation might proceed during the anodic oxidation at −80 °C.

**Figure 4:**
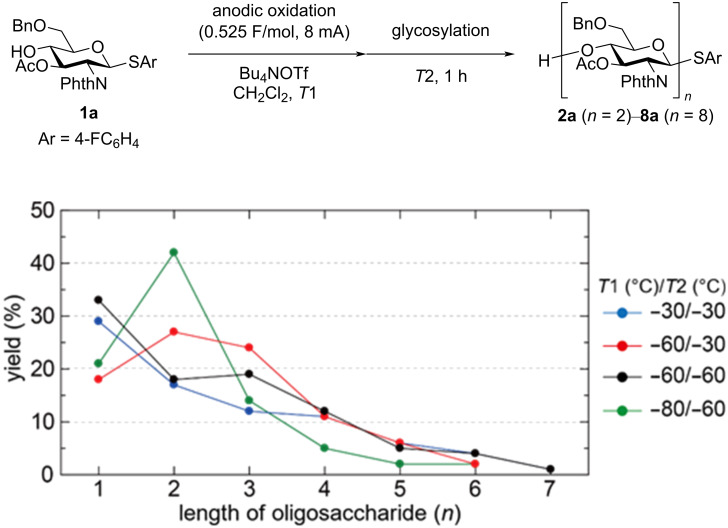
Influence of temperatures of anodic oxidation (*T*1) and glycosylation (*T*2).

**Figure 5:**
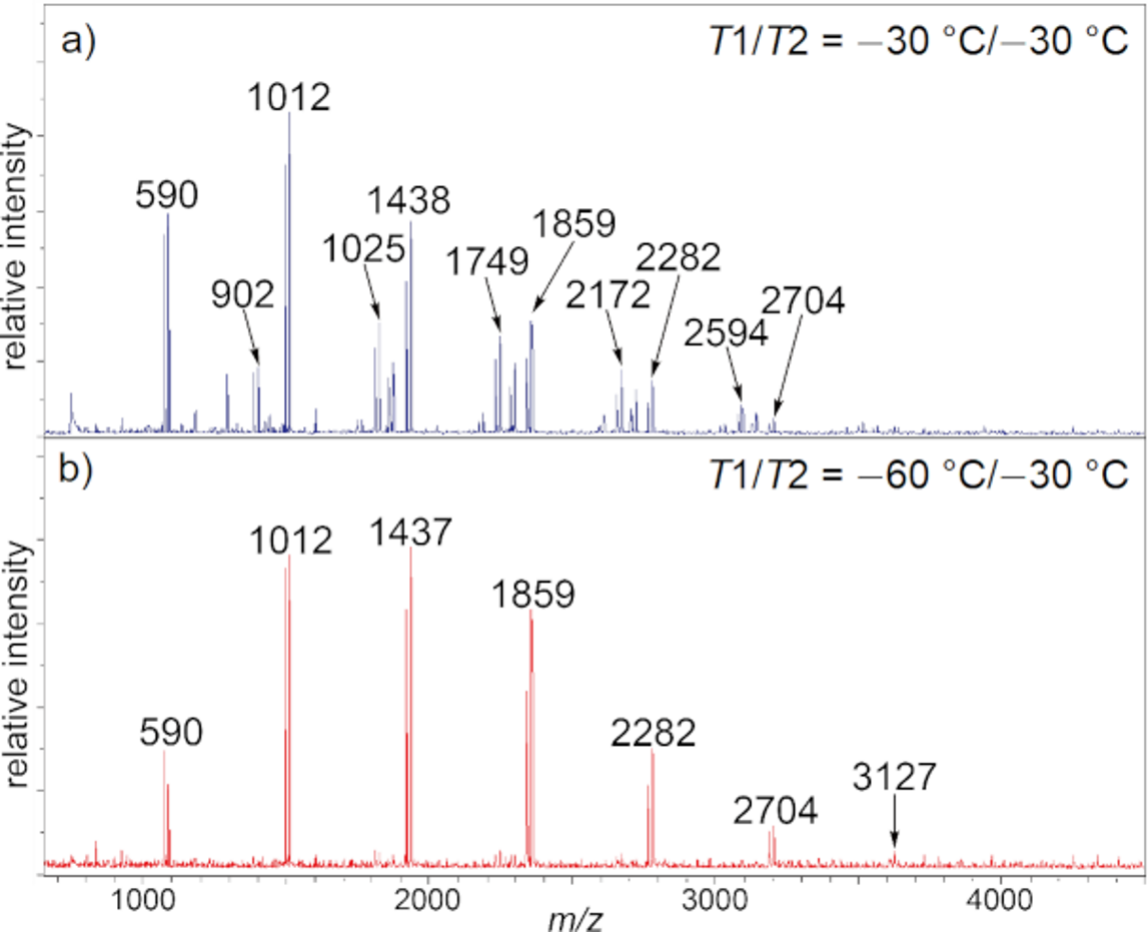
MALDI–TOF MS spectra of oligosaccharides.

**Figure 6:**
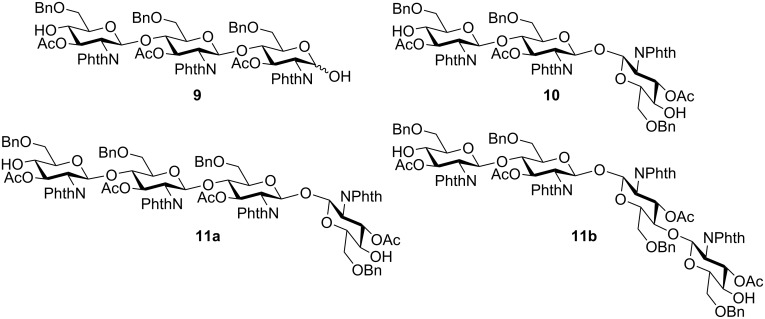
Proposed structures of byproducts of electrochemical polyglycosylation.

The temperature of anodic oxidation (*T*1) was also investigated together with the glycosylation temperature (*T*2) because glycosylation must occur during the anodic oxidation at elevated temperature (Figure 4). Indeed, formation of oligosaccharides longer than tetrasaccharide **4a** was increased at elevated temperature. The highest total yield of oligosaccharides **2a**–**7a** was obtained at *T*1 = −60 °C and *T*2 = −30 °C, although heptasaccharide **7a** was not produced. MALDI–TOF MS spectra indicated the formation of byproducts derived from longer oligosaccharides at *T*1 = −30 °C and *T*2 = −30 °C (Figure 5). The relative intensity of the molecular ion peaks of hydroxy-substituted sugars of oligosaccharides and/or trehalose pseudo-oligosaccharides, which were major byproducts at the elevated temperature, became stronger in the corresponding peaks of longer oligosaccharides, such as hexasaccharide **6a** and heptasaccharide **7a**. Proposed structures of byproducts of trisaccharide **3a**, including hydroxy-substituted sugar **9** and trehalose product **10**, are shown in Figure 6. These byproducts were obtained as inseparable mixture because of the same molecular weight and similar polarity. Moreover, the trehalose product of longer oligosaccharides has more than two possible structures. For example, there are two pseudo-tetrasaccharide structures **11a** and **11b**, which would be hard to separate by preparative-scale purification techniques.

### Reaction mechanism

**Figure 7:**
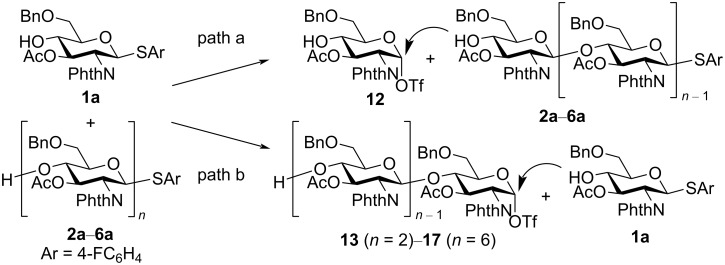
Proposed mechanisms of electrochemical polyglycosylation.

There are two possible pathways for chain elongation in electrochemical polyglycosylation (Figure 7). In path a, monosaccharide **1a** is converted to the corresponding glycosyl triflate **12**, and 4-OH of oligosaccharides **2a**–**6a** reacts with **12** [9]. In path b, oligosaccharides **2a**–**6a** are converted to the corresponding glycosyl triflates **13**–**17**, and 4-OH of monosaccharide **1a** reacts with **13**–**17**. It is difficult to exclude the possibility of reactions between oligosaccharides. However, polyglycosylation has been carried out with a slightly excessive amount of electricity (0.525 F/mol), and monosaccharide **1a** has always been recovered in more than 15% [10]. Moreover, longer oligosaccharides might be less reactive on grounds of mass transfer because electrochemical activation occurs at the surface of the anode and the substrates must move to the surface of the electrode.

**Figure 8:**
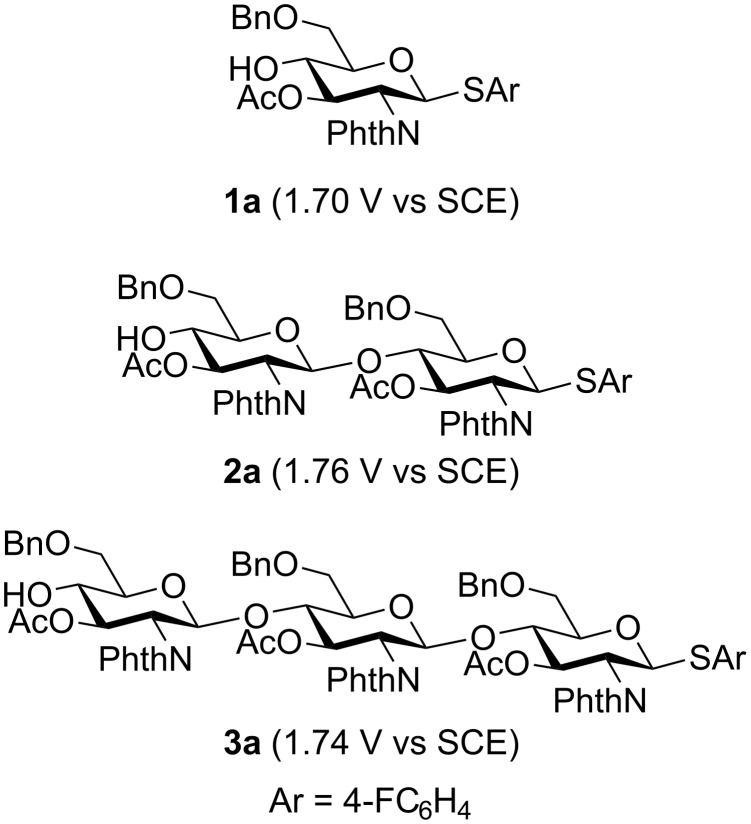
Oxidative potential of monosaccharide **1a**, disaccharide **2a**, and trisaccharide **3a**.

**Scheme 1:**
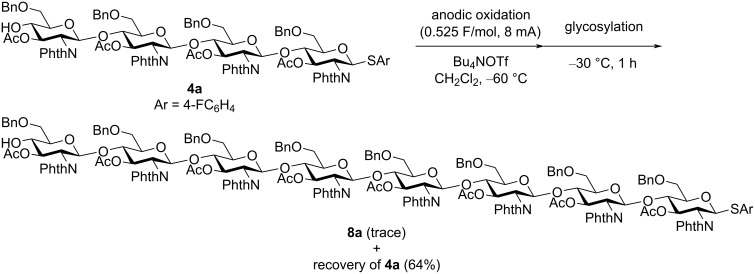
Electrochemical dimerization of tetrasaccharide **4a**.

To confirm the reactivity of oligosaccharides, we measured oxidation potentials of monosaccharide **1a**, disaccharide **2a**, and trisaccharide **3a** using a rotating disk electrode (RDE) made of glassy carbon (Figure 8). The oxidation potential of oligosaccharides **2a** and **3a** (*E*_ox_ = 1.76 and 1.74 V vs SCE) was higher than that of monosaccharide **1a** (*E*_ox_ = 1.70 V vs SCE). We also examined electrochemical activation of tetrasaccharide **4a** to obtain octasaccharide **8a** through the dimerization of **4a**. However, a trace amount of **8a** was formed together with byproducts, and the recovered amount of tetrasaccharide **4a** was 64% (Scheme 1). These results strongly suggested that path a in Figure 7 is the most probable mechanism of the reaction.

### Modification of electrochemical polyglycosylation protocol

**Figure 9:**
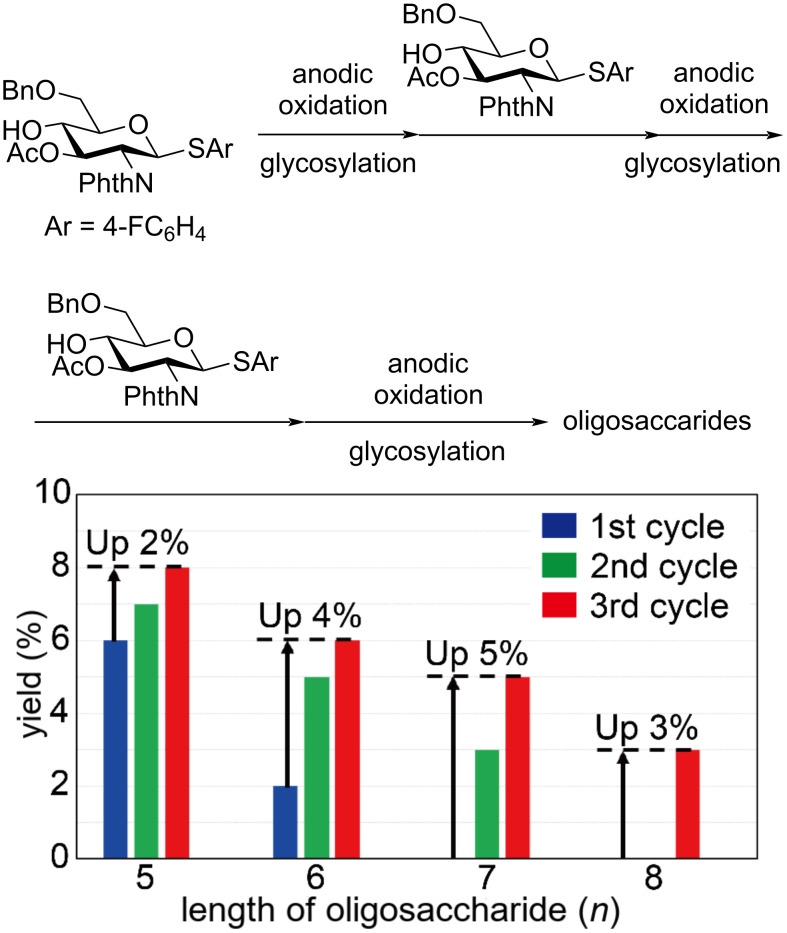
Influence of cycle number on the yield of longer oligosaccharides **5a** (*n* = 5)–**8a** (*n* = 8). Conditions of anodic oxidation: constant current (8.0 mA, 0.52 F/mol), temperature −60 °C for anodic oxidation and −30 °C for glycosylation, building block **1a** (0.20 mmol, 109 mg) per cycle as 0.2 M solution in dry CH_2_Cl_2_.

The optimized conditions of the electrochemical polyglycosylation can afford oligosaccharides up to the hexasaccharide **6a**. However, we were also interested in longer oligosaccharides, such as the heptasaccharide **7a** and the octasaccharide **8a** because of the biological activities [11]. Accordingly, the higher reactivity of monosaccharide **1a** compared to the oligosaccharides encouraged us to modify the electrochemical polyglycosylation protocol (Figure 9). We developed a modified electrochemical polyglycosylation method by repeating the addition of monosaccharide **1a** and anodic oxidation as a single cycle. To prove our concept, we ran the electrochemical polyglycosylation under the optimized conditions, and one equivalent of monosaccharide **1a** was added before the second anodic oxidation. After the second cycle, we could isolate heptasaccharide **7a** (3%, 1.5 μmol, 4.7 mg) together with an increased amount of hexasaccharide **6a** (5%, 3.1 μmol, 8.2 mg). We ran the process up to the third cycle and isolated octasaccharide **8a** (3%, 2.3 μmol, 7.6 mg), which was never isolated after the first cycle and the second cycle. These results also supported the proposed reaction mechanism path a in Figure 7.

## Conclusion

In conclusion, we have developed a practical method to synthesize longer-chain oligosaccharides within a short period of time through electrochemical polyglycosylation. A rational reaction mechanism was proposed based on oxidation potentials of the oligosaccharides, and further modification of the protocol was examined. By repeating cycles in one pot, the modified method enabled us to prepare longer oligosaccharides up to the octasaccharide. Further optimizations of reaction parameters, such as concentration, size and shape of electrodes for large-scale production of oligosaccharides, and deprotection of oligosaccharides thus obtained are in progress in our laboratory.

## Experimental

Electrochemical polyglycosylation (see Figure 4, *T*1 = −60 °C and *T*2 = −30 °C) was performed using our second-generation automated electrochemical synthesizer equipped with the H-type electrolysis cell. Thioglycoside **1a** (0.20 mmol, 109 mg), Bu_4_NOTf (1.0 mmol, 393 mg), and dry CH_2_Cl_2_ (10 mL) were added to the anodic chamber. Triflic acid (0.2 mmol, 17.6 μL) and CH_2_Cl_2_ (10 mL) were added to the cathodic chamber. Electrolysis was performed at −60 °C under constant current conditions until 0.52 F/mol of electricity had been consumed. Then, the reaction temperature was elevated to −30 °C, and the temperature was kept for 1 h. The reaction was quenched with Et_3_N (0.30 mL), and the reaction mixture was diluted with Et_2_O and EtOAc and washed with water to remove the electrolyte. The combined organic layer was dried with Na_2_SO_4_, and the solvent was removed under reduced pressure. The thus-obtained crude product (110 mg) was purified by preparative GPC using CHCl_3_ as eluent.

## Supporting Information

File 1 Additional experimental details and compound characterization data.
