# Supplementary material for: Synthesis of protected precursors of chitin oligosaccharides by electrochemical polyglycosylation of thioglycosides
Source: Beilstein J Org Chem. 2022 Aug 30;18:1133–9. doi: 10.3762/bjoc.18.117 (PMC9443410; doi:10.3762/bjoc.18.117)
Supplement: File 1 — Additional experimental details and compound characterization data. [file Beilstein_J_Org_Chem-18-1133-s001.pdf]

BEILSTEIN JOURNAL OF ORGANIC CHEMISTRY

Supporting Information

for

Synthesis of protected precursors of chitin oligosaccharides by electrochemical polyglycosylation of thioglycosides

Md Azadur Rahman, Kana Kuroda, Hirofumi Endo, Norihiko Sasaki, Tomoaki Hamada, Hiraku Sakai and Toshiki Nokami

Beilstein J. Org. Chem. **2022,** *18,* 1133–1139. doi:10.3762/bjoc.18.117

Additional experimental details and compound characterization data

License and Terms: This is a supporting information file under the terms of the Creative Commons Attribution License (https://creativecommons.org/licenses/by/4.0). Please note that the reuse, redistribution and reproduction in particular requires that the author(s) and source are credited and that individual graphics may be subject to special legal provisions.
The license is subject to the *Beilstein Journal of Organic Chemistry* terms and conditions: (https://www.beilstein-journals.org/bjoc/terms)

Contents

1. General S2
2. Synthesis of oligosaccharides by electrochemical polyglycosylation S2
3. Optimization of electricity and electrolyte S13
4. Influence of reaction parameters S14
5. Measurement of oxidation potential of oligosaccharides S16
6. Electrochemical dimerization of tetrasaccharide S17
7. Protocol modification of electrochemical polyglycosylation S18
8. References S19
9. ^{1}H and ^{13}C NMR, H,H-COSY, and HMQC spectra of oligosaccharides S20

1. General

All reactions were carried out under argon atmosphere except where otherwise noted. ^{1}H NMR and ^{13}C NMR spectra were recorded on Bruker AVANCE II 600 (600 MHz for ^{1}H and 150 MHz for ^{13}C) and JEOL JNM-ECZ600 spectrometers (600 MHz for ^{1}H and 150 MHz for ^{13}C). ESI-MS spectra were recorded on a Thermo Scientific Exactive spectrometer. MALDI-TOF MS spectra were recorded on a Bruker Ultraflextreme spectrometer. Optical rotation data was recorded on a JASCO DIP-370 digital polarimeter. Merck TLC plates (silica gel 60 F_{254}) were employed for TLC analysis. Gel permeation chromatography (GPC) was used with JAI Labo Ace LC-5060 recycling preparative HPLC (eluent: $CHCl_3$). Kanto silica gel (spherical, neutral, 63–210 μm) and Sephadex LH-20 were used for silica gel chromatography and gel filtration chromatography, respectively. Rotating disk electrode voltammetry was carried out using a BAS 700c analyzer and a RRDE-3 rotating ring disk electrode. Measurements of oxidation potential of substrates (c = 4.0 mM) were carried out in 0.1 M Bu_4NOTf/CH_2Cl_2 using a glassy carbon disk working electrode, a platinum wire counter electrode, and a saturated calomel electrode (SCE) as a reference electrode, with a sweep rate of 10 mV/s at 2000 rpm. Compounds **1a** [1], **1b** [2], **1c** [1], and **1d** [1] were synthesized according to the reported procedures. Unless otherwise mentioned, all reagents were obtained from commercial suppliers and used without extra purification.

2. Synthesis of oligosaccharides by electrochemical polyglycosylation

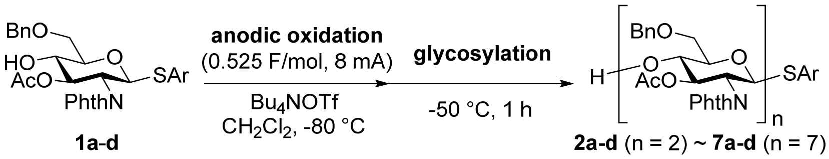

The electrochemical polymerization synthesis of linear oligosaccharides (**2a**–**7a**) was carried out in an H-type divided cell (4G glass filter). The cell had a carbon felt anode (Nippon Carbon JF-20-P7) and platinum square plate (20 mm × 20 mm). Building block **1a** (0.39 mmol, 218 mg), Bu_4NOTf (1.00 mmol, 393 mg), and CH_2Cl_2 (20 mL) were added to the anodic chamber. Trifluoromethanesulfonic acid (0.4 mmol, 35 μL), Bu_4NOTf (1.00 mmol, 393 mg), and CH_2Cl_2 (20 mL) were added to the cathodic chamber. The constant current (8 mA (current density: 2.0 mA/cm^2), 45 V (electrode distance: 4.5 cm)) was employed at −80 °C with magnetic stirring until 0.52 F/mol of the electricity was consumed. After the electrolysis, the reaction was kept stirring at −50 °C for 1 h. After that, triethylamine (0.3 mL) was added to both chambers. The solution in both chambers was collected in an "eggplant" flask, and the solvent was removed under reduced pressure. The mixture was dissolved in EtOAc and washed with water (3 ×) and brine, respectively. The solution was dried over Na_2SO_4, and the solvent was removed under reduced pressure. The crude product was purified with preparative GPC to afford linear oligosaccharides **2a** (n = 2, 53 μmol, 52.0 mg, 27%), **3a** (n = 3, 25 μmol, 34.7

mg, 19%), **4a** (*n* = 4, 11 μmol, 19.3 mg, 11%), **5a** (*n* = 5, 2.2 μmol, 5.0 mg, 3%), **6a** (*n* = 6, 0.90 μmol, 2.4 mg, 1%), and **7a** (*n* = 7, trace) as white solids. Recovered yield of buliding block **1a** was 27% (58.2 mg, 106 μmol).

4-Fluorophenyl (3-*O*-acetyl-6-*O*-benzyl-2-deoxy-2-phthalimido-β-D-glucopyranosyl)-(1→4)-3-*O*-acetyl-6-*O*-benzyl-2-deoxy-2-phthalimido-1-thio-β-D-glucopyranoside (**2a**); TLC (Hexane:EtOAc 1:2): R_f 0.57. $[\alpha]_D$ = –7.88 (*c* = 1.0, $CHCl_3$, 26 °C). E_{ox} = 1.76 V vs. SCE; ^{1}H NMR ($CDCl_3$, 600 MHz) δ 7.86–7.77 (m, 4 H), 7.76–7.72 (m, 2 H), 7.71–7.67 (m, 2 H), 7.35–7.32 (m, 6 H), 7.31–7.26 (m, 4 H), 7.22 (d, *J* = 7.0 Hz, 2 H), 6.82 (*pseudo*-t, *J* = 8.6 Hz, 2 H), 5.67 (dd, *J* = 9.9, 8.9 Hz, 1 H), 5.57 (dd, *J* = 10.6, 8.9 Hz, 1 H), 5.50 (d, *J* = 10.5 Hz, 1 H), 5.45 (d, *J* = 8.3 Hz, 1 H), 4.54 (d, *J* = 11.8 Hz, 1 H), 4.49 (d, *J* = 11.8 Hz, 1 H), 4.37 (d, *J* = 11.8 Hz, 1 H), 4.31 (d, *J* = 11.9 Hz, 1 H), 4.15 (*pseudo*-t, *J* = 10.3 Hz, 1 H), 4.11 (dd, *J* = 10.7, 8.3 Hz, 1 H), 4.03 (*pseudo*-t, *J* = 9.2 Hz, 1 H), 3.81 (td, *J* = 9.2, 3.2 Hz, 1 H), 3.75 (dd, *J* = 10.0, 4.0 Hz, 1 H), 3.66 (dd, *J* = 10.0, 4.9 Hz, 1 H), 3.52 (dd, *J* = 9.8, 2.3 Hz, 2 H), 3.49–3.43 (m, 2 H), 2.96 (d, *J* = 2.8 Hz, 1 H), 1.88 (s, 3 H), 1.82 (s, 3 H).; ^{13}C NMR ($CDCl_3$, 150 MHz) δ 171.0, 170.0, 167.8, 167.3, 163.0 (d, *J* = 247.5 Hz), 138.2, 137.4, 136.1 (d, *J* = 9.0 Hz), 134.4, 134.3, 143.2, 131.7,131.4, 131.2, 128.5, 128.3, 128.0, 127.7, 127.5, 127.4, 125.8 (d, *J* = 3.0 Hz), 123.7, 123.5, 115.9 (d, *J* = 22.5 Hz), 97.2, 82.6, 78.5, 74.1, 73.6, 73.4, 73.2, 72.7, 72.4, 71.4, 70.0, 67.8, 54.9, 53.8, 20.63, 20.61; HRMS (ESI) *m/z* calculated for $C_{52}H_{47}FKN_2O_{14}S$ $[M+K]^+$, 1013.2364; found, 1013.2322.

4-Fluorophenyl (3-*O*-acetyl-6-*O*-benzyl-2-deoxy-2-phthalimido-β-D-glucopyranosyl)-(1→4)-(3-*O*-acetyl-6-*O*-benzyl-2-deoxy-2-phthalimido-β-D-glucopyranosyl)-(1→4)-3-*O*-acetyl-6-*O*-benzyl-2-deoxy-2-phthalimido-1-thio-β-D-glucopyranoside (**3a**); TLC (Hexane:EtOAc 1:2): R_f 0.50. $[\alpha]_D$ = –15.8 (*c* = 1.0, $CHCl_3$, 26 °C). E_{ox} = 1.74 V vs. SCE; ^{1}H NMR ($CDCl_3$, 600 MHz) δ 7.88–7.77 (m, 6 H), 7.76–7.67 (m, 6 H), 7.35–7.31 (m, 4 H), 7.30–7.26 (m, 5 H), 7.25–7.20 (m, 5 H), 7.14 (*pseudo*-t, *J* = 7.8 Hz, 2 H), 6.82 (*pseudo*-t, *J* = 8.6 Hz, 2 H), 5.58 (*pseudo*-t, *J* = 9.4 Hz, 1 H), 5.54 (td, *J* = 10.6, 1.6 Hz, 1 H), 5.51 (td, *J* = 10.6, 1.6 Hz, 1 H), 5.46 (d, *J* = 10.5 Hz, 1 H), 5.38 (d, *J* = 8.3 Hz, 1 H), 5.27 (d, *J* = 8.4 Hz, 1 H), 4.52 (d, *J* = 11.7 Hz, 1 H), 4.47 (d, *J* = 11.8 Hz, 1 H), 4.43 (d, *J* = 11.8 Hz, 1 H), 4.42 (d, *J* = 11.6 Hz, 1 H), 4.38 (d, *J* = 11.8 Hz, 1 H), 4.31 (d, *J* = 11.6 Hz, 1 H), 4.14 (dd, *J* = 9.4, 5.5 Hz, 1 H), 4.12 (dd, *J* = 9.4, 4.4 Hz, 1 H), 4.07 (dd, *J* = 10.7, 8.3 Hz, 1 H), 4.02 (dd, *J* = 10.4, 8.2 Hz, 1 H), 3.99 (*pseudo*-t, *J* = 9.4 Hz, 1 H), 3.79 (td, *J* = 9.2, 3.2 Hz, 1 H), 3.72 (dd, *J* = 9.9, 4.0 Hz, 1 H), 3.63 (dd, *J* = 9.9, 4.9 Hz, 1 H), 3.54 (d, *J* = 10.4 Hz, 1 H), 3.46 (dd, *J* = 10.7, 3.7 Hz, 1 H), 3.42 (d, *J* = 10.9 Hz, 2 H), 3.30 (dd, *J* = 11.2, 3.5 Hz, 1 H), 3.27 (dd, *J* = 9.2, 4.4 Hz, 1 H), 3.10 (d, *J* = 8.8 Hz, 1 H), 2.88 (d, *J* = 3.3 Hz, 1 H), 1.80 (s, 3 H), 1.71 (s, 3 H), 1.63 (s, 3 H); ^{13}C NMR ($CDCl_3$, 150 MHz) δ 171.0, 170.2, 170.1, 168.1, 167.8, 167.2 163.0 (d, *J* = 247.5 Hz), 138.2, 138.1, 137.4, 136.0 (d, *J* = 9.0 Hz), 134.4, 134.3, 134.1, 131.7, 131.2, 128.5, 128.2, 128.1, 127.9, 127.6,

127.4, 127.36, 127.26, 127.1 125.9 (d, *J* = 3.3 Hz), 123.6, 123.5, 115.9 (d, *J* = 22.5 Hz), 96.6, 96.5, 82.6, 78.5, 74.0, 73.6, 72.6, 72.3, 71.7, 71.4, 71.2, 70.0, 67.9, 67.3 55.3, 54.9, 53.8, 20.61, 20.57, 20.46; HRMS (ESI) *m/z* calculated for $C_{75}H_{68}FKN_3O_{21}S$ $[M+K]^+$, 1436.3682; found, 1436.3613.

4-Fluorophenyl (3-*O*-acetyl-6-*O*-benzyl-2-deoxy-2-phthalimido-β-D-glucopyranosyl)-(1→4)-(3-*O*-acetyl-6-*O*-benzyl-2-deoxy-2-phthalimido-β-D-glucopyranosyl)-(1→4)-(3-*O*-acetyl-6-*O*-benzyl-2-deoxy-2-phthalimido-β-D-glucopyranosyl)-(1→4)-3-*O*-acetyl-6-*O*-benzyl-2-deoxy-2-phthalimido-1-thio-β-D-glucopyranoside (**4a**); TLC (Hexane:EtOAc 1:2): R_f 0.37. $[\alpha]_D$ = –22.9 (*c* = 1.1, $CHCl_3$, 24 °C). ^{1}H NMR ($CDCl_3$, 600 MHz) δ 7.89–7.65 (m, 16 H), 7.35–7.26 (m, 9 H), 7.25–7.17 (m, 7 H), 7.10 (*pseudo*-t, *J* = 7.8 Hz, 2 H), 6.99 (*pseudo*-t, *J* = 7.8 Hz, 2 H), 6.94 (*pseudo*-t, *J* = 7.2 Hz, 1 H), 6.82 (*pseudo*-t, *J* = 8.4 Hz, 2 H), 6.69 (*pseudo*-t, *J* = 7.2 Hz, 1 H), 5.57 (dd, *J* = 10.2, 9.0 Hz, 1 H), 5.49 (dd, *J* = 10.8, 9.0 Hz, 1 H), 5.48–5.44 (m, 3 H), 5.34 (d, *J* = 8.4 Hz, 1 H), 5.21 (d, *J* = 8.4 Hz, 1 H), 5.18 (d, *J* = 8.4 Hz, 1 H), 4.52 (d, *J* = 11.4 Hz, 1 H), 4.47 (d, *J* = 11.4 Hz, 1 H), 4.45–4.33 (m, 5 H), 4.32 (d, *J* = 11.4 Hz, 1 H), 4.14 (d, *J* = 10.8 Hz, 1 H), 4.10 (d, *J* = 9.0 Hz, 1 H), 4.09–3.95 (m, 5 H), 3.77 (*pseudo*-t, *J* = 9.6 Hz, 1 H), 3.71 (dd, *J* = 10.2, 4.2 Hz, 1 H), 3.62 (dd, *J* = 9.6, 4.8 Hz, 1 H), 3.53 (d, *J* = 9.6 Hz, 1 H), 3.47–3.43 (m, 2 H), 3.41–3.37 (m, 2 H), 3.26–3.20 (m, 3 H), 3.01 (dd, *J* = 9.6, 1.8 Hz, 1 H), 2.86 (s, 1 H), 2.79 (d, *J* = 9.0 Hz, 1 H), 1.87 (s, 3 H), 1.79 (s, 3 H), 1.73 (s, 3 H), 1.67 (s, 3 H); ^{13}C NMR ($CDCl_3$, 150 MHz) δ 171.0, 170.4, 170.3, 170.2, 168.2, 167.8, 167.3, 163.0 (d, *J* = 247.2 Hz), 138.3, 138.19, 138.17, 137.5, 136.0 (d, *J* = 8.7 Hz), 134.5, 134.4, 134.3, 134.2, 131.7, 131.5, 131.2, 128.6, 128.3, 128.11, 128.03, 127.97, 127.7, 127.5, 127.3, 127.2, 127.0, 126.0 (d, *J* = 3.3 Hz), 123.7, 123.63, 123.58, 123.45, 123.39, 115.9 (d, *J* = 20.9 Hz), 96.60, 96.56, 96.0, 82.7, 78.5, 73.9, 73.8, 73.6, 73.38, 73.33, 73.1, 73.0, 72.6, 72.33, 72.30, 72.1, 71.8, 71.3, 70.8, 69.9, 67.9, 67.5, 55.4, 55.2, 55.0, 53.8, 20.67, 20.65, 20.53; HRMS (ESI) *m/z* calculated for $C_{98}H_{89}FN_4NaO_{28}S$ $[M+Na]^+$, 1843.5260; found, 1843.5217.

4-Fluorophenyl (3-*O*-acetyl-6-*O*-benzyl-2-deoxy-2-phthalimido-β-D-glucopyranosyl)-(1→4)-(3-*O*-acetyl-6-*O*-benzyl-2-deoxy-2-phthalimido-β-D-glucopyranosyl)-(1→4)-(3-*O*-acetyl-6-*O*-benzyl-2-deoxy-2-phthalimido-β-D-glucopyranosyl)-(1→4)-(3-*O*-acetyl-6-*O*-benzyl-2-deoxy-2-phthalimido-β-D-glucopyranosyl)-(1→4)-3-*O*-acetyl-6-*O*-benzyl-2-deoxy-2-phthalimido-1-thio-β-D-glucopyranoside (**5a**); TLC (Hexane:EtOAc 1:2): R_f 0.28. $[\alpha]_D$ = –27.0 (*c* = 1.2, $CHCl_3$, 25 °C); ^{1}H NMR ($CDCl_3$, 600 MHz) δ 7.89–7.64 (m, 20 H), 7.34–7.25 (m, 10 H), 7.23–7.14 (m, 9 H), 7.07 (*pseudo*-t, *J* = 7.8 Hz, 2 H), 6.92 (*pseudo*-t, *J* = 7.8 Hz, 2 H), 6.90 (*pseudo*-t, *J* = 7.8 Hz, 2 H), 6.81 (*pseudo*-t, *J* = 9.0 Hz, 2 H), 6.61 (*pseudo*-t, *J* = 7.2 Hz, 1 H), 6.55 (*pseudo*-t, *J* = 7.2 Hz, 1 H), 5.55 (dd, *J* = 10.2, 9.6 Hz, 1 H), 5.50–5.40 (m, 4 H), 5.36 (dd, *J* = 10.2, 9.0 Hz, 1 H), 5.31 (d, *J* = 8.4 Hz, 1 H), 5.18 (d, *J* = 8.4 Hz, 1 H), 5.12 (d, *J* = 8.4 Hz, 1 H), 5.10 (d, *J* = 8.4 Hz, 1 H), 4.50 (d, *J* = 11.4 Hz, 1 H), 4.46 (d, *J* = 11.4 Hz, 1 H), 4.44–4.28 (m, 8 H), 4.13–3.90 (m, 10 H), 3.75 (td, *J* = 9.6, 3.6

Hz, 1 H), 3.69 (dd, *J* = 9.6, 3.6 Hz, 1 H), 3.61 (dd, *J* = 9.6, 4.8 Hz, 1 H), 3.51 (d, *J* = 9.6 Hz, 1 H), 3.46–3.33 (m, 4 H), 3.23–3.12 (m, 4 H), 2.97 (d, *J* = 9.0 Hz, 1 H), 2.89 (d, *J* = 3.6 Hz, 1 H), 2.71 (d, *J* = 9.0 Hz, 1 H), 2.65 (d, *J* = 8.4 Hz, 1 H), 1.85 (s, 3 H), 1.77 (s, 3 H), 1.72 (s, 3 H), 1.69 (s, 3 H), 1.62 (s, 3 H); ^{13}C NMR ($CDCl_3$, 150 MHz) δ 170.9, 170.33, 170.30, 170.2, 170.1, 168.0, 167.7, 167.20, 167.16, 162.9 (d, *J* = 247.2 Hz), 138.22, 138.13, 138.12, 138.08, 137.4, 135.9 (d, *J* = 7.7 Hz), 134.4, 134.29, 134.23, 134.16, 134.1, 131.6, 131.5, 131.3, 131.1, 128.5, 128.26, 128.20, 128.12, 128.0, 127.89, 127.87, 127.6, 127.42, 127.38, 127.36, 127.19, 127.15, 127.06, 126.90, 126.85, 125.9 (d, *J* = 3.3 Hz), 123.63, 123.54, 123.49, 123.39, 123.33, 115.8 (d, *J* = 21.9 Hz), 96.5, 96.4, 95.9, 95.8, 82.7, 73.8, 73.6, 73.3, 73.2, 73.0, 72.9, 72.5, 72.2, 72.0, 71.9, 71.7, 71.3, 71.2, 70.7, 70.6, 69.9, 67.8, 67.5, 67.4, 55.3, 55.2, 55.1, 54.8, 53.7, 20.58, 20.55, 20.44; HRMS (ESI) *m/z* calculated for $C_{121}H_{110}FN_5NaO_{35}S$ $[M+Na]^+$, 2266.6578; found, 2266.6513.

4-Fluorophenyl (3-*O*-acetyl-6-*O*-benzyl-2-deoxy-2-phthalimido-β-D-glucopyranosyl)-(1→4)-(3-*O*-acetyl-6-*O*-benzyl-2-deoxy-2-phthalimido-β-D-glucopyranosyl)-(1→4)-(3-*O*-acetyl-6-*O*-benzyl-2-deoxy-2-phthalimido-β-D-glucopyranosyl)-(1→4)-(3-*O*-acetyl-6-*O*-benzyl-2-deoxy-2-phthalimido-β-D-glucopyranosyl)-(1→4)-(3-*O*-acetyl-6-*O*-benzyl-2-deoxy-2-phthalimido-β-D-glucopyranosyl)-(1→4)-3-*O*-acetyl-6-*O*-benzyl-2-deoxy-2-phthalimido-1-thio-β-D- glucopyranoside (**6a**); TLC (Hexane:EtOAc 1:2): R_f 0.20. $[\alpha]_D$ = –28.9 (*c* = 0.9, $CHCl_3$, 28 °C); ^{1}H NMR ($CDCl_3$, 600 MHz) δ 7.90–7.64 (m, 24 H), 7.34–7.26 (m, 9 H), 7.23–7.15 (m, 12 H), 7.08 (*pseudo*-t, *J* = 7.8 Hz, 2 H), 6.94 (*pseudo*-t, *J* = 7.8 Hz, 2 H), 6.93–6.85 (m, 4 H), 6.82 (*pseudo*-t, *J* = 8.4 Hz, 2 H), 6.61 (*pseudo*-t, *J* = 7.2 Hz, 1 H), 6.53 (*pseudo*-t, *J* = 7.8 Hz, 1 H), 6.49 (*pseudo*-t, *J* = 7.2 Hz, 1 H), 5.56 (dd, *J* = 10.2, 9.0 Hz, 1 H), 5.48 (dd, *J* = 10.8, 9.0 Hz, 1 H), 5.46–5.41 (m, 3 H), 5.38–5.31 (m, 3 H), 5.18 (d, *J* = 8.4 Hz, 1 H), 5.12 (d, *J* = 8.4 Hz, 1 H), 5.10 (d, *J* = 8.4 Hz, 1 H), 5.06 (d, *J* = 8.4 Hz, 1 H), 4.51 (d, *J* = 11.4 Hz, 1 H), 4.46 (d, *J* = 11.4 Hz, 1 H), 4.44–4.29 (m, 10 H), 4.14–3.89 (m, 12 H), 3.76 (td, *J* = 9.6, 3.6 Hz, 1 H), 3.70 (dd, *J* = 9.6, 3.6 Hz, 1 H), 3.62 (dd, *J* = 9.6, 4.8 Hz, 1 H), 3.52 (d, *J* = 10.2 Hz, 1 H), 3.45–3.35 (m, 5 H), 3.23–3.16 (m, 3 H), 3.14–3.09 (m, 2 H), 2.98 (d, *J* = 9.0 Hz, 1 H), 2.89 (d, *J* = 3.6 Hz, 1 H), 2.70 (d, *J* = 9.6 Hz, 1 H), 2.64 (d, *J* = 9.0 Hz, 1 H), 2.59 (d, *J* = 9.6 Hz, 1 H), 1.86 (s, 3 H), 1.78 (s, 3 H), 1.73 (s, 3 H), 1.71 (s, 3 H), 1.69 (s, 3 H), 1.65 (s, 3 H); ^{13}C NMR ($CDCl_3$, 150 MHz) δ 171.2, 170.34, 170.30, 170.2, 170.1, 168.01, 167.97, 167.7, 167.2, 167.1 162.9 (d, *J* = 247.2 Hz), 138.2, 138.15, 138.11, 138.08, 137.3, 135.9 (d, *J* = 7.7 Hz), 134.3, 134.1, 131.60, 131.56, 131.4, 131.1, 128.5, 128.2, 128.0, 127.90, 127.85, 127.80, 127.6, 127.42, 127.35, 127.26, 127.18, 127.14, 127.05, 126.82, 126.79, 125.9 (d, *J* = 3.3 Hz), 123.66, 123.54, 123.49, 123.38, 115.8 (d, *J* = 21.9 Hz), 96.49, 96.41, 95.89, 95.74, 82.7, 78.4, 73.8, 73.6, 73.5, 73.3, 73.1, 73.0, 72.5, 72.2, 71.9, 71.7, 71.3, 71.2, 70.73, 70.70, 70.6, 69.9, 67.8, 67.5, 67.4, 55.27, 55.14, 55.11, 55.08, 54.8, 53.7, 20.58, 20.55, 20.44; HRMS (ESI) *m/z* calculated for $C_{144}H_{131}FN_6NaO_{42}S$ $[M+Na]^+$, 2689.7896; found, 2689.7849.

4-Fluorophenyl (3-*O*-acetyl-6-*O*-benzyl-2-deoxy-2-phthalimido-β-D-glucopyranosyl)-(1→4)-(3-*O*-acetyl-6-*O*-benzyl-2-deoxy-2-phthalimido-β-D-glucopyranosyl)-(1→4)-(3-*O*-acetyl-6-*O*-benzyl-2-deoxy-2-phthalimido-β-D-glucopyranosyl)-(1→4)-(3-*O*-acetyl-6-*O*-benzyl-2-deoxy-2-phthalimido-β-D-glucopyranosyl)-(1→4)-(3-*O*-acetyl-6-*O*-benzyl-2-deoxy-2-phthalimido-β-D-glucopyranosyl)-(1→4)-(3-*O*-acetyl-6-*O*-benzyl-2-deoxy-2-phthalimido-β-D-glucopyranosyl)-(1→4)-3-*O*-acetyl-6-*O*-benzyl-2-deoxy-2-phthalimido-1-thio-β-D-glucopyranoside (**7a**); TLC (Hexane:EtOAc 1:2): R_f 0.17. $[\alpha]_D$ = –28.9 (*c* = 0.64, $CHCl_3$, 28 °C); ^1H NMR ($CDCl_3$, 600 MHz) δ 7.89–7.66 (m, 28 H), 7.35–7.27 (m, 10 H), 7.24–7.13 (m, 13 H), 7.08 (*pseudo*-t, *J* = 7.8 Hz, 2 H), 6.94 (*pseudo*-t, *J* = 7.8 Hz, 2 H), 6.92–6.84 (m, 6 H), 6.82 (*pseudo*-t, *J* = 7.8 Hz, 2 H), 6.61 (*pseudo*-t, *J* = 7.8 Hz, 1 H), 6.52 (*pseudo*-t, *J* = 7.2 Hz, 1 H), 6.48 (*pseudo*-t, *J* = 7.8 Hz, 1 H), 6.46 (*pseudo*-t, *J* = 7.8 Hz, 1 H), 5.56 (dd, *J* = 10.2, 9.0 Hz, 1 H), 5.48 (dd, *J* = 10.8, 9.0 Hz, 1 H), 5.46–5.40 (m, 3 H), 5.37–5.30 (m, 4 H), 5.18 (d, *J* = 8.4 Hz, 1 H), 5.11 (d, *J* = 9.0 Hz, 1 H), 5.09 (d, *J* = 8.4 Hz, 1 H), 5.048 (d, *J* = 8.4 Hz, 1 H), 5.046 (d, *J* = 8.4 Hz, 1 H), 4.51 (d, *J* = 11.4 Hz, 1 H), 4.46 (d, *J* = 11.4 Hz, 1 H), 4.44–4.37 (m, 6 H), 4.36–4.28 (m, 6 H), 4.14–3.88 (m, 16 H), 3.79–3.74 (m, 1 H), 3.70 (dd, *J* = 9.6, 3.6 Hz, 1 H), 3.61 (dd, *J* = 10.2, 4.8 Hz, 1 H), 3.52 (d, *J* = 10.2 Hz, 1 H), 3.45–3.35 (m, 6 H), 3.23–3.16 (m, 3 H), 3.13–3.07 (m, 2 H), 2.97 (d, *J* = 9.6 Hz, 1 H), 2.87 (s, 1 H), 2.70 (d, *J* = 10.2 Hz, 1 H), 2.63 (d, *J* = 9.0 Hz, 1 H), 2.58–2.55 (m, 1 H), 1.86 (s, 3 H), 1.77 (s, 3 H), 1.73 (s, 3 H), 1.71 (s, 3 H), 1.70 (s, 3 H), 1.68 (s, 3 H), 1.64 (s, 3 H); ^{13}C NMR ($CDCl_3$, 150 MHz) δ 171.0, 170.43, 170.41, 170.38, 170.30, 170.2, 168.05, 167.99, 167.8, 167.3, 167.23, 167.18, 167.17, 163.0 (d, *J* = 247.5 Hz), 138.2, 138.11, 138.08, 138.04, 138.0, 137.4, 136.0 (d, *J* = 8.3 Hz), 134.39, 134.32, 134.25, 134.17, 131.6, 131.5, 131.4, 131.3, 131.1, 128.5, 128.2, 128.0, 127.91, 127.88, 127.87, 127.81, 127.6, 127.40, 127.37, 127.23, 127.21, 127.18, 127.15, 127.0, 126.9, 126.84, 126.80, 125.9 (d, *J* = 2.9 Hz), 123.69, 123.66, 123.58, 123.52, 123.4, 115.8 (d, *J* = 21.9 Hz), 96.5, 96.4, 95.9, 95.7, 82.7, 78.4, 77.3, 77.0, 76.8, 73.8, 73.57, 73.53, 73.47, 73.32, 73.27, 73.0, 72.8, 72.5, 72.2, 71.9, 71.7, 71.2, 71.1, 70.72, 70.69, 70.61, 69.8, 67.8, 67.5, 67.4, 55.3, 55.12, 55.08, 54.9, 53.7, 20.59, 20.56, 20.45, 20.43; HRMS (ESI) *m/z* calculated for $C_{167}H_{152}FKN_7O_{49}S$ $[M+K]^+$, 3128.8954; found, 3128.8948.

Buliding block **1b** (0.40 mmol, 220 mg) afforded oligosaccharides **2b** (*n* = 2, 60 μmol, 60 mg, 30%), **3b** (*n* = 3, 27 μmol, 40 mg, 20%), and **4b** (*n* = 4, 14 μmol, 26 mg, 14%) as white solids. Recovered yield of buliding block **1b** was 21% (47 mg, 83 μmol).

4-Chlorophenyl (3-*O*-acetyl-6-*O*-benzyl-2-deoxy-2-phthalimido-β-D-glucopyranosyl)-(1→4)-3-*O*-acetyl-6-*O*-benzyl-2-deoxy-2-phthalimido-1-thio-β-D-glucopyranoside (**2b**); TLC (Hexane:EtOAc 1:2): R_f 0.63. $[\alpha]_D$ = –8.62 (*c* = 1.3, $CHCl_3$, 27 °C); ^1H NMR ($CDCl_3$, 600 MHz) δ 7.86–7.78 (m, 4 H), 7.76–7.68 (m, 4 H), 7.35–7.26 (m, 10 H), 7.23–7.21 (m, 2 H), 7.10–7.07 (m, 2 H), 5.68 (dd, *J* = 10.2, 9.0 Hz, 1 H), 5.57 (dd, *J* = 10.8, 9.0 Hz, 1 H), 5.54 (d, *J* = 10.2 Hz, 1 H), 5.49

(d, *J* = 8.4 Hz, 1 H), 4.55 (d, *J* = 12.0 Hz, 1 H), 4.49 (d, *J* = 12.0 Hz, 1 H), 4.37 (d, *J* = 12.0 Hz, 1 H), 4.31 (d, *J* = 11.4 Hz, 1 H), 4.18 (*pseudo*-t, *J* = 10.2 Hz, 1 H), 4.11 (dd, *J* = 10.8, 8.4 Hz, 1 H), 4.04 (*pseudo*-t, *J* = 8.4 Hz, 1 H), 3.84–3.79 (m, 1 H), 3.76 (dd, *J* = 10.2, 4.2 Hz, 1 H), 3.66 (dd, *J* = 9.6, 4.8 Hz, 1 H), 3.56–3.51 (m, 2 H), 3.50–3.43 (m, 2 H), 2.95 (d, *J* = 3.6 Hz, 1 H), 1.89 (s, 3 H), 1.82 (s, 3 H).; ^{13}C NMR ($CDCl_3$, 150 MHz) δ 171.0, 170.0, 167.8, 167.2, 138.1, 137.3, 134.7, 134.6, 134.4, 134.3, 134.2, 131.7, 131.42, 131.39, 131.2, 129.4, 129.0, 128.5, 128.3, 127.9, 127.7, 127.5, 127.3, 123.7, 123.5, 97.2, 82.4, 78.5, 74.1, 73.6, 73.5, 73.2, 72.8, 72.3, 71.3, 69.9, 67.8, 54.9, 53.9, 20.61, 20.58; HRMS (ESI) *m/z* calculated for $C_{52}H_{47}ClN_2NaO_{14}S$ $[M+Na]^+$, 1013.2329; found, 1013.2300.

4-Chlorophenyl (3-*O*-acetyl-6-*O*-benzyl-2-deoxy-2-phthalimido-β-D-glucopyranosyl)-(1→4)-(3-*O*-acetyl-6-*O*-benzyl-2-deoxy-2-phthalimido-β-D-glucopyranosyl)-(1→4)-3-*O*-acetyl-6-*O*-benzyl-2-deoxy-2-phthalimido-1-thio-β-D-glucopyranoside (**3b**); TLC (Hexane:EtOAc 1:2): R_f 0.57. $[\alpha]_D$ = –17.5 (*c* = 1.3, $CHCl_3$, 27 °C).; ^{1}H NMR ($CDCl_3$, 600 MHz) δ 7.88–7.79 (m, 6 H), 7.76–7.67 (m, 6 H),7.36–7.32 (m, 2 H), 7.31–7.26 (m, 7 H), 7.25–7.20 (m, 5 H), 7.15 (*pseudo*-t, *J* = 7.8 Hz, 2 H), 7.10–7.08 (m, 2 H), 7.01 (*pseudo*-t, *J* = 7.2 Hz, 1 H), 5.60 (dd, *J* = 10.2, 9.0 Hz, 1 H), 5.55 (dd, *J* = 10.2, 8.4 Hz, 1 H), 5.52 (dd, *J* = 10.8, 9.0 Hz, 1 H), 5.50 (d, *J* = 10.8 Hz, 1 H), 5.38 (d, *J* = 8.4 Hz, 1 H), 5.27 (d, *J* = 8.4 Hz, 1 H), 4.53 (d, *J* = 11.4 Hz, 1 H), 4.48 (d, *J* = 12.0 Hz, 1 H), 4.43 (d, *J* = 11.4 Hz, 1 H), 4.42 (d, *J* = 11.4 Hz, 1 H), 4.38 (d, *J* = 11.4 Hz, 1 H), 4.32 (d, *J* = 11.4 Hz, 1 H), 4.17 (*pseudo*-t, *J* = 10.2 Hz, 1 H), 4.13 (*pseudo*-t, *J* = 9.0 Hz, 1 H), 4.07 (dd, *J* = 10.8, 8.4 Hz, 1 H), 4.03 (dd, *J* = 10.8, 8.4 Hz, 1 H), 4.00 (*pseudo*-t, *J* = 9.0 Hz, 1 H), 3.79 (td, *J* = 9.6, 3.0 Hz, 1 H), 3.72 (dd, *J* = 10.2, 4.2 Hz, 1 H), 3.63 (dd, *J* = 10.2, 4.8 Hz, 1 H), 3.55 (d, *J* = 9.6 Hz, 1 H), 3.48 (ddd, *J* = 9.6, 3.6, 1.2 Hz, 1 H), 3.45–3.41 (m, 2 H), 3.31–3.25 (m, 2 H), 3.11 (dd, *J* = 10.2, 1.2 Hz, 1 H), 2.89 (d, *J* = 3.6 Hz, 1 H), 1.88 (s, 3 H), 1.80 (s, 3 H), 1.71 (s, 3 H); ^{13}C NMR ($CDCl_3$, 150 MHz) δ 170.9, 170.2, 170.1, 168.1, 167.7, 167.3, 167.2, 138.2, 138.0, 137.4, 134.6, 134.5, 134.4, 134.3, 134.1, 131.6, 131.5, 131.44, 131.38, 131.1, 129.5, 128.9, 128.5, 128.2, 128.1, 127.9, 127.6, 127.42, 127.39, 127.3, 127.1, 123.7, 123.5, 123.4, 96.6, 96.5, 82.4, 78.5, 74.0, 73.6, 73.26, 73.23, 73.1, 73.0, 72.6, 72.3, 71.1, 71.4, 71.2, 69.9, 67.9, 67.4, 55.3, 54.9, 53.8, 20.61, 20.57, 20.46; HRMS (ESI) *m/z* calculated for $C_{75}H_{68}ClN_3NaO_{21}S$ $[M+Na]^+$, 1436.3647; found, 1436.3621.

4-Chlorophenyl (3-*O*-acetyl-6-*O*-benzyl-2-deoxy-2-phthalimido-β-D-glucopyranosyl)-(1→4)-(3-*O*-acetyl-6-*O*-benzyl-2-deoxy-2-phthalimido-β-D-glucopyranosyl)-(1→4)-(3-*O*-acetyl-6-*O*-benzyl-2-deoxy-2-phthalimido-β-D-glucopyranosyl)-(1→4)-3-*O*-acetyl-6-*O*-benzyl-2-deoxy-2-phthalimido-1-thio-β-D-glucopyranoside (**4b**); TLC (Hexane:EtOAc 1:2): R_f 0.50. $[\alpha]_D$ = –32.9 (*c* = 0.7, $CHCl_3$, 27 °C); ^{1}H NMR ($CDCl_3$, 600 MHz) δ 7.90–7.65 (m, 16 H), 7.36–7.32 (m, 2 H), 7.30–7.26 (m, 6 H), 7.25–7.18 (m, 8 H), 7.12–7.07 (m, 4 H), 6.99 (*pseudo*-t, *J* = 7.8 Hz, 2 H), 6.94 (*pseudo*-t, *J* = 7.8 Hz, 1 H), 6.70 (*pseudo*-t, *J* = 7.2 Hz, 1 H), 5.58 (dd, *J* = 10.2, 9.0 Hz, 1 H), 5.51–5.44 (m, 4

H), 5.34 (d, *J* = 8.4 Hz, 1 H), 5.21 (d, *J* = 8.4 Hz, 1 H), 5.18 (d, *J* = 8.4 Hz, 1 H), 4.52 (d, *J* = 12.0 Hz, 1 H), 4.47 (d, *J* = 11.4 Hz, 1 H), 4.45–4.37 (m, 4 H), 4.35 (d, *J* = 11.4 Hz, 1 H), 4.32 (d, *J* = 11.4 Hz, 1 H), 4.15 (*pseudo*-t, *J* = 10.2 Hz, 1 H), 4.09 (*pseudo*-t, *J* = 9.6 Hz, 1 H), 4.07–3.95 (m, 5 H), 3.77 (td, *J* = 9.0, 3.0 Hz, 1 H), 3.71 (dd, *J* = 10.2, 4.2 Hz, 1 H), 3.62 (dd, *J* = 10.2, 5.4 Hz, 1 H), 3.54 (d, *J* = 10.2 Hz, 1 H), 3.48–3.45 (m, 2 H), 3.41–3.38 (m, 2 H), 3.26–3.21 (m, 3 H), 3.01 (dd, *J* = 10.2, 1.8 Hz, 1 H), 2.86 (d, *J* = 3.0 Hz, 1 H), 2.79 (dd, *J* = 10.2, 1.2 Hz, 1 H), 1.87 (s, 3 H), 1.79 (s, 3 H), 1.73 (s, 3 H), 1.67 (s, 3 H); ^{13}C NMR ($CDCl_3$, 150 MHz) δ 171.9, 170.3, 170.2, 170.1, 168.1, 167.7, 167.23, 167.18, 138.20, 138.11, 138.09, 137.4, 134.58, 134.47, 134.37, 134.29, 134.17, 134.12, 131.6, 131.5, 131.4, 131.1, 129.6, 128.9, 128.5, 128.2, 128.1, 127.95, 127.92, 127.6, 127.4, 127.21, 127.18, 127.07, 126.97, 123.7, 123.54, 123.51, 123.36, 123.31, 96.52, 96.46, 95.9, 82.4, 78.5, 73.9, 73.7, 73.6, 73.3, 73.13, 73.07, 72.9, 72.5, 72.3, 72.2, 72.0, 71.6, 71.4, 71.2, 70.7, 69.9, 67.8, 67.4, 55.3, 55.2, 54.8, 53.7, 20.60, 20.57, 20.45; HRMS (ESI) *m/z* calculated for $C_{98}H_{89}ClN_4NaO_{28}S$ $[M+Na]^+$, 1859.4965; found, 1859.4932.

Buliding block **1c** (0.20 mmol, 110 mg) afforded oligosaccharides **2c** (*n* = 2, 17 μmol, 16 mg, 17%), **3c** (*n* = 3, 6.0 μmol, 8.3 mg, 9%), and **4c** (*n* = 4, 0.99 μmol, 1.8 mg, 2%) as white solids. Recovered yield of buliding block **1c** was 49% (53.3 mg, 97 μmol).

4-Methylphenyl (3-*O*-acetyl-6-*O*-benzyl-2-deoxy-2-phthalimido-β-D-glucopyranosyl)-(1→4)-3-*O*-acetyl-6-*O*-benzyl-2-deoxy-2-phthalimido-1-thio-β-D-glucopyranoside (**2c**); TLC (Hexane:EtOAc 1:2): R_f 0.50. $[\alpha]_D$ = –6.37 (*c* = 1.6, $CHCl_3$, 27 °C); ^{1}H NMR ($CDCl_3$, 600 MHz) δ 7.87–7.71 (m, 4 H), 7.75–7.71 (m, 2 H), 7.71–7.66 (m, 2 H), 7.35–7.27 (m, 8 H), 7.23 (d, *J* = 7.8 Hz, 2 H), 7.21 (d, *J* = 8.4 Hz, 2 H), 6.94 (d, *J* = 7.8 Hz, 2 H), 5.68 (dd, *J* = 10.2, 9.0 Hz, 1 H), 5.57 (dd, *J* = 10.8, 9.0 Hz, 1 H), 5.52 (d, *J* = 10.8 Hz, 1 H), 5.45 (d, *J* = 8.4 Hz, 1 H), 4.54 (d, *J* = 12.0 Hz, 1 H), 4.49 (d, *J* = 12.0 Hz, 1 H), 4.37 (d, *J* = 12.0 Hz, 1 H), 4.32 (d, *J* = 12.0 Hz, 1 H), 4.18 (*pseudo*-t, *J* = 10.2 Hz, 1 H), 4.11 (dd, *J* = 10.8, 8.4 Hz, 1 H), 4.04 (*pseudo*-t, *J* = 9.0 Hz, 1 H), 3.84–3.78 (m, 1 H), 3.75 (dd, *J* = 9.6, 3.6 Hz, 1 H), 3.65 (dd. *J* = 10.2, 5.4 Hz, 1 H), 3.54–3.50 (m, 2 H), 3.48–3.43 (m, 2 H), 2.96 (d, *J* = 3.0 Hz, 1 H), 2.25 (s, 3 H), 1.88 (s, 3 H), 1.82 (s, 3 H).; ^{13}C NMR ($CDCl_3$, 150 MHz) δ 170.9, 170.0, 167.8, 167.3, 138.4, 138.2, 137.4, 134.3, 134.2, 134.1, 133.8, 131.7, 131.4, 131.2, 129.5, 128.5, 128.2, 127.9, 127.7, 127.4, 127.1, 123.6, 123.5, 97.2, 82.7, 78.6, 74.1, 73.6, 73.5, 73.2, 72.7, 72.5, 71.3, 70.0, 67.8, 54.9, 54.0, 21.1, 20.6; HRMS (ESI) *m/z* calculated for $C_{53}H_{50}N_2NaO_{14}S$ $[M+Na]^+$, 993.2875; found, 993.2875.

4-Methylphenyl (3-*O*-acetyl-6-*O*-benzyl-2-deoxy-2-phthalimido-β-D-glucopyranosyl)-(1→4)-(3-*O*-acetyl-6-*O*-benzyl-2-deoxy-2-phthalimido-β-D-glucopyranosyl)-(1→4)-3-*O*-acetyl-6-*O*-benzyl-2-deoxy-2-phthalimido-1-thio-β-D-glucopyranoside (**3c**); TLC (Hexane:EtOAc 1:2): R_f 0.57. $[\alpha]_D$ = –17.9 (*c* = 1.7, $CHCl_3$, 27 °C); ^{1}H NMR ($CDCl_3$, 600 MHz) δ 7.89–7.77 (m, 6 H), 7.76–7.66 (m, 6 H), 7.36–7.32 (m, 2 H), 7.31–7.27 (m, 5 H), 7.24–7.20 (m, 7 H), 7.13 (*pseudo*-t, *J* = 7.8 Hz, 2 H), 6.99 (*pseudo*-t, *J* = 7.2 Hz, 1 H), 6.94 (d, *J* = 7.8 Hz, 2 H), 5.60 (dd, *J* = 10.2, 9.0 Hz, 1 H), 5.55 (dd, *J* = 10.8, 9.0 Hz, 1 H), 5.51 (dd, *J* = 10.2, 8.4 Hz, 1 H), 5.48 (d, *J* = 10.8 Hz, 1 H), 5.38 (d, *J* = 8.4 Hz, 1 H), 5.27 (d, *J* = 7.8 Hz, 1 H), 4.53 (d, *J* = 11.4 Hz, 1 H), 4.48 (d, *J* = 12.0 Hz, 1 H), 4.44–4.38 (m, 3 H), 4.32 (d, *J* = 11.4 Hz, 1 H), 4.17 (*pseudo*-t, *J* = 10.8 Hz, 1 H), 4.12 (*pseudo*-t, *J* = 9.0 Hz, 1 H), 4.07 (dd, *J* = 10.8, 8.4 Hz, 1 H), 4.03–3.99 (m, 2 H), 3.79 (td, *J* = 9.0, 3.0 Hz, 1 H), 3.72 (dd, *J* = 10.2, 4.2 Hz, 1 H), 3.63 (dd, *J* = 9.6, 4.8 Hz, 1 H), 3.55 (d, *J* = 10.2 Hz, 1 H), 3.46 (dd, *J* = 10.2, 3.6 Hz, 1 H), 3.44–3.39 (m, 2 H), 3.31–3.25 (m, 2 H), 3.09 (dd, *J* = 10.2, 1.2 Hz, 1 H), 2.88 (d, *J* = 3.6 Hz, 1 H), 2.24 (s, 3 H), 1.88 (s, 3 H), 1.80 (s, 3 H), 1.70 (s, 3 H); ^{13}C NMR ($CDCl_3$, 150 MHz) δ 171.0, 170.2, 170.1, 168.1, 167.7, 167.32, 167.26, 138.3, 138.1, 137.4, 134.3, 134.09, 134.05, 133.7, 131.7, 131.54, 131.45, 131.3, 129.5, 128.5, 128.2, 128.0, 127.9, 127.6, 127.41, 127.39, 127.3, 127.1, 123.60, 123.55, 123.47, 123.3, 96.6, 96.5, 82.9, 78.6, 74.0, 73.6, 73.26, 73.21, 73.16, 73.09, 72.6, 72.3, 71.9, 71.4, 71.3, 69.9, 67.9, 67.3, 55.3, 54.9, 53.9, 21.1, 20.61, 20.58, 20.49; HRMS (ESI) *m/z* calculated for $C_{76}H_{71}N_3NaO_{21}S$ $[M+Na]^+$, 1416.4193; found, 1416.4163.

4-Methylphenyl (3-*O*-acetyl-6-*O*-benzyl-2-deoxy-2-phthalimido-β-D-glucopyranosyl)-(1→4)-(3-*O*-acetyl-6-*O*-benzyl-2-deoxy-2-phthalimido-β-D-glucopyranosyl)-(1→4)-(3-*O*-acetyl-6-*O*-benzyl-2-deoxy-2-phthalimido-β-D-glucopyranosyl)-(1→4)-3-*O*-acetyl-6-*O*-benzyl-2-deoxy-2-phthalimido-1-thio-β-D-glucopyranoside (**4c**); TLC (Hexane:EtOAc 1:2): R_f 0.48. $[\alpha]_D$ = –24.3 (*c* = 0.6, $CHCl_3$, 27 °C); ^{1}H NMR ($CDCl_3$, 600 MHz) δ 7.90–7.84 (m, 4 H), 7.83–7.74 (m, 8 H), 7.73–7.65 (m, 4 H), 7.37–7.26 (m, 8 H), 7.25–7.18 (m, 8 H), 7.09 (d, *J* = 6.0 Hz, 1 H), 7.08 (d, *J* = 7.8 Hz, 1 H), 6.99 (*pseudo*-t, *J* = 7.8 Hz, 2 H), 6.95–6.89 (m, 3 H), 6.69 (*pseudo*-t, *J* = 7.2 Hz, 1 H), 5.58 (*pseudo*-t, *J* = 9.6 Hz, 1 H), 5.52–5.48 (m, 2 H), 5.45 (d, *J* = 7.8 Hz, 2 H), 5.34 (d, *J* = 8.4 Hz, 1 H), 5.21 (d, *J* = 8.4 Hz, 1 H), 5.18 (d, *J* = 8.4 Hz, 1 H), 4.52 (d, *J* = 12.0 Hz, 1 H), 4.47 (d, *J* = 11.4 Hz, 1 H), 4.43 (d, *J* = 11.4 Hz, 1 H), 4.42 (d, *J* = 11.4 Hz, 1 H), 4.41–4.38 (m, 2 H), 4.35 (d, *J* = 11.4 Hz, 1 H), 4.32 (d, *J* = 11.4 Hz, 1 H), 4.16 (*pseudo*-t, *J* = 10.2 Hz, 1 H), 4.12–4.02 (m, 3 H), 4.01–3.95 (m, 3 H), 3.77 (*pseudo*-t, *J* = 9.0 Hz, 1 H), 3.75–3.69 (m, 1 H), 3.62 (dd, *J* = 9.6, 4.8 Hz, 1 H), 3.54 (d, *J* = 10.8 Hz, 1 H), 3.46–3.42 (m, 2 H), 3.41–3.37 (m, 2 H), 3.26–3.19 (m, 3 H), 2.99 (d, *J* = 10.2 Hz, 1 H), 2.87 (s, 1 H), 2.78 (d, *J* = 9.6 Hz, 1 H), 2.24 (s, 3 H), 1.87 (s, 3 H), 1.79 (s, 3 H), 1.73 (s, 3 H), 1.67 (s, 3 H); ^{13}C NMR ($CDCl_3$, 150 MHz) δ 171.0, 170.3, 170.2, 170.1, 168.1, 167.7, 167.3, 138.4, 138.3, 138.2, 138.1, 137.4, 134.29, 134.27, 134.23, 134.19, 134.17, 134.0, 133.7, 131.7, 131.6, 131.5, 131.49, 131.46, 131.42, 131.39, 131.27, 129.5, 128.5, 128.2, 127.99, 127.96, 127.92, 127.6, 127.41, 127.38,

127.38, 127.2, 127.10, 127.08, 126.94, 123.67, 123.63, 123.60, 123.57, 123.51, 123.48, 123.39, 123.30, 96.51, 96.48, 95.9, 82.9, 78.6, 73.9, 73.7, 73.6, 73.3, 73.14, 73.13, 72.9, 72.5, 72.26, 72.24, 72.0, 71.8, 71.4, 70.7, 70.0, 67.9, 67.5, 55.3, 55.2, 54.9, 53.9, 21.1, 20.60, 20.57, 20.48, 20.45; HRMS (ESI) *m/z* calculated for $C_{99}H_{92}KN_4O_{28}S$ $[M+K]^+$, 1855.5250; found, 1855.5226.

Buliding block **1d** (0.2 mmol, 114 mg) afforded oligosaccharides **2d** (*n* = 2, 23 μmol, 22.8 mg, 23%), **3d** (*n* = 3, 6.7 μmol, 9.5 mg, 10%), **4d** (*n* = 4, 4.3 μmol, 8.0 mg, 9%), and **5d** (*n* = 5, 2.6 μmol, 5.8 mg, 6%) as white solids. Recovered yield of buliding block **1d** was 37% (42 mg, 74 μmol).

2,4-Difluorophenyl (3-*O*-acetyl-6-*O*-benzyl-2-deoxy-2-phthalimido-β-D-glucopyranosyl)-(1→4)-3-*O*-acetyl-6-*O*-benzyl-2-deoxy-2-phthalimido-1-thio-β-D-glucopyranoside (**2d**); TLC (Hexane:EtOAc 1:2): R_f 0.60. $[\alpha]_D$ = –4.06 (*c* = 1.4, $CHCl_3$, 27 °C); ^1H NMR ($CDCl_3$, 600 MHz) δ 7.86–7.67 (m, 8 H), 7.46 (td, *J* = 8.4, 6.0 Hz, 1 H), 7.36–7.28 (m, 8 H), 7.20 (d, *J* = 8.4 Hz, 2 H), 6.70 (td, *J* = 8.4, 2.4 Hz, 1 H), 6.64 (td, *J* = 8.4, 2.4 Hz, 1 H), 5.66 (*pseudo*-t, *J* = 9.6 Hz, 1 H), 5.57 (dd, *J* = 10.8, 9.6 Hz, 1 H), 5.52 (d, *J* = 10.2 Hz, 1 H), 5.45 (d, *J* = 8.4 Hz, 1 H), 4.54 (d, *J* = 12.0 Hz, 1 H), 4.49 (d, *J* = 11.4 Hz, 1 H), 4.35 (d, *J* = 12.0 Hz, 1 H), 4.32 (d, *J* = 11.4 Hz, 1 H), 4.14−4.08 (m, 2 H), 4.03 (*pseudo*-t, *J* = 9.0 Hz, 1 H), 3.82 (td, *J* = 10.8, 3.6 Hz, 1 H), 3.75 (dd, *J* = 9.6, 3.6 Hz, 1 H), 3.66 (dd, *J* = 10.2, 5.4 Hz, 1 H), 3.53–3.50 (m, 2 H), 3.48 (dd, *J* = 9.6, 4.8 Hz, 1 H), 3.45 (dd, *J* = 11.4, 3.6 Hz, 1 H), 2.96 (d, *J* = 3.0 Hz, 1 H), 1.88 (s, 3 H), 1.82 (s, 3 H).; ^{13}C NMR ($CDCl_3$, 150 MHz) δ 171.0, 170.0, 167.8, 167.3, 163.6 (dd, *J* = 250.7, 11.4 Hz), 162.8 (dd, *J* = 248.6, 12.3 Hz), 138.2, 137.8 (d, *J* = 9.3 Hz), 137.4, 134.4, 134.25, 134.17, 131.6, 131.4, 131.2, 128.5, 128.2, 127.9, 127.7, 127.5, 127.3, 123.7, 123.5, 112.9 (dd, *J* = 18.5, 4.1 Hz), 111.9 (dd, *J* = 21.3, 3.6 Hz), 104.4 (t, *J* = 26.3 Hz), 97.3, 82.0, 78.6, 74.0, 73.6, 73.5, 73.2, 72.8, 72.3, 71.2, 69.9, 67.8, 54.9, 53.8, 20.60, 20.58; HRMS (ESI) *m/z* calculated for $C_{52}H_{46}F_2KN_2O_{14}S$ $[M+K]^+$, 1031.2269; found, 1031.2269.

2,4-Difluorophenyl (3-*O*-acetyl-6-*O*-benzyl-2-deoxy-2-phthalimido-β-D-glucopyranosyl)-(1→4)-(3-*O*-acetyl-6-*O*-benzyl-2-deoxy-2-phthalimido-β-D-glucopyranosyl)-(1→4)-3-*O*-acetyl-6-*O*-benzyl-2-deoxy-2-phthalimido-1-thio-β-D-glucopyranoside (**3d**); TLC (Hexane:EtOAc 1:2): R_f 0.55. $[\alpha]_D$ = –18.1 (*c* = 1.7, $CHCl_3$, 27 °C); ^1H NMR ($CDCl_3$, 600 MHz) δ 7.88–7.67 (m, 12 H), 7.46 (td, *J* = 8.4, 6.6 Hz, 1 H), 7.37–7.19 (m, 12 H), 7.15 (*pseudo*-t, *J* = 7.8 Hz, 2 H), 7.02 (*pseudo*-t, *J* = 7.2 Hz, 1 H), 6.71 (td, *J* = 8.4, 2.4 Hz, 1 H), 6.65 (td, *J* = 8.4, 2.4 Hz, 1 H), 5.58 (dd, *J* = 9.6, 9.0 Hz, 1 H), 5.55 (dd, *J* = 10.2, 8.4 Hz, 1 H), 5.51 (dd, *J* = 10.8, 9.0 Hz, 1 H), 5.47 (d, *J* = 10.2 Hz, 1 H), 5.38 (d, *J* = 8.4 Hz, 1 H), 5.27 (d, *J* = 8.4 Hz, 1 H), 4.53 (d, *J* = 12.0 Hz, 1 H), 4.48 (d, *J* = 11.4 Hz, 1 H), 4.45–4.40 (m, 2 H), 4.38 (d, *J* = 11.4 Hz, 1 H), 4.31 (d, *J* = 12.0 Hz, 1 H), 4.13 (*pseudo*-t, *J* = 9.6 Hz, 1 H), 4.11–4.05 (m, 2 H), 4.02 (dd, *J* = 10.8, 8.4 Hz, 1 H), 4.00 (*pseudo*-t, *J* = 9.6 Hz, 1 H), 3.79 (td, *J* = 9.0, 3.0 Hz, 1 H), 3.72 (dd, *J* = 10.2, 4.2 Hz, 1 H), 3.63 (dd, *J* = 10.2, 4.8 Hz, 1 H), 3.54 (d, *J* =

10.8 Hz, 1 H), 3.48–3.40 (m, 3 H), 3.33–3.25 (m, 2 H), 3.11 (dd, *J* = 9.6, 1.2 Hz, 1 H), 2.88 (d, *J* = 3.6 Hz, 1 H), 1.88 (s, 3 H), 1.80 (s, 3 H), 1.71 (s, 3 H); ^{13}C NMR ($CDCl_3$, 150 MHz) δ 170.9, 170.15, 170.07, 168.1, 167.7, 167.29, 167.24, 163.5 (dd, *J* = 250.5, 11.0 Hz), 162.6 (dd, *J* = 248.3, 12.2 Hz), 138.2, 138.0, 137.7 (d, *J* = 9.9 Hz), 137.4, 134.34, 134.27, 134.12, 131.61, 131.52, 131.44, 131.38, 131.18, 128.5, 128.24, 128.09, 127.92, 127.64, 127.40, 127.31, 127.25, 127.14, 123.61, 123.54, 123.46, 123.35, 113.1 (dd, *J* = 17.6, 3.3 Hz), 111.9 (dd, *J* = 21.8, 3.3 Hz), 104.4 (t, *J* = 26.3 Hz), 96.6, 96.5, 82.1, 78.6, 74.0, 73.6, 73.2, 73.0, 72.6, 72.3, 71.7, 71.3, 71.2, 69.9, 67.8, 67.3, 55.3, 54.9, 53.8, 20.60, 20.57, 20.45; HRMS (ESI) *m/z* calculated for $C_{75}H_{67}F_2KN_3O_{21}S$ $[M+K]^+$, 1454.3587; found, 1454.3563.

2,4-Difluorophenyl (3-*O*-acetyl-6-*O*-benzyl-2-deoxy-2-phthalimido-β-D-glucopyranosyl)-(1→4)-(3-*O*-acetyl-6-*O*-benzyl-2-deoxy-2-phthalimido-β-D-glucopyranosyl)-(1→4)-(3-*O*-acetyl-6-*O*-benzyl-2-deoxy-2-phthalimido-β-D-glucopyranosyl)-(1→4)-3-*O*-acetyl-6-*O*-benzyl-2-deoxy-2-phthalimido-1-thio-β-D-glucopyranoside (**4d**); TLC (Hexane:EtOAc 1:2): R_f 0.47. $[\alpha]_D$ = –5.00 (*c* = 1.4, $CHCl_3$, 27 °C); ^{1}H NMR ($CDCl_3$, 600 MHz) δ 7.89–7.65 (m, 16 H), 7.46 (td, *J* = 7.8, 6.6 Hz, 1 H), 7.38–7.18 (m, 15 H), 7.10 (*pseudo*-t, *J* = 7.8 Hz, 1 H), 6.99 (*pseudo*-t, *J* = 7.8 Hz, 2 H), 6.95 (*pseudo*-t, *J* = 7.2 Hz, 1 H), 6.72–6.68 (m, 2 H), 6.65 (td, *J* = 8.4, 2.4 Hz, 1 H), 5.57 (*pseudo*-t, *J* = 10.2 Hz, 1 H), 5.51–5.43 (m, 4 H), 5.34 (d, *J* = 7.8 Hz, 1 H), 5.21 (d, *J* = 8.4 Hz, 1 H), 5.18 (d, *J* = 8.4 Hz, 1 H), 4.52 (d, *J* = 12.0 Hz, 1 H), 4.47 (d, *J* = 11.4 Hz, 1 H), 4.44–4.34 (m, 5 H), 4.32 (d, *J* = 11.4 Hz, 1 H), 4.13–3.95 (m, 7 H), 3.77 (td, *J* = 9.6, 3.0 Hz, 1 H), 3.71 (dd, *J* = 9.6, 3.6 Hz, 1 H), 3.62 (dd, *J* = 10.2, 4.8 Hz, 1 H), 3.52 (d, *J* = 10.2 Hz, 1 H), 3.49–3.35 (m, 4 H), 3.29–3.19 (m, 3 H), 3.02 (d, *J* = 10.2 Hz, 1 H), 2.87 (d, *J* = 3.0 Hz, 1 H), 2.79 (d, *J* = 10.2 Hz, 1 H), 1.87 (s, 3 H), 1.79 (s, 3 H), 1.73 (s, 3 H), 1.67 (s, 3 H); ^{13}C NMR ($CDCl_3$, 150 MHz) δ 170.9, 170.3, 170.2, 168.1, 167.7, 167.2, 163.5 (dd, *J* = 249.3, 9.8 Hz), 162.6 (dd, *J* = 248.4, 12.2 Hz), 138.2, 138.11, 138.09, 137.6 (d, *J* = 8.7 Hz), 137.4, 134.4, 134.3, 134.2, 134.1, 131.6, 131.44, 131.35, 131.2, 128.5, 128.2, 128.03, 127.96, 127.91, 127.6, 127.4, 127.3, 127.2, 127.1, 127.0, 123.61, 123.57, 123.45, 123.36, 113.1 (dd, *J* = 18.6, 4.4 Hz), 111.9 (dd, *J* = 21.9, 4.4 Hz), 104.4 (t, *J* = 26.3 Hz), 96.54, 96.46, 95.9, 82.1, 78.6, 73.9, 73.7, 73.6, 73.3, 73.2, 73.0, 72.9, 72.5, 72.2, 72.0, 71.7, 71.4, 71.2, 70.7, 69.9, 67.79, 67.45, 67.43, 55.3, 55.1, 54.9, 53.7, 20.60, 20.56, 20.44; HRMS (ESI) *m/z* calculated for $C_{98}H_{88}F_2N_4NaO_{28}S$ $[M+Na]^+$, 1861.5166; found, 1861.5137.

2,4-Difluorophenyl (3-*O*-acetyl-6-*O*-benzyl-2-deoxy-2-phthalimido-β-D-glucopyranosyl)-(1→4)-(3-*O*-acetyl-6-*O*-benzyl-2-deoxy-2-phthalimido-β-D-glucopyranosyl)-(1→4)-(3-*O*-acetyl-6-*O*-benzyl-2-deoxy-2-phthalimido-β-D-glucopyranosyl)-(1→4)-(3-*O*-acetyl-6-*O*-benzyl-2-deoxy-2-phthalimido-β-D-glucopyranosyl)-(1→4)-3-*O*-acetyl-6-*O*-benzyl-2-deoxy-2-phthalimido-1-thio-β-D-glucopyranoside (**5d**); TLC (Hexane:EtOAc 1:2): R_f 0.40. $[\alpha]_D$ = –27.1 (*c* = 1.0, $CHCl_3$, 30 °C); ^{1}H NMR ($CDCl_3$, 600 MHz) δ 7.90–7.64 (m, 20 H), 7.45 (td, *J* = 7.8, 6.0 Hz, 1 H), 7.36–7.26 (m, 8 H), 7.25–7.15 (m, 9 H), 7.09 (*pseudo*-t, *J* = 7.2 Hz, 2 H), 6.97–6.90 (m, 4 H), 6.70 (td, *J* = 8.4, 2.4 Hz, 1 H), 6.66–6.61 (m, 2 H), 6.57 (*pseudo*-t, *J* = 7.8 Hz, 1 H), 5.57 (*pseudo*-t, *J* = 9.6 Hz, 1 H) 5.50–5.41 (m, 4 H), 5.37 (dd, *J* = 10.8, 9.6 Hz, 1 H), 5.32 (d, *J* = 8.4 Hz, 1 H), 5.19 (d, *J* = 8.4 Hz, 1 H), 5.12 (*pseudo*-t, *J* = 8.4 Hz, 2 H), 4.51 (d, *J* = 11.4 Hz, 1 H), 4.47 (d, *J* = 12.0 Hz, 1 H), 4.45–4.30 (m, 8 H), 4.10–3.91 (m, 10 H), 3.77 (td, *J* = 9.6, 3.6 Hz, 1 H), 3.71 (dd, *J* = 9.6, 3.6 Hz, 1 H), 3.62 (dd, *J* = 10.2, 4.8 Hz, 1 H), 3.51 (d, *J* = 10.8 Hz, 1 H), 3.46–3.36 (m, 4 H), 3.23–3.13 (m, 4 H), 3.00 (d, *J* = 10.2 Hz, 1 H), 2.85 (d, *J* = 2.4 Hz, 1 H), 2.72 (d, *J* = 9.0 Hz, 1 H), 2.61 (d, *J* = 9.0 Hz, 1 H), 1.88 (s, 3 H), 1.78 (s, 3 H), 1.73 (s, 3 H), 1.70 (s, 3 H), 1.65 (s, 3 H); ^{13}C NMR ($CDCl_3$, 150 MHz) δ 170.9, 170.35, 170.32, 170.18, 170.15, 168.0, 167.7, 167.22, 167.16, 163.5 (dd, *J* = 250.7, 11.3 Hz), 162.6 (dd, *J* = 249.1, 11.9 Hz), 138.2, 138.10, 138.08, 138.05, 137.6 (d, *J*= 9.0 Hz), 137.4, 134.3, 134.24, 134.22, 134.15, 134.09, 131.55, 131.48, 131.4, 131.3, 131.1, 128.46, 128.18, 128.00, 127.87, 127.86, 127.6, 127.4, 127.3, 127.19, 127.14, 127.04, 126.89, 126.84, 123.7, 123.6, 123.5, 123.4, 123.3, 113.1 (dd, *J* = 18.3, 3.6 Hz), 111.9 (dd, *J* = 22.4, 3.3 Hz), 104.3 (t, *J* = 25.5 Hz), 96.5, 96.4, 95.9, 95.7, 82.1, 78.5, 73.8, 73.55, 73.52, 73.2, 73.0, 72.8, 72.5, 72.2, 71.97, 71.95, 71.6, 71.2, 70.7, 70.6, 69.8, 67.7, 67.5, 67.4, 55.2, 55.0, 54.8, 53.7, 20.55, 20.52, 20.4; HRMS (ESI) *m/z* calculated for $C_{121}H_{109}F_2KN_5O_{35}S$ $[M+K]^+$, 2300.6223; found, 2300.6287.

3. Optimization of electricity and electrolyte

Table S1. Optimization of electricity of electrochemical polyglycosylation.

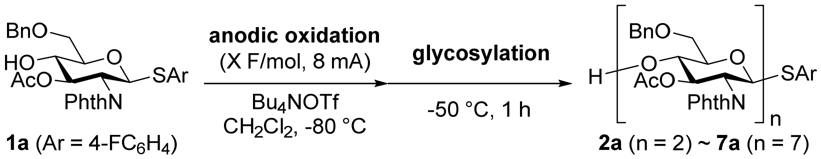

entry	electricity (F/mol)	yield of oligosaccharides **2a** (n = 2)–**7a** (n = 7)							
		**2a**	**3a**	**4a**	**5a**	**6a**	**7a**	Conv.	total
1	0.3	25%	3%	trace	—	—	—	53%	28%
2	0.4	32%	20%	2%	—	—	—	63%	54%
3	0.5	31%	16%	7%	3%	2%	—	70%	59%
4	0.6	33%	19%	8%	—	—	—	quant	60%

Table S2. Optimization of electrolyte of electrochemical polyglycosylation.

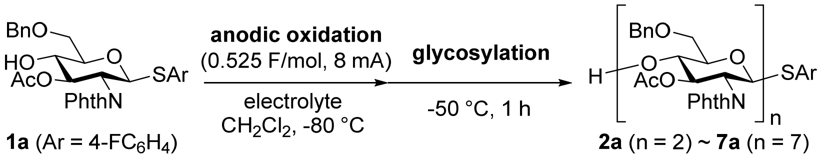

entry	electrolyte	yield of oligosaccharides **2a** (n = 2)–**7a** (n = 7)							
		**2a**	**3a**	**4a**	**5a**	**6a**	**7a**	conv.	total
1	Bu_4NOTf	27%	19%	10%	3%	1%	trace	73%	61%
2	Et_4NOTf	41%	14%	4%	trace	—	—	79%	59%
3	[BMPy]OTf	33%	14%	6%	1%	—	—	79%	54%

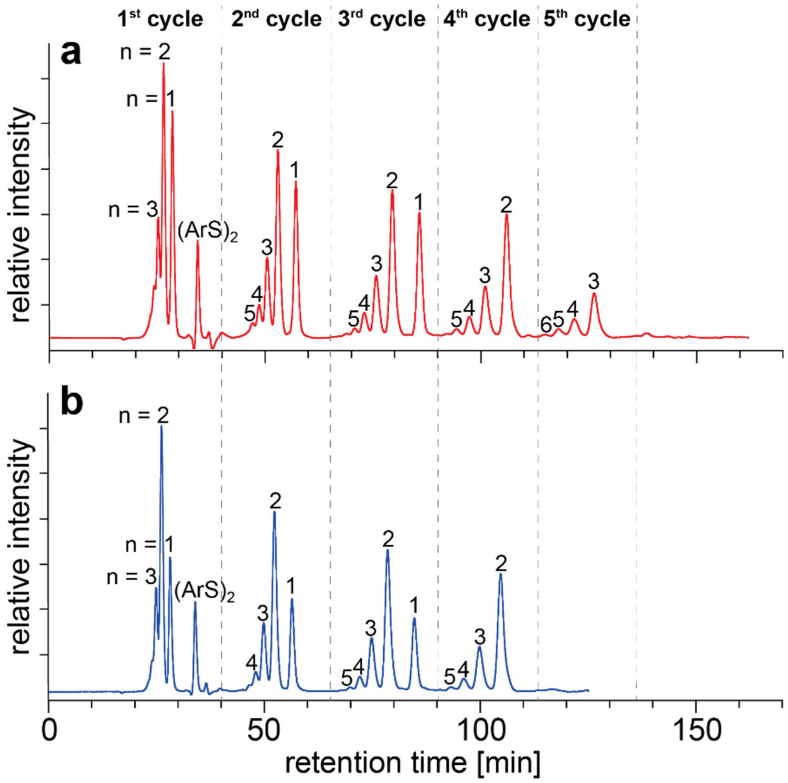

Figure S1. Preparative recycling GPC traces of protected oligosaccharides **2a** (n = 2)–**6a** (n = 6). (a) Bu_4NOTf as electrolyte (Table S2, entry 1). (b) Et_4NOTf as electrolyte (Table S2, entry 2).

4. Influence of reaction parameters

Table S3. Effect of the anomeric leaving group on the yield of oligosaccharides (see Figure 2).

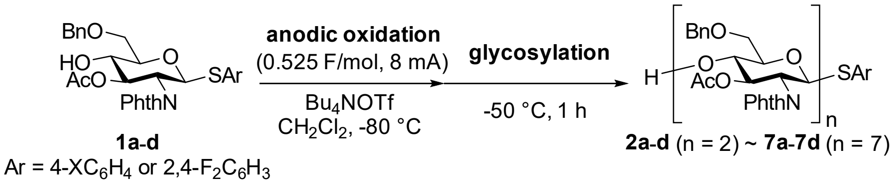

entry	X	oxidation potential	yield of oligosaccharides **2a** (n = 2)–**7a** (n = 7)							
			**2a**	**3a**	**4a**	**5a**	**6a**	**7a**	conv.	total
1	F	1.70 V	27%	19%	11%	3%	1%	trace	73%	61%
2	Cl	1.68 V	30%	20%	14%	—	—	—	79%	64%
3	methyl	1.47 V	17%	9%	2%	—	—	—	51%	28%
4	difluoro	1.73 V	23%	10%	7%	6%	—	—	63%	46%

Table S4. Influence of the glycosylation temperature on the yield of oligosaccharides (see Figure 3).

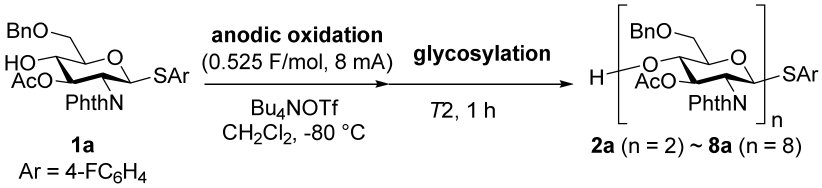

entry	*T*2	yield of oligosaccharides **2a** (*n* = 2)–**7a** (*n* = 7)							
		**2a**	**3a**	**4a**	**5a**	**6a**	**7a**	conv.	total
1	−30	28%	18%	8%	5%	3%	1%	78%	63%
2	−40	26%	16%	9%	7%	4%	1%	84%	63%
3	−50	27%	19%	11%	3%	1%	trace	77%	61%
4	−60	42%	14%	5%	2%	2%	—	79%	65%
5	−80	34%	10%	4%	1%	—	—	63	49%

Table S5. Influence of the temperature of anodic oxidation and glycosylation (see Figure 4).

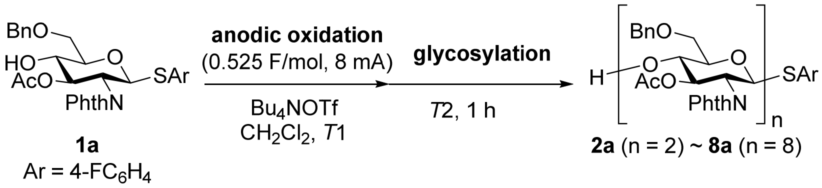

entry	*T*1	*T*2	yield of oligosaccharides **2a** (*n* = 2)–**7a** (*n* = 7)							
			**2a**	**3a**	**4a**	**5a**	**6a**	**7a**	conv.	total
1	-30	−30	17%	12%	11%	6%	4%	1%	71%	51%
2	-60	−30	27%	24%	11%	6%	2%	—	82%	70%
3	-60	−60	18%	19%	12%	5%	4%	1%	67%	59%
4	-80	−60	42%	14%	5%	2%	2%	—	79%	65%

Table S6. Influence of the number of cycles on the yield of longer oligosaccharides (see Figure 9).

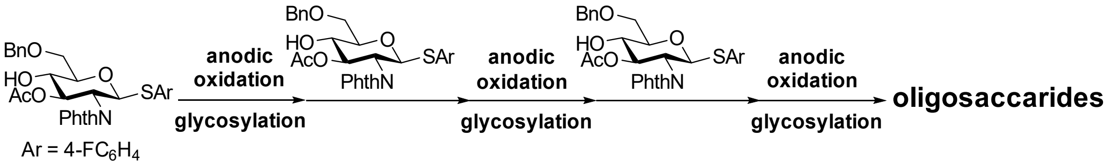

entry	cycles	yield of oligosaccharides **2a** (n = 2)–**8a** (n = 8)							
		**2a**	**3a**	**4a**	**5a**	**6a**	**7a**	**8a**	total
1	1st	27%	24%	11%	6%	2%	—	—	70%
2	2nd	29%	20%	11%	7%	5%	3%	—	75%
3	3rd	22%	17%	11%	8%	6%	5%	3%	72%

5. Measurement of oxidation potential of oligosaccharides

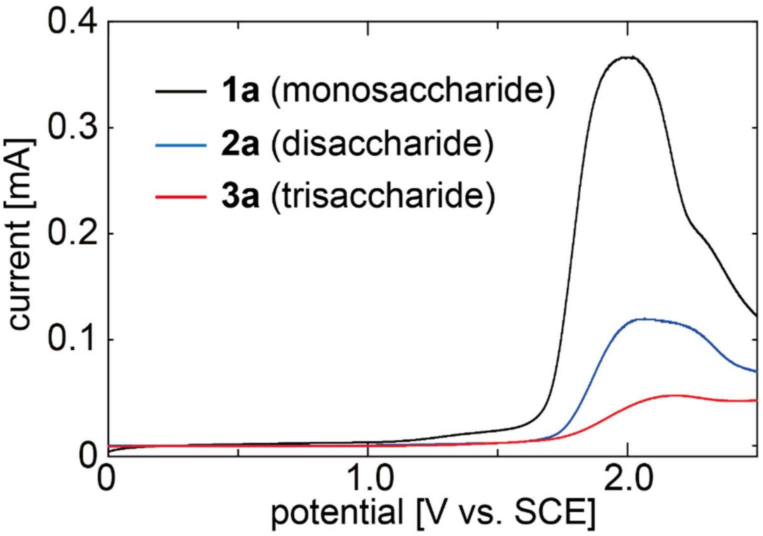

Figure S2. Linear sweep voltammetry of monosaccharide **1a** and oligosaccharides **2a** and **3a** measured by rotating disk electrode.

6. Electrochemical dimerization of tetrasaccharide

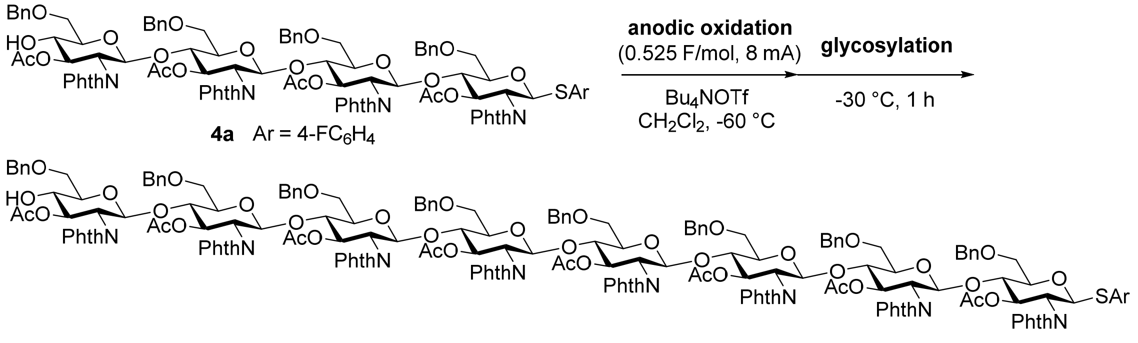

8a (trace) recovery of **4a** (64%)

The electrochemical dimerization of tetrasaccharide **4a** was carried out an H-type divided cell (4G glass filter). The cell had a carbon felt anode (Nippon Carbon JF-20-P7) and platinum square plate (20 mm×20 mm). Tetrasaccharide **4a** (0.1 mmol, 182 mg), Bu_4NOTf (0.5 mmol, 196 mg), and CH_2Cl_2 (5 mL) were added to the anodic chamber. Trifluoromethanesulfonic acid (0.1 mmol, 9 µL), Bu_4NOTf (0.5 mmol, 196 mg), and CH_2Cl_2 (5 mL) were added to the cathodic chamber. The constant current (6 mA (current density: 2.0 mA/cm^2), 29 V (electrode distance: 4.5 cm)) was employed at –60 °C with magnetic stirring until 0.52 F/mol of the electricity was consumed. After the electrolysis, the reaction was kept stirring at –30 °C for 1 h. After that, triethylamine (0.2 mL) was added to both chambers. The solution in both chambers was collected in eggplant flask, and the solvent was removed under reduced pressure. The mixture was dissolved in EtOAc and washed with water (3 times) and brine, respectively. The solution was dried over Na_2SO_4, and the solvent was removed under reduced pressure. The crude product was purified with preparative-GPC to afford octasaccharides **8a** (*n* = 8, trace), and recovered yield of tetrasaccharides **4a** (*n* = 4, 0.6422 mmol, 117 mg, 64%) as white solids.

7. Protocol modification of electrochemical polyglycosylation

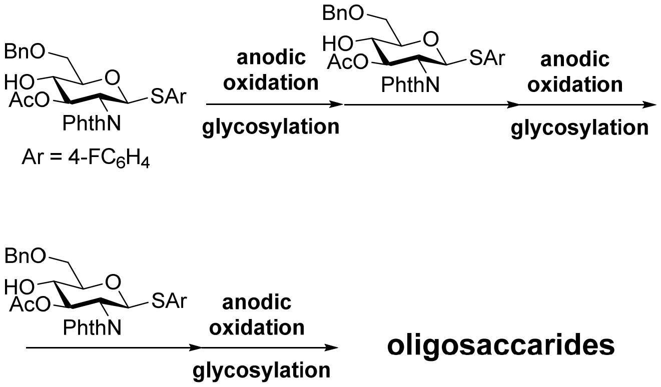

The electrochemical polymerization synthesis of linear oligosaccharides (**2a**–**8a**) was carried out in an H-type divided cell (4G glass filter). The cell had a carbon felt anode (Nippon Carbon JF-20-P7) and platinum square plate (20 mm × 20 mm). Building block **1a** (0.200 mmol, 109 mg), Bu_4NOTf (1.00 mmol, 393 mg), and CH_2Cl_2 (10 mL) were added to the anodic chamber. Trifluoromethanesulfonic acid (0.200 mmol, 18 μL), Bu_4NOTf (1.00 mmol, 393 mg), and CH_2Cl_2 (10 mL) were added to the cathodic chamber. The constant current (8 mA (current density: 2.0 mA/cm^2), 53 V (electrode distance: 4.5 cm)) was employed at −60 °C with magnetic stirring until 0.52 F/mol of the electricity was consumed. After the electrolysis, the reaction was kept stirring at −30 °C for 1 h. After that, building block **1a** (0.400 mmol, 218 mg) dissolved in CH_2Cl_2 (2.0 mL) was subsequently added by the syringe (1.0 mL (0.200 mmol) for one cycle) at −30 °C. The reaction temperature was cooled down to −60 °C and the next cycle started. After the 2nd cycle, triethylamine (0.3 mL) was added to both chambers. The solution in both chambers was collected in an "eggplant" flask, and the solvent was removed under reduced pressure. The reaction mixture was dissolved in EtOAc and washed with water (3 ×) and brine, respectively. The solution was dried over Na_2SO_4, and the solvent was removed under reduced pressure. The crude product was purified with preparative GPC to afford linear oligosaccharides **2a** (*n* = 2, 65 μmol, 63 mg), **3a** (*n* = 3, 34 μmol, 47 mg, **4a** (*n* = 4, 17 μmol, 31 mg), **5a** (*n* = 5, 9.4 μmol, 21 mg), **6a** (*n* = 6, 5.6 μmol, 15 mg), **7a** (*n* = 7, 4.2 μmol, 13 mg), and **8a** (*n* = 8, 2.3 μmol, 7.6 mg) as white solids.

4-Fluorophenyl (3-*O*-acetyl-6-*O*-benzyl-2-deoxy-2-phthalimido-β-D-glucopyranosyl)-(1→4)-(3-*O*-acetyl-6-*O*-benzyl-2-deoxy-2-phthalimido-β-D-glucopyranosyl)-(1→4)-(3-*O*-acetyl-6-*O*-benzyl-2-deoxy-2-phthalimido-β-D-glucopyranosyl)-(1→4)-(3-*O*-acetyl-6-*O*-benzyl-2-deoxy-2-phthalimido-β-D-glucopyranosyl)-(1→4)-(3-*O*-acetyl-6-*O*-benzyl-2-deoxy-2-phthalimido-β-D-glucopyranosyl)-(1→4)-(3-*O*-acetyl-6-*O*-benzyl-2-deoxy-2-phthalimido-β-D-glucopyranosyl)-(1→4)-(3-*O*-acetyl-6-*O*-benzyl-2-deoxy-2-phthalimido-β-D-glucopyranosyl)-(1→4)-3-*O*-acetyl-6-*O*-benzyl-2-deoxy-2-phthalimido-1-thio-β-D-glucopyranoside (**8a**); TLC (Hexane:EtOAc 1:2): R_f 0.13. $[\alpha]_D$ = –25.8 (*c* = 0.9, $CHCl_3$, 32 °C); ^{1}H NMR ($CDCl_3$, 600 MHz) δ 7.90–7.60 (m, 32 H), 7.36–7.25 (m, 10 H), 7.23–7.11 (m, 16 H), 7.08 (*pseudo*-t, *J* = 7.8 Hz, 2 H), 6.96–6.84 (m, 10 H), 6.81 (*pseudo*-t, *J* = 7.8 Hz, 2 H), 6.61 (*pseudo*-t, *J* = 7.2 Hz, 1 H), 6.52 (*pseudo*-t, *J* = 7.2 Hz, 1 H), 6.50–6.43 (m, 2 H), 5.56 (*pseudo*-t, *J* = 10.2 Hz, 1 H), 5.48 (dd, *J* = 10.2, 9.0 Hz, 1 H), 5.46–5.41 (m, 4 H), 5.38–5.30 (m, 5 H), 5.18 (d, *J* = 7.8 Hz, 1 H), 5.11 (d, *J* = 8.4 Hz, 1 H), 5.09 (d, *J* = 8.4 Hz, 1 H), 5.06–5.01 (m, 2 H), 4.51 (d, *J* = 11.4 Hz, 1 H), 4.47 (d, *J* = 11.4 Hz, 1 H), 4.44–4.27 (m, 14 H), 4.14–3.87 (m, 16 H), 3.76 (*pseudo*-t, *J* = 9.0 Hz, 1 H), 3.70 (dd, *J* = 9.6, 3.6 Hz, 1 H), 3.62 (dd, *J* = 9.6, 4.2 Hz, 1 H), 3.52 (d, *J* = 10.2 Hz, 1 H), 3.45–3.35 (m, 8 H), 3.24–3.16 (m, 4 H), 3.14–3.06 (m, 4 H), 2.97 (d, *J* = 9.6 Hz, 1 H), 2.70 (d, *J* = 10.2 Hz, 1 H), 2.63 (d, *J* = 9.6 Hz, 1 H), 2.60–2.54 (m, 1 H), 1.86 (s, 3 H), 1.77 (s, 3 H), 1.73 (s, 3 H), 1.703 (s, 3 H), 1.698 (s, 3 H), 1.695 (s, 3 H), 1.68 (s, 3 H), 1.64 (s, 3 H); ^{13}C NMR ($CDCl_3$, 150 MHz) δ 170.9, 170.4, 170.3, 170.2, 170.1, 168.0, 167.9, 167.2, 167.1, 163.0 (d, *J* = 247.4 Hz), 138.2, 138.12, 138.09, 138.07, 137.4, 135.9 (d, *J* = 8.3 Hz), 134.3, 134.23, 134.20, 134.1, 131.6, 131.55, 131.48, 131.40, 128.81, 128.78, 128.5, 128.2, 128.0, 127.89, 127.86, 127.84, 127.82, 127.78, 127.6, 127.35, 127.19, 127.17, 127.14, 127.05, 126.88, 126.82, 126.77, 126.76, 125.9, 123.66, 123.65, 123.64, 123.58, 123.54, 123.49, 123.48, 123.44, 123.36 115.8 (d, *J* = 21.9 Hz), 96.5, 96.4, 95.9, 95.7, 82.66, 82.65, 78.4, 73.8, 73.6, 73.5, 73.25, 73.20, 73.0, 72.8, 72.5, 72.2, 71.94, 71.91, 71.7, 71.25, 71.19, 70.7, 70.6, 69.8, 67.7, 67.45, 67.41, 66.2, 65.9, 55.25, 55.12, 55.07, 54.8, 20.56, 20.52, 20.4; HRMS (ESI) *m/z* calculated for $C_{190}H_{173}FKN_8O_{56}S$ $[M+K]^+$, 3552.0272; found, 3552.0203.

8. References

1 T. Nokami, Y. Isoda, N. Sasaki, A. Takaiso, S. Hayase, T. Itoh, R. Hayashi, A. Shimizu, and J. Yoshida, *Org. Lett.*, **2015**, *17*, 1525-1528.

2 K. Yano, T. Itoh and T. Nokami, *Carbohydr. Res.*, **2020**, *492*, 108018.

9. ^{1}H and ^{13}C NMR, H,H-COSY, and HMQC spectra of oligosaccharides

^{1}H NMR

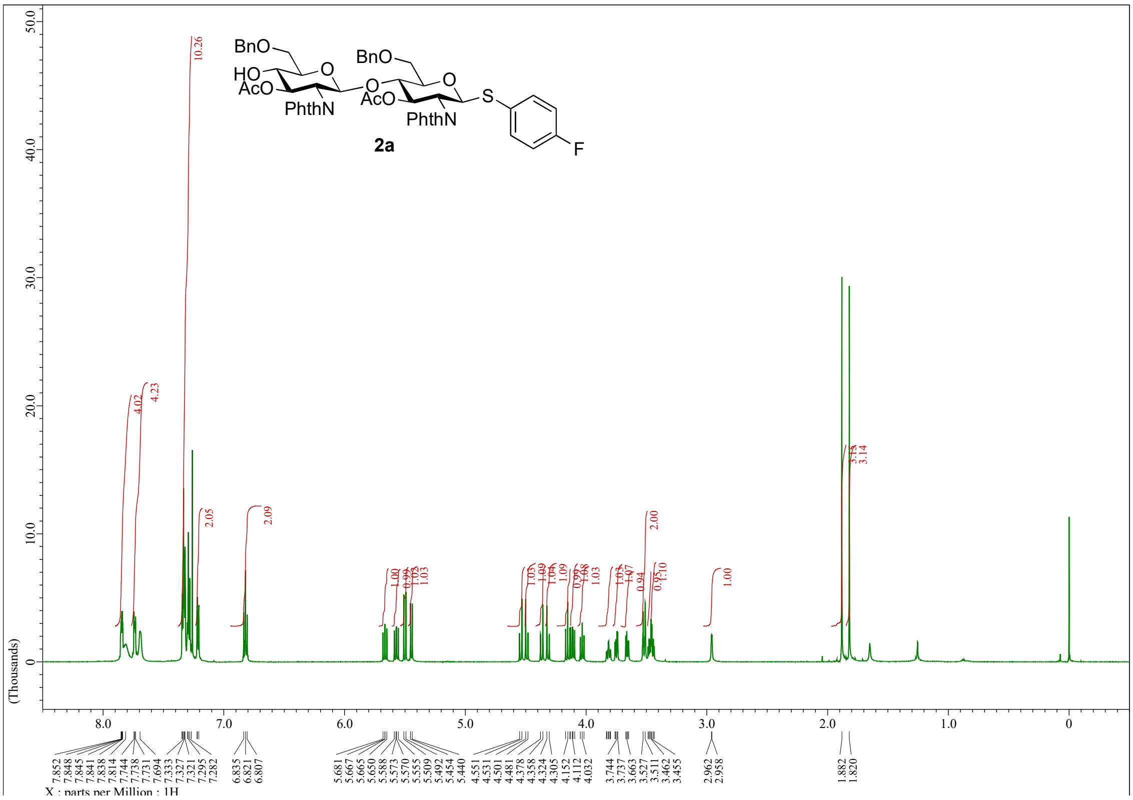

^{13}C NMR

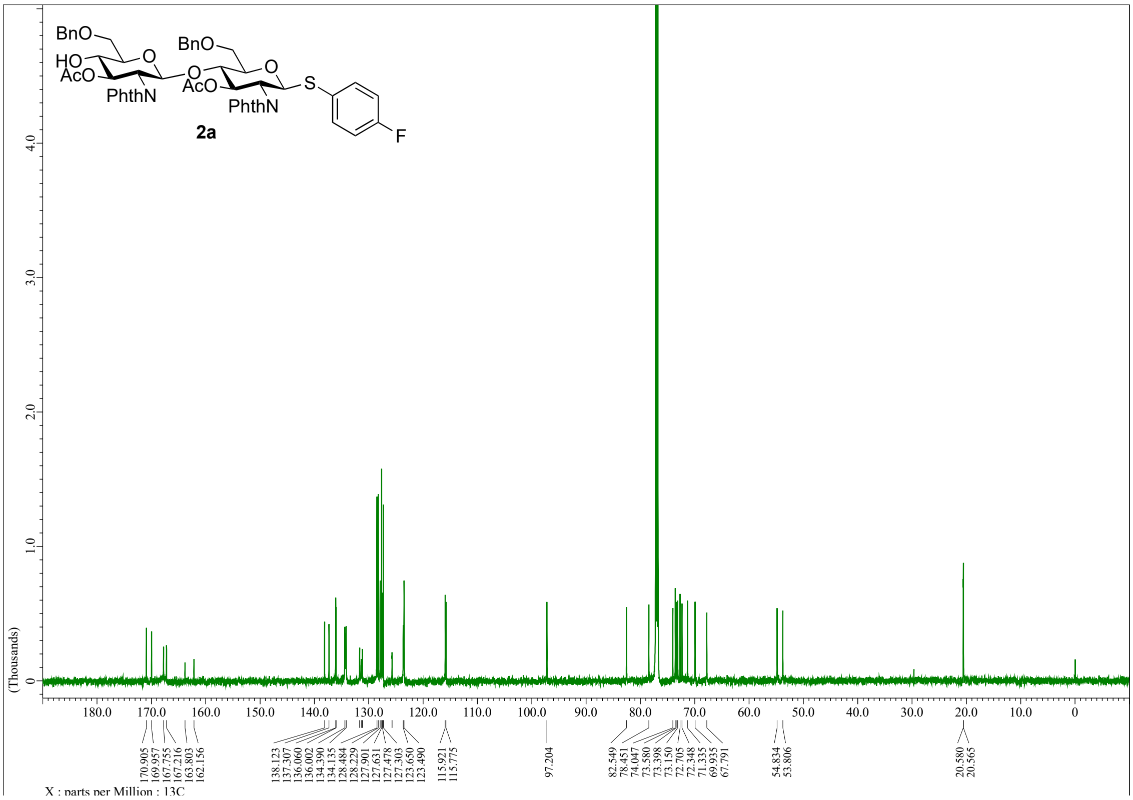

H,H-COSY

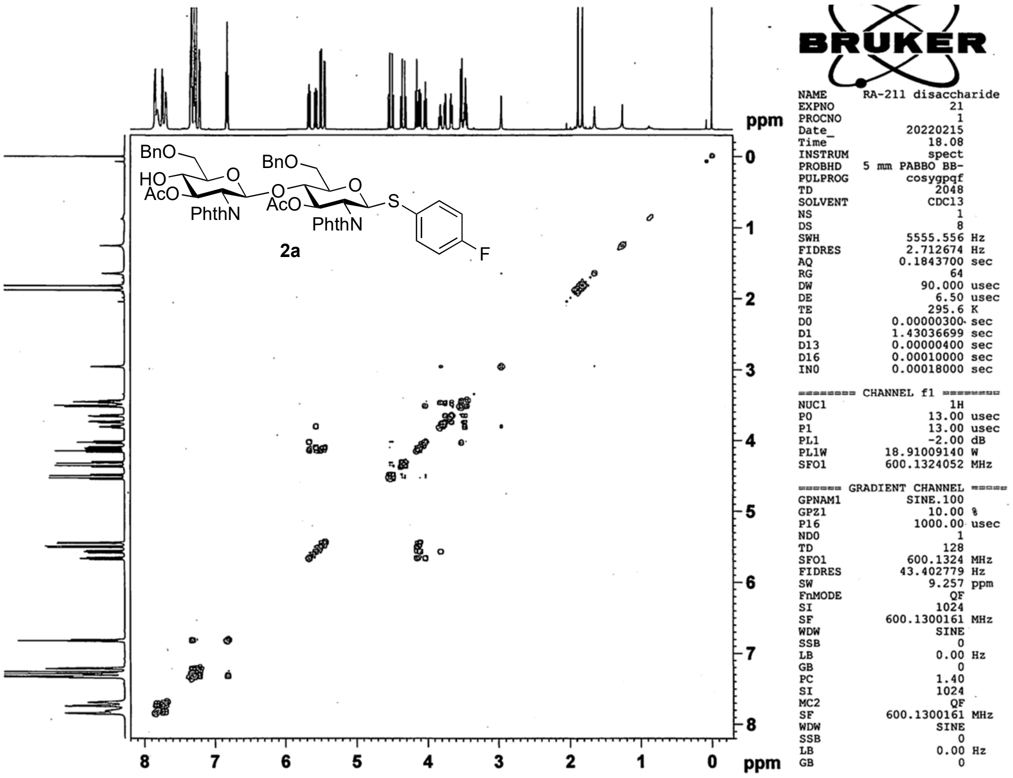
BnO
HO
AcO
PhthN
O
BnO
AcO
PhthN
S
F
2a
ppm
NAME RA-211 disaccharide
EXPNO 21
PROCNO 1
Date_ 20220215
Time 18.08
INSTRUM spect
PROBHD 5 mm PABBO BB-
PULPROG cosygpqf
TD 2048
SOLVENT CDCl3
NS 1
DS 8
SWH 5555.556 Hz
FIDRES 2.712674 Hz
AQ 0.1843700 sec
RG 64
DW 90.000 usec
DE 6.50 usec
TE 295.6 K
D0 0.00000300 sec
D1 1.43036699 sec
D13 0.00000400 sec
D16 0.00010000 sec
IN0 0.00018000 sec
======== CHANNEL f1 ========
NUC1 1H
P0 13.00 usec
P1 13.00 usec
PL1 -2.00 dB
PL1W 18.91009140 W
SFO1 600.1324052 MHz
====== GRADIENT CHANNEL =====
GPNAM1 SINE.100
GPZ1 10.00 %
P16 1000.00 usec
ND0 1
TD 128
SFO1 600.1324 MHz
FIDRES 43.402779 Hz
SW 9.257 ppm
FnMODE QF
SI 1024
SF 600.1300161 MHz
WDW SINE
SSB 0
LB 0.00 Hz
GB 0
PC 1.40
SI 1024
MC2 QF
SF 600.1300161 MHz
WDW SINE
SSB 0
LB 0.00 Hz
GB 0

HMQC

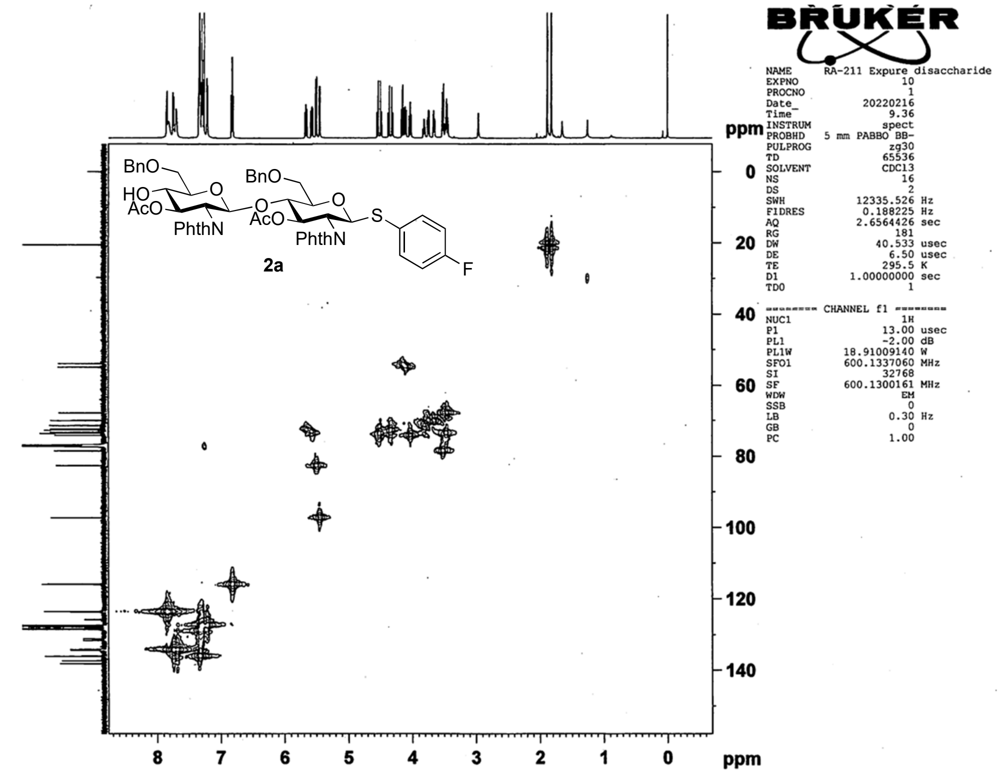
BnO
HO
AcO
PhthN
O
BnO
AcO
PhthN
S
F
2a
ppm
NAME RA-211 Expure disaccharide
EXPNO 10
PROCNO 1
Date_ 20220216
Time 9.36
INSTRUM spect
PROBHD 5 mm PABBO BB-
PULPROG zg30
TD 65536
SOLVENT CDCl3
NS 16
DS 2
SWH 12335.526 Hz
FIDRES 0.188225 Hz
AQ 2.6564426 sec
RG 181
DW 40.533 usec
DE 6.50 usec
TE 295.5 K
D1 1.00000000 sec
TD0 1
======== CHANNEL f1 ========
NUC1 1H
P1 13.00 usec
PL1 -2.00 dB
PL1W 18.91009140 W
SFO1 600.1337060 MHz
SI 32768
SF 600.1300161 MHz
WDW EM
SSB 0
LB 0.30 Hz
GB 0
PC 1.00

^{1}H NMR

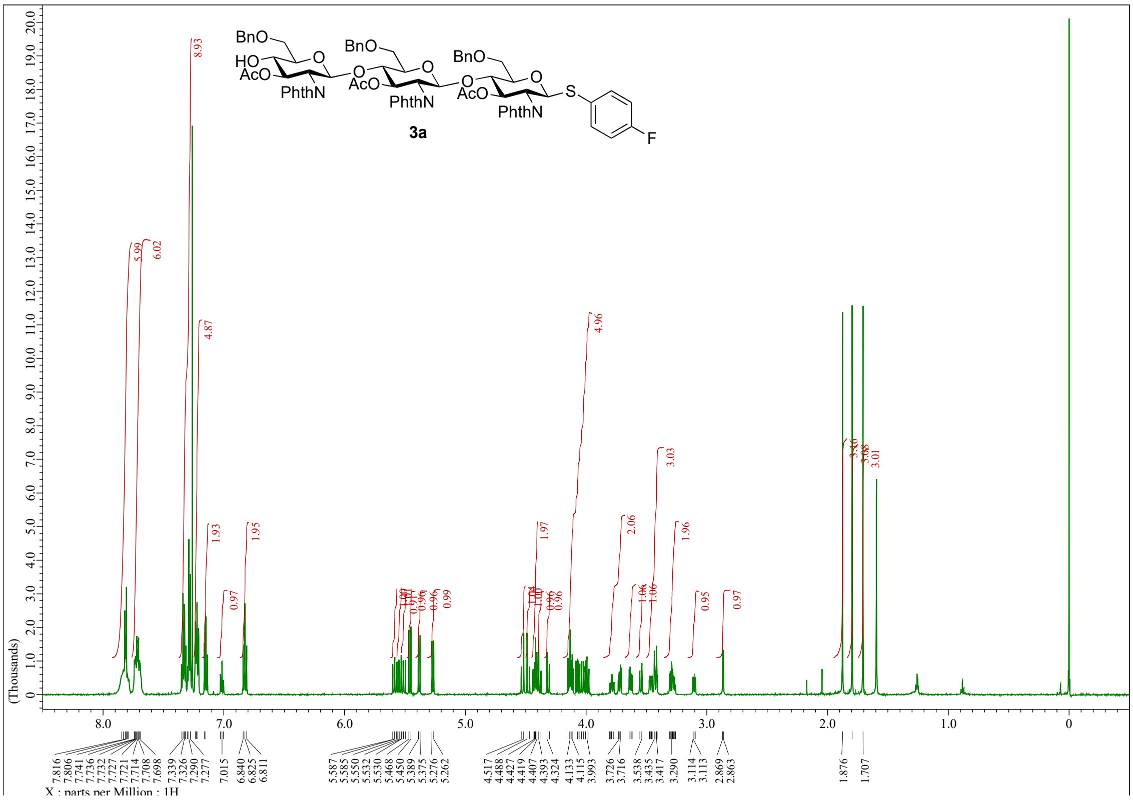

^{13}C NMR

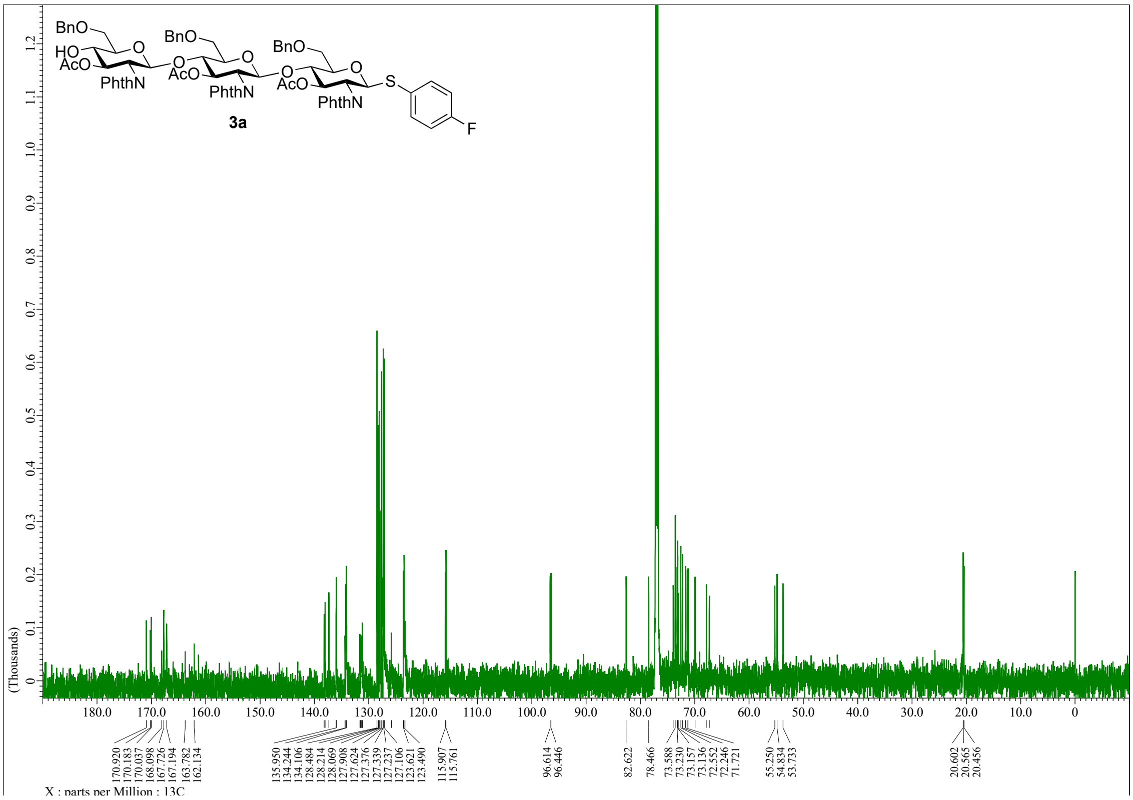

H,H-COSY

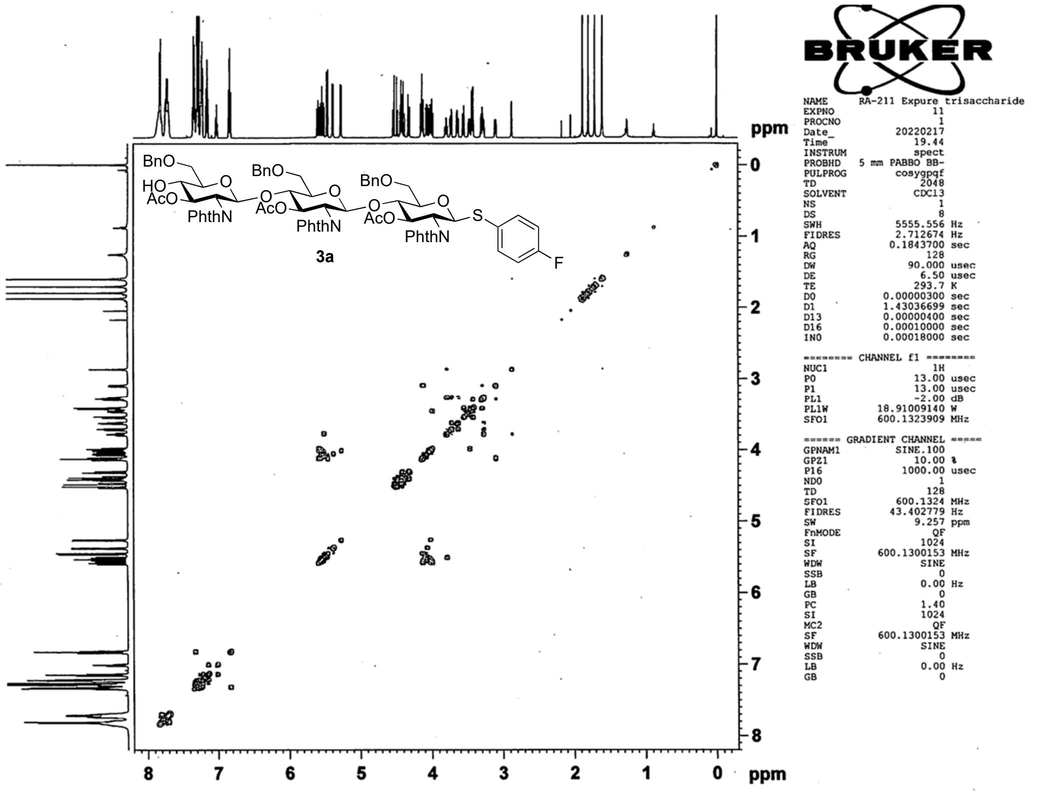
BnO
HO
AcO
PhthN
BnO
AcO
PhthN
BnO
AcO
PhthN
S
F
3a
ppm
ppm
BRUKER
NAME RA-211 Expure trisaccharide

HMQC

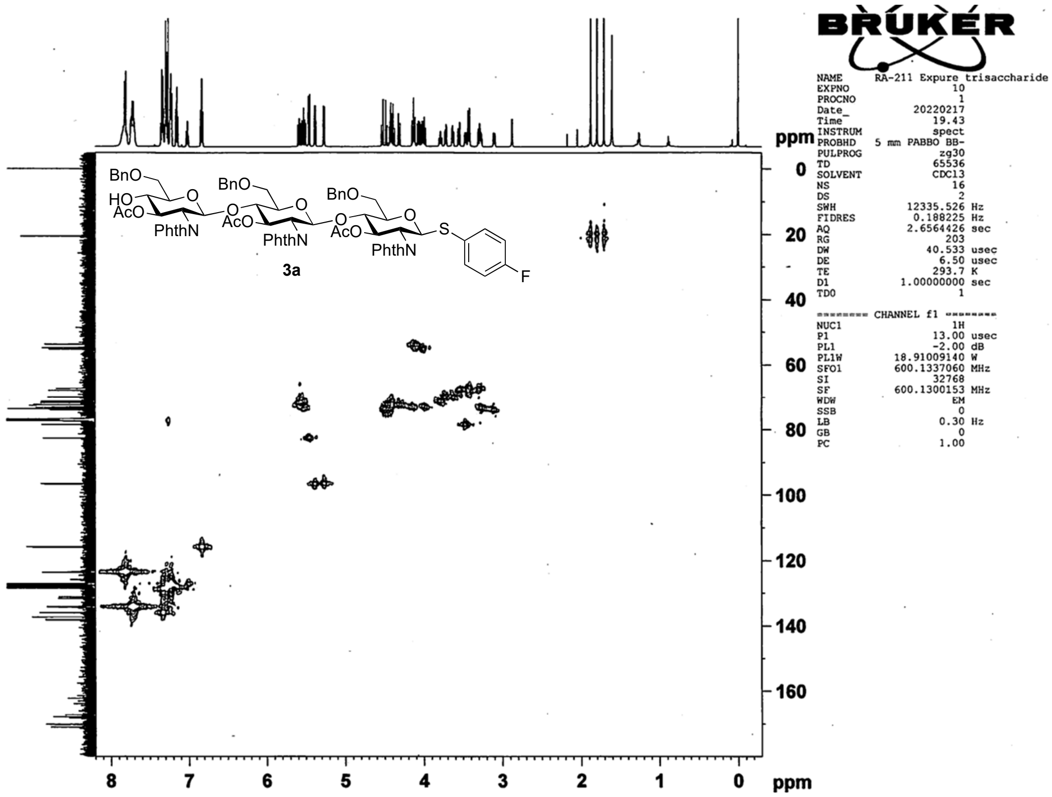
BnO
HO
AcO
PhthN
BnO
AcO
PhthN
BnO
AcO
PhthN
S
F
3a
ppm
ppm
BRUKER
NAME RA-211 Expure trisaccharide

^{1}H NMR

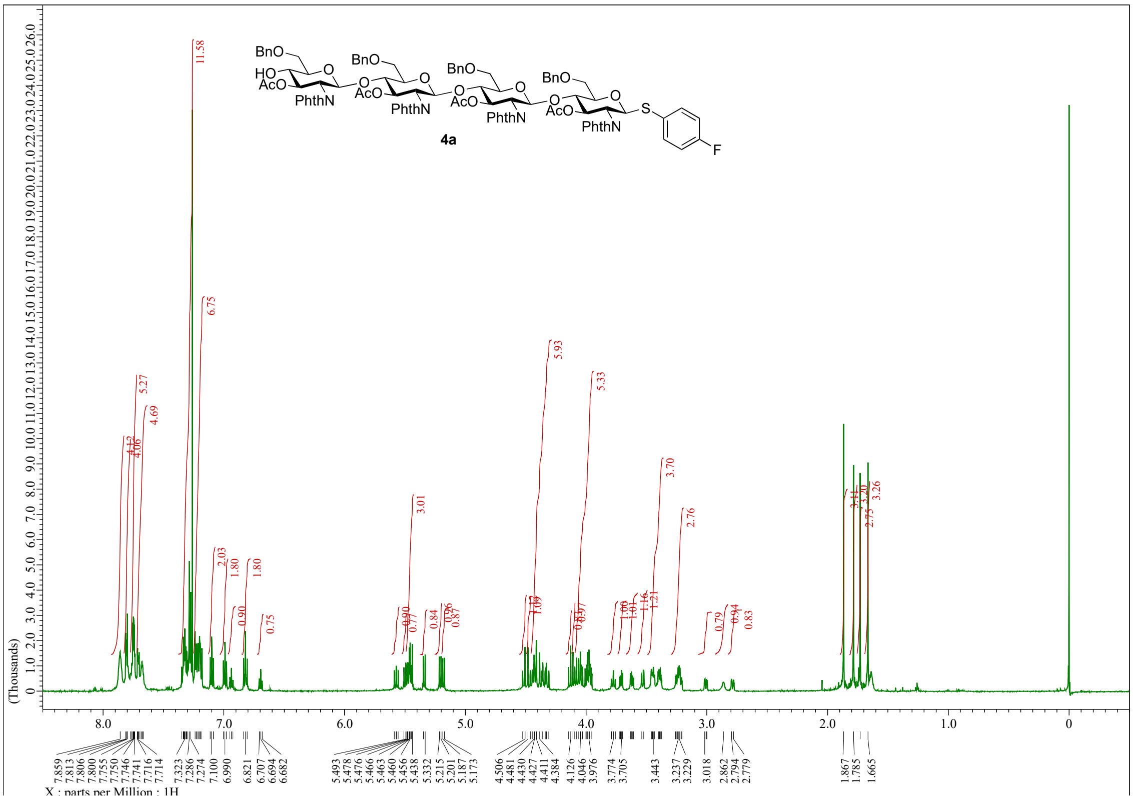

^{13}C NMR

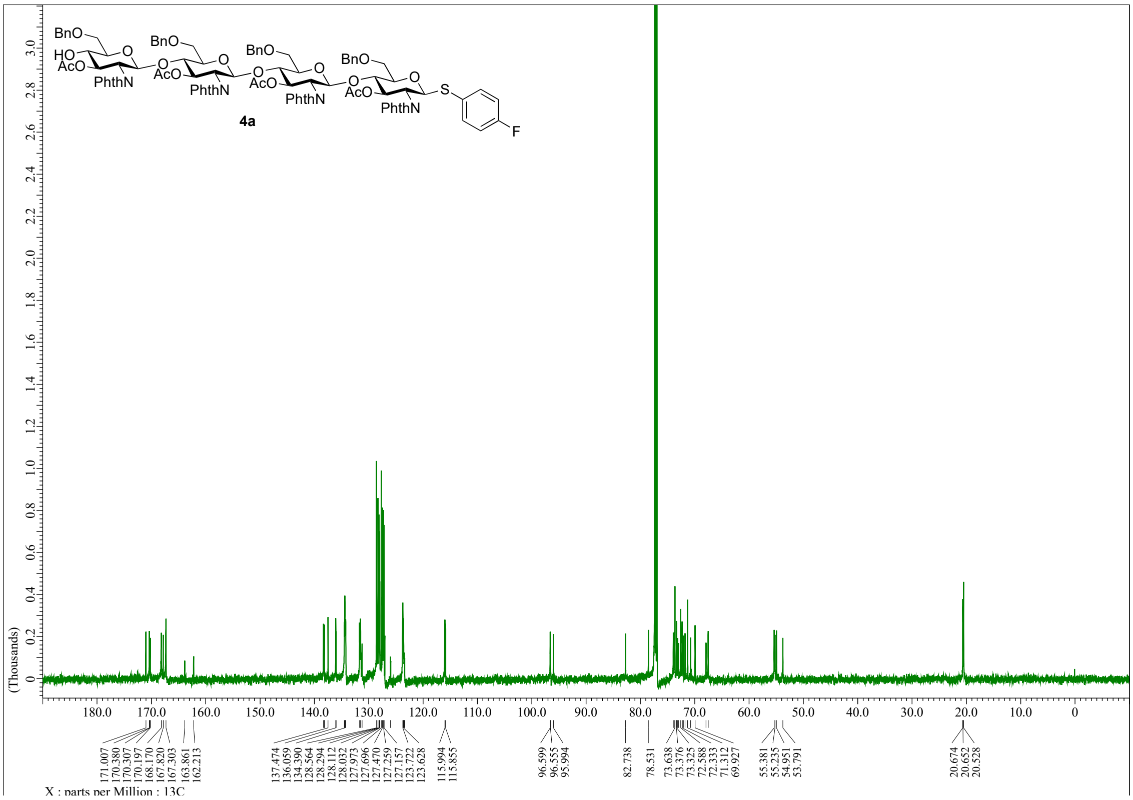

H,H-COSY

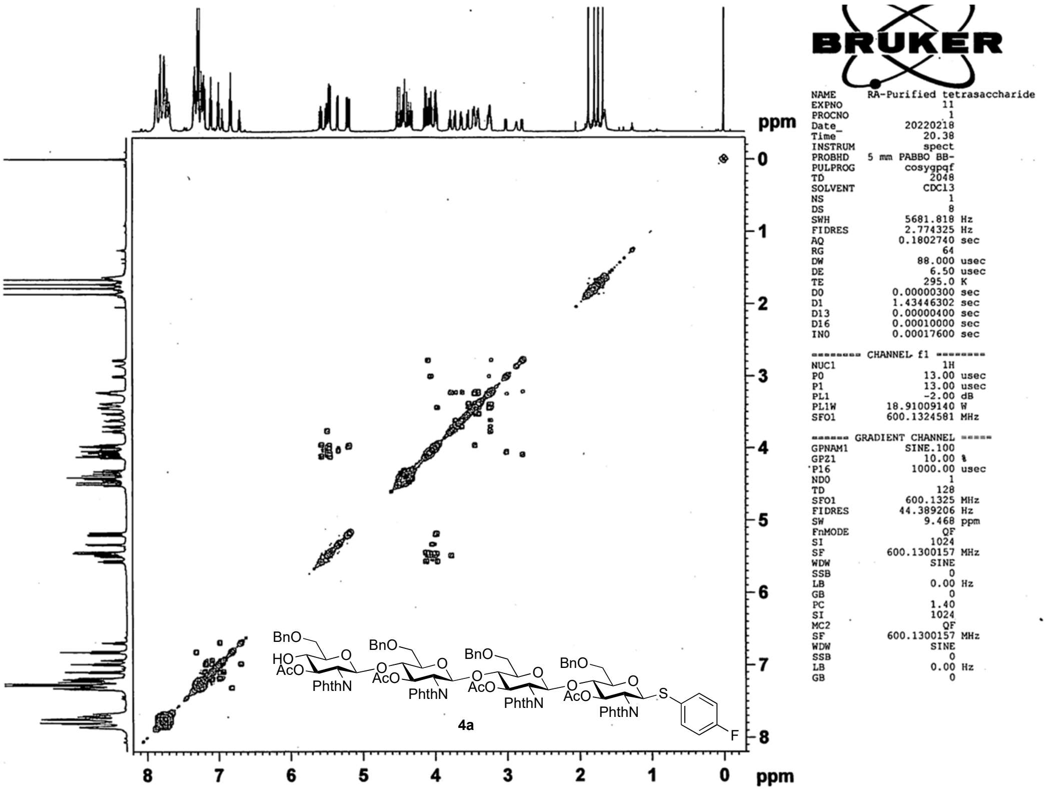
BRUKER
NAME RA-Purified tetrasaccharide
EXPNO 11
PROCNO 1
Date_ 20220218
Time 20.38
INSTRUM spect
PROBHD 5 mm PABBO BB-
PULPROG cosygpqf
TD 2048
SOLVENT CDCl3
NS 1
DS 8
SWH 5681.818 Hz
FIDRES 2.774325 Hz
AQ 0.1802740 sec
RG 64
DW 88.000 usec
DE 6.50 usec
TE 295.0 K
D0 0.00000300 sec
D1 1.43446302 sec
D13 0.00000400 sec
D16 0.00010000 sec
IN0 0.00017600 sec
======== CHANNEL f1 ========
NUC1 1H
P0 13.00 usec
P1 13.00 usec
PL1 -2.00 dB
PL1W 18.91009140 W
SFO1 600.1324581 MHz
====== GRADIENT CHANNEL =====
GPNAM1 SINE.100
GPZ1 10.00 %
P16 1000.00 usec
ND0 1
TD 128
SFO1 600.1325 MHz
FIDRES 44.389206 Hz
SW 9.468 ppm
FnMODE QF
SI 1024
SF 600.1300157 MHz
WDW SINE
SSB 0
LB 0.00 Hz
GB 0
PC 1.40
SI 1024
MC2 QF
SF 600.1300157 MHz
WDW SINE
SSB 0
LB 0.00 Hz
GB 0
BnO
HO
AcO
PhthN
AcO
PhthN
AcO
PhthN
AcO
PhthN
S
F
4a
ppm

HMQC

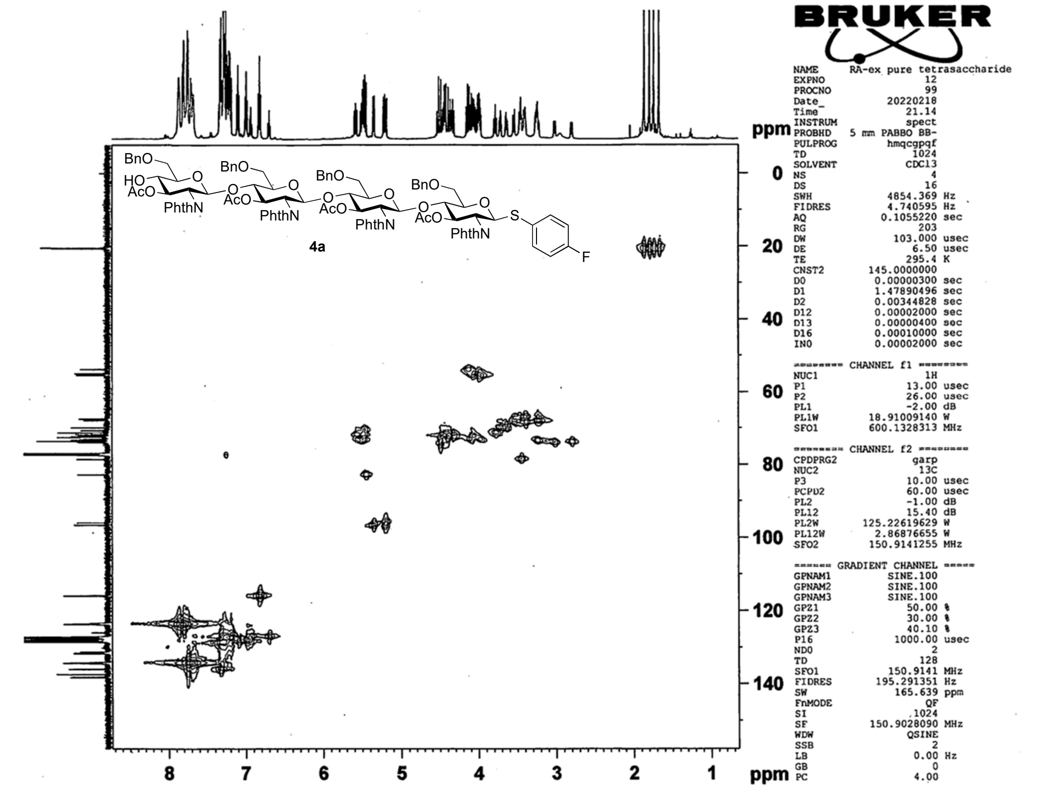
BRUKER
NAME RA-ex pure tetrasaccharide
EXPNO 12
PROCNO 99
Date_ 20220218
Time 21.14
INSTRUM spect
PROBHD 5 mm PABBO BB-
PULPROG hmqcgpqf
TD 1024
SOLVENT CDCl3
NS 4
DS 16
SWH 4854.369 Hz
FIDRES 4.740595 Hz
AQ 0.1055220 sec
RG 203
DW 103.000 usec
DE 6.50 usec
TE 295.4 K
CNST2 145.0000000
D0 0.00000300 sec
D1 1.47890496 sec
D2 0.00344828 sec
D12 0.00002000 sec
D13 0.00000400 sec
D16 0.00010000 sec
IN0 0.00002000 sec
======== CHANNEL f1 ========
NUC1 1H
P1 13.00 usec
P2 26.00 usec
PL1 -2.00 dB
PL1W 18.91009140 W
SFO1 600.1328313 MHz
======== CHANNEL f2 ========
CPDPRG2 garp
NUC2 13C
P3 10.00 usec
PCPD2 60.00 usec
PL2 -1.00 dB
PL12 15.40 dB
PL2W 125.22619629 W
PL12W 2.86876655 W
SFO2 150.9141255 MHz
====== GRADIENT CHANNEL =====
GPNAM1 SINE.100
GPNAM2 SINE.100
GPNAM3 SINE.100
GPZ1 50.00 %
GPZ2 30.00 %
GPZ3 40.10 %
P16 1000.00 usec
ND0 2
TD 128
SFO1 150.9141 MHz
FIDRES 195.291351 Hz
SW 165.639 ppm
FnMODE QF
SI 1024
SF 150.9028090 MHz
WDW QSINE
SSB 2
LB 0.00 Hz
GB 0
PC 4.00
BnO
HO
AcO
PhthN
AcO
PhthN
AcO
PhthN
AcO
PhthN
S
F
4a
ppm

^{1}H NMR

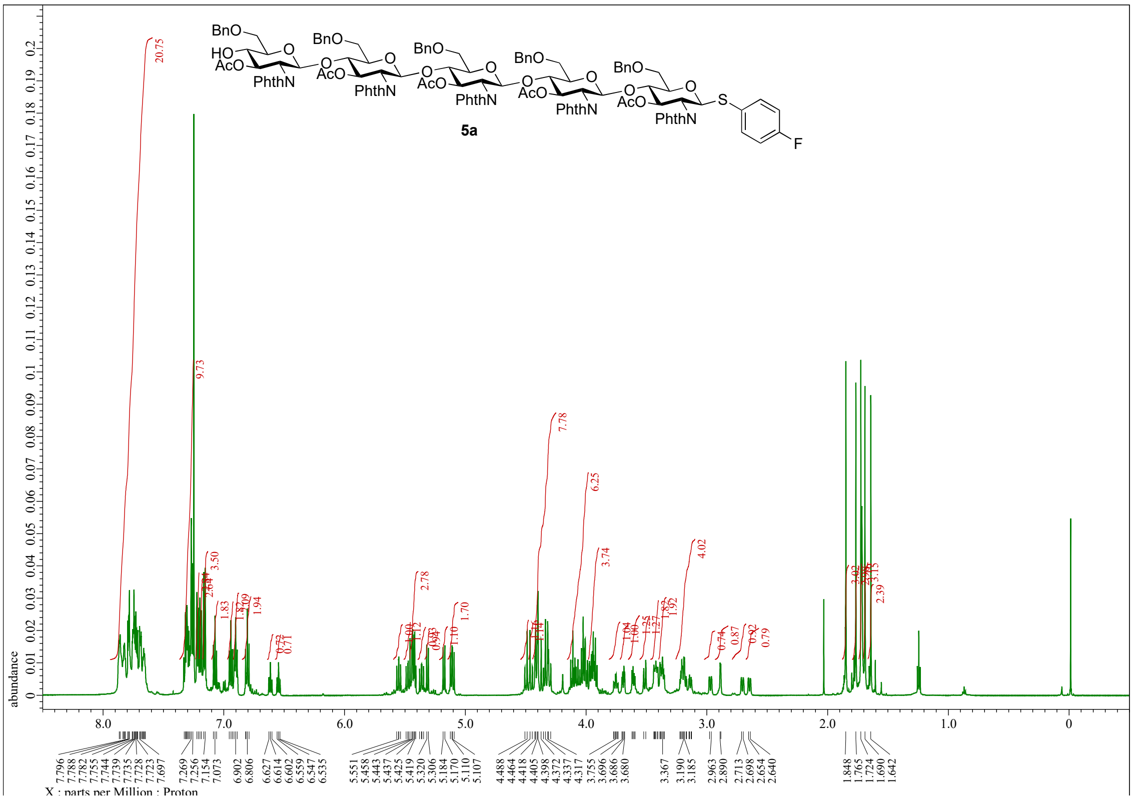

^{13}C NMR

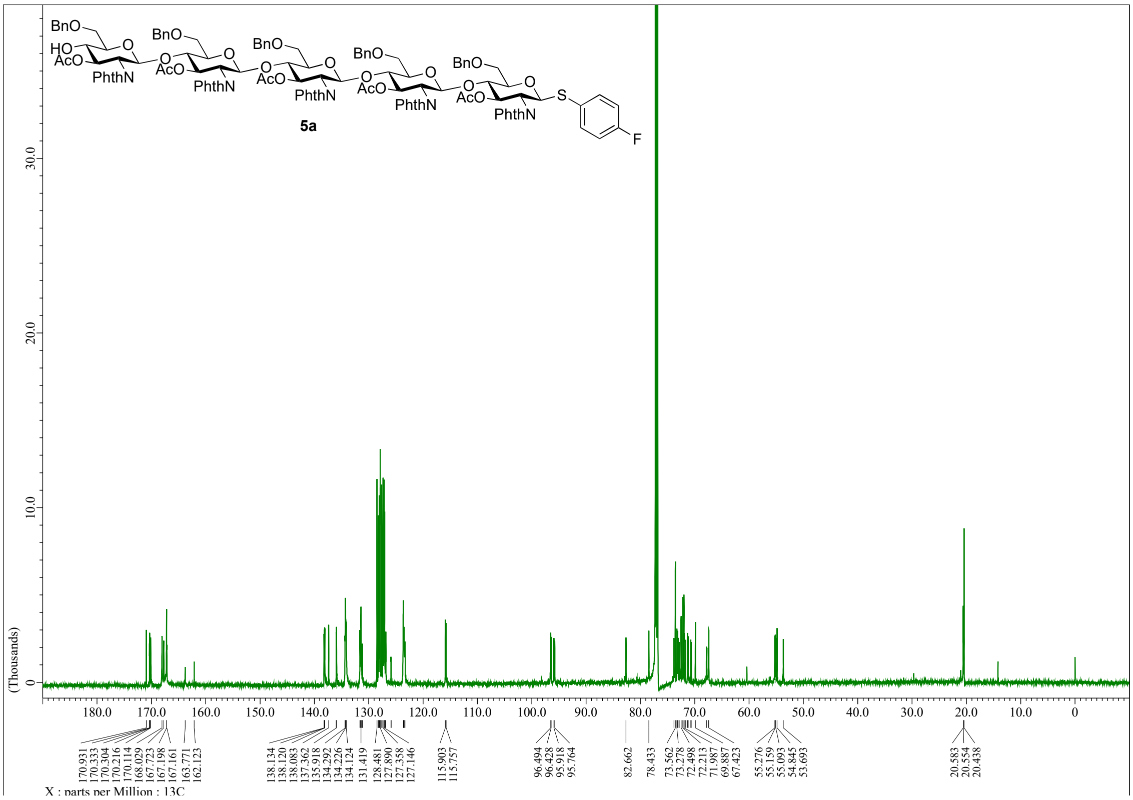

H,H-COSY

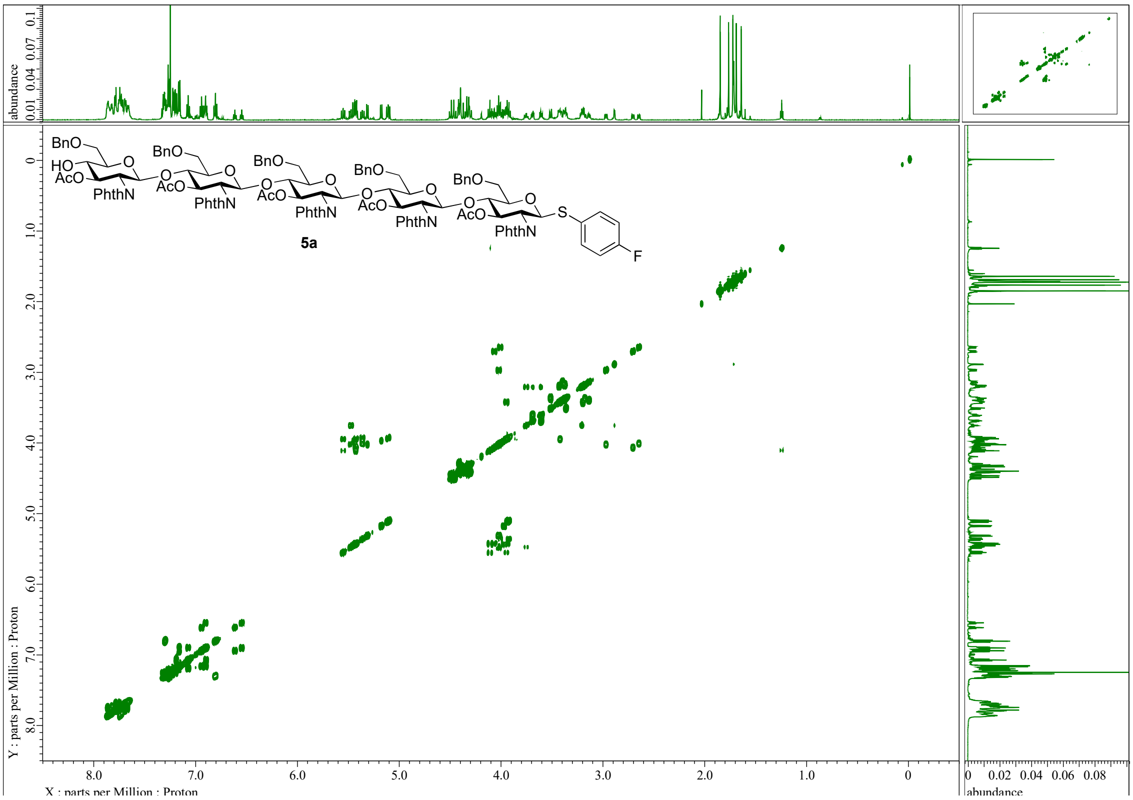
abundance
Y : parts per Million : Proton
X : parts per Million : Proton
BnO
HO
AcO
PhthN
5a
S
F
abundance

HMQC

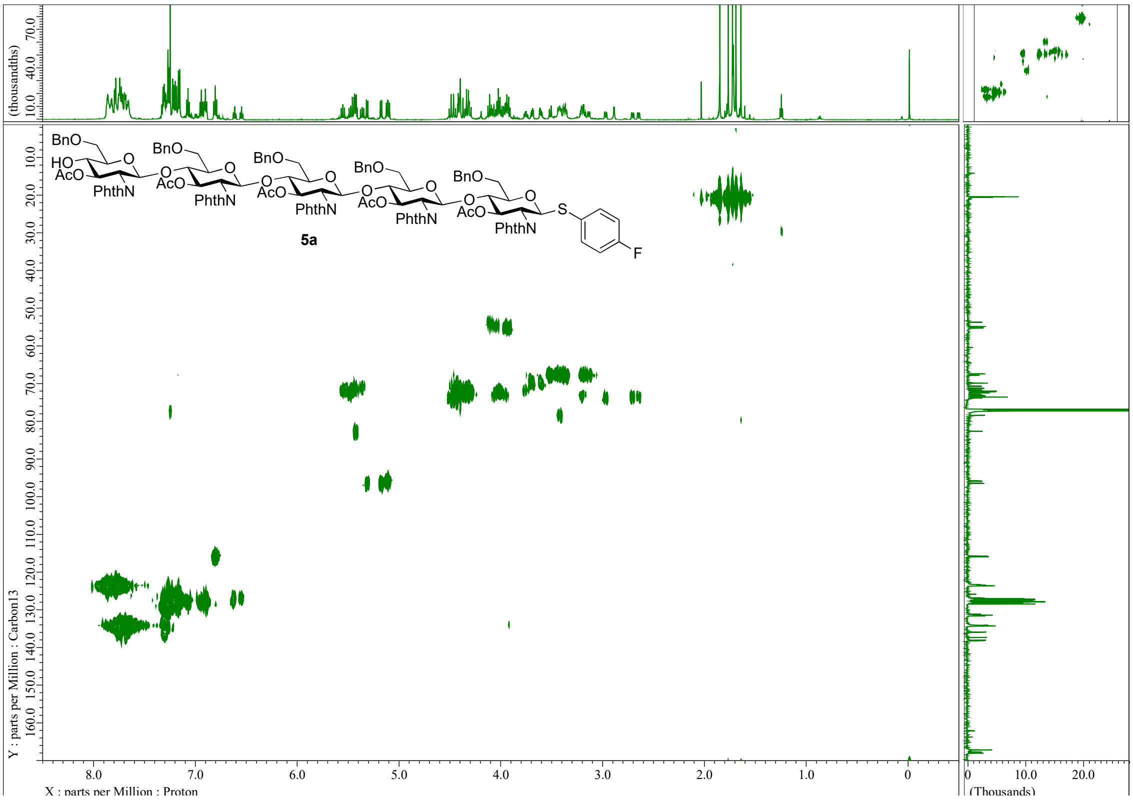
(thousandths)
Y : parts per Million : Carbon13
X : parts per Million : Proton
BnO
HO
AcO
PhthN
5a
S
F
(Thousands)

^{1}H NMR

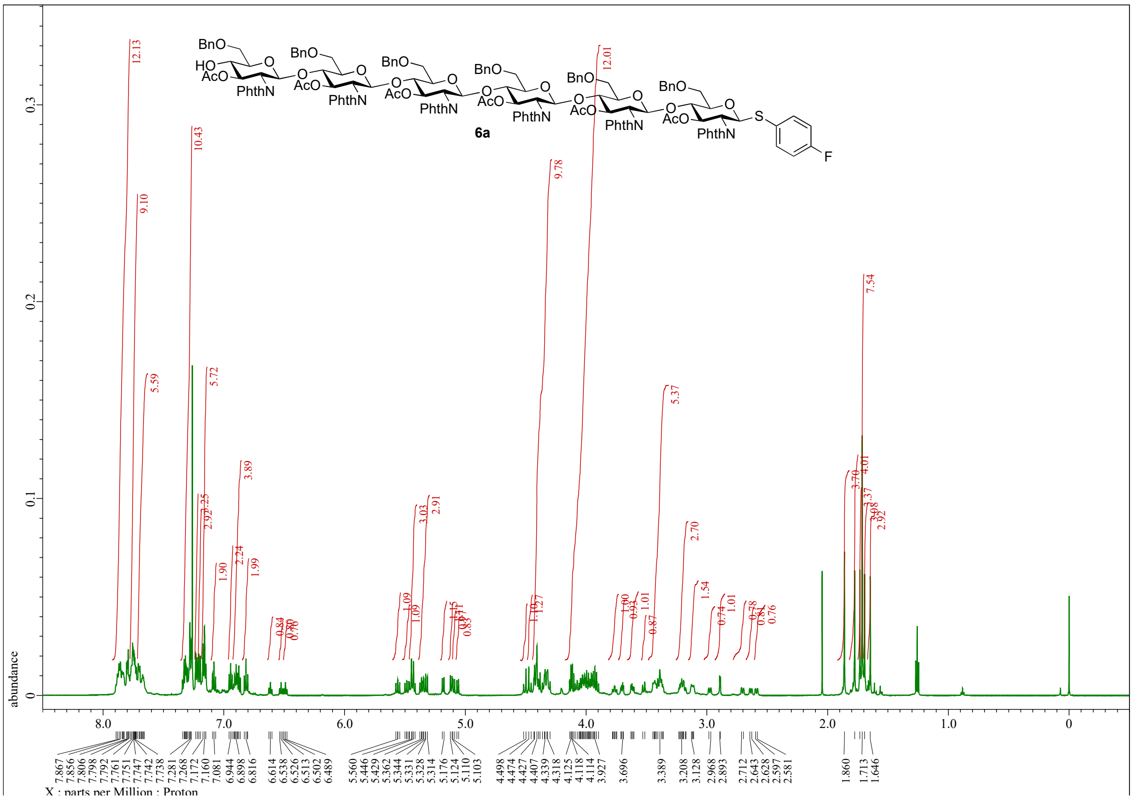

^{13}C NMR

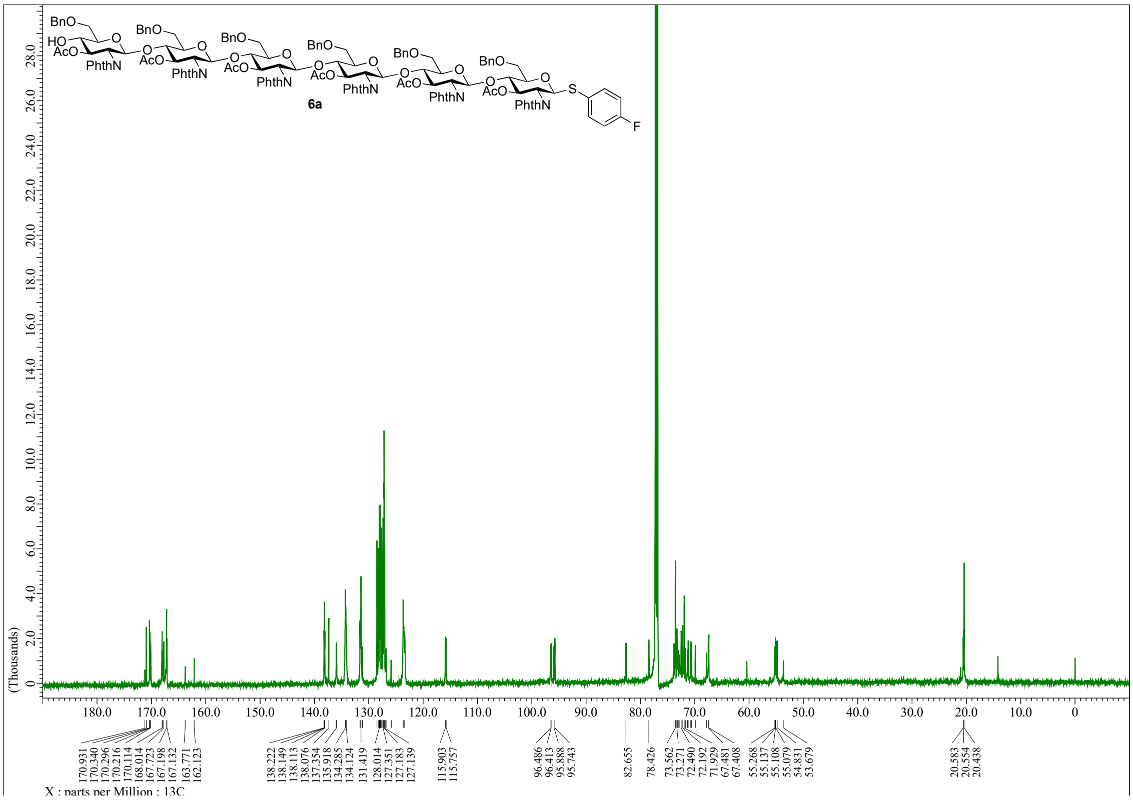

H,H-COSY

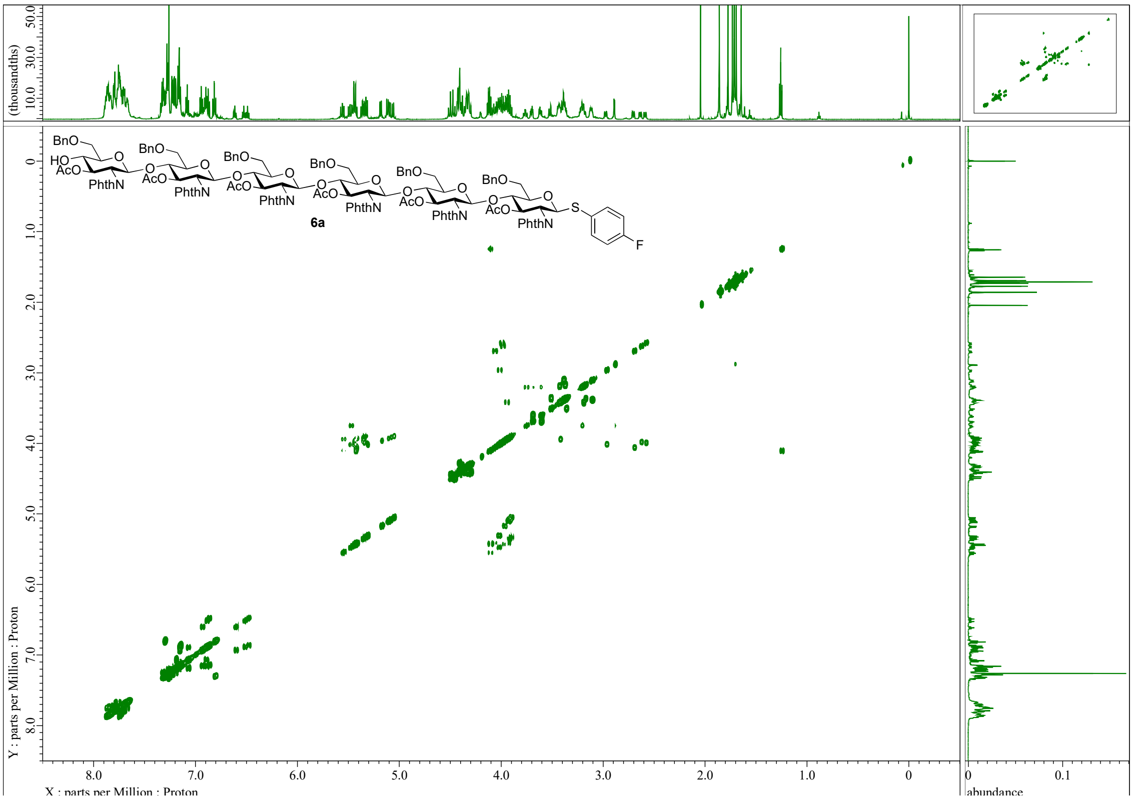
(thousandths)
50.0
30.0
10.0
Y : parts per Million : Proton
X : parts per Million : Proton
abundance
6a
BnO
HO
AcO
PhthN
S
F
8.0
7.0
6.0
5.0
4.0
3.0
2.0
1.0
0
0.1

HMQC

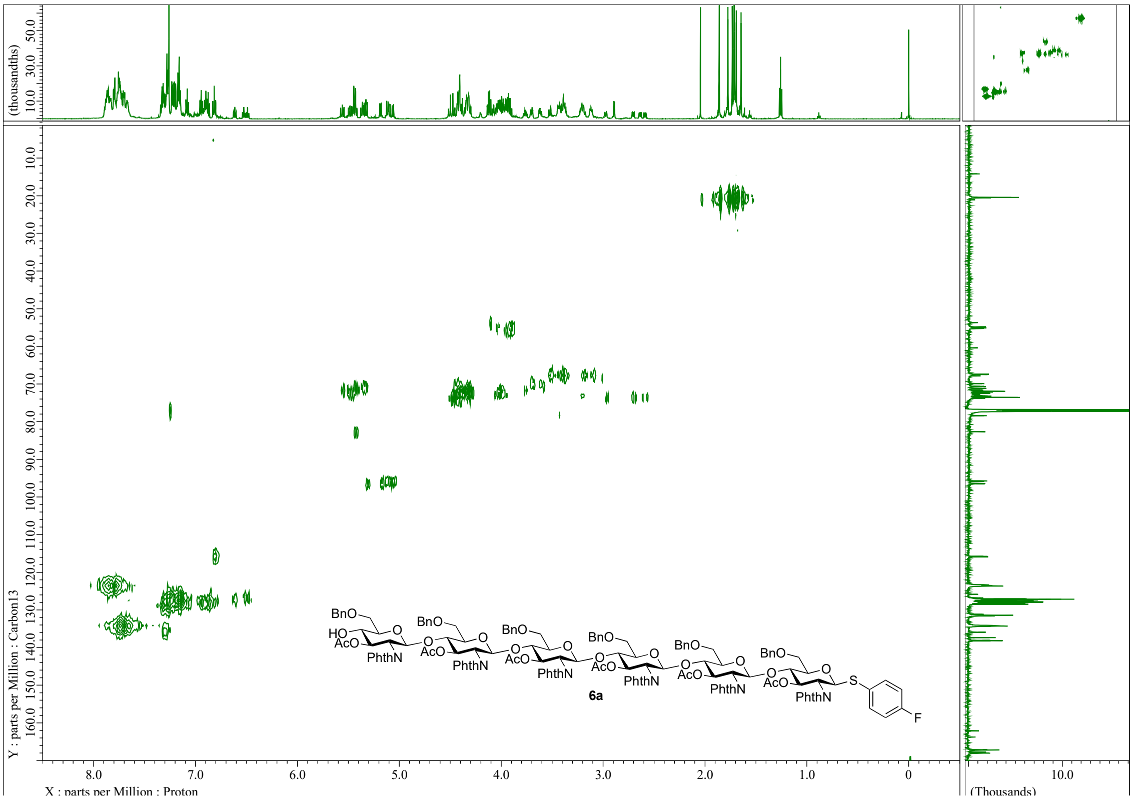
(thousandths)
Y : parts per Million : Carbon13
X : parts per Million : Proton
(Thousands)
6a
BnO
HO
AcO
PhthN
S
F
10.0

^{1}H NMR

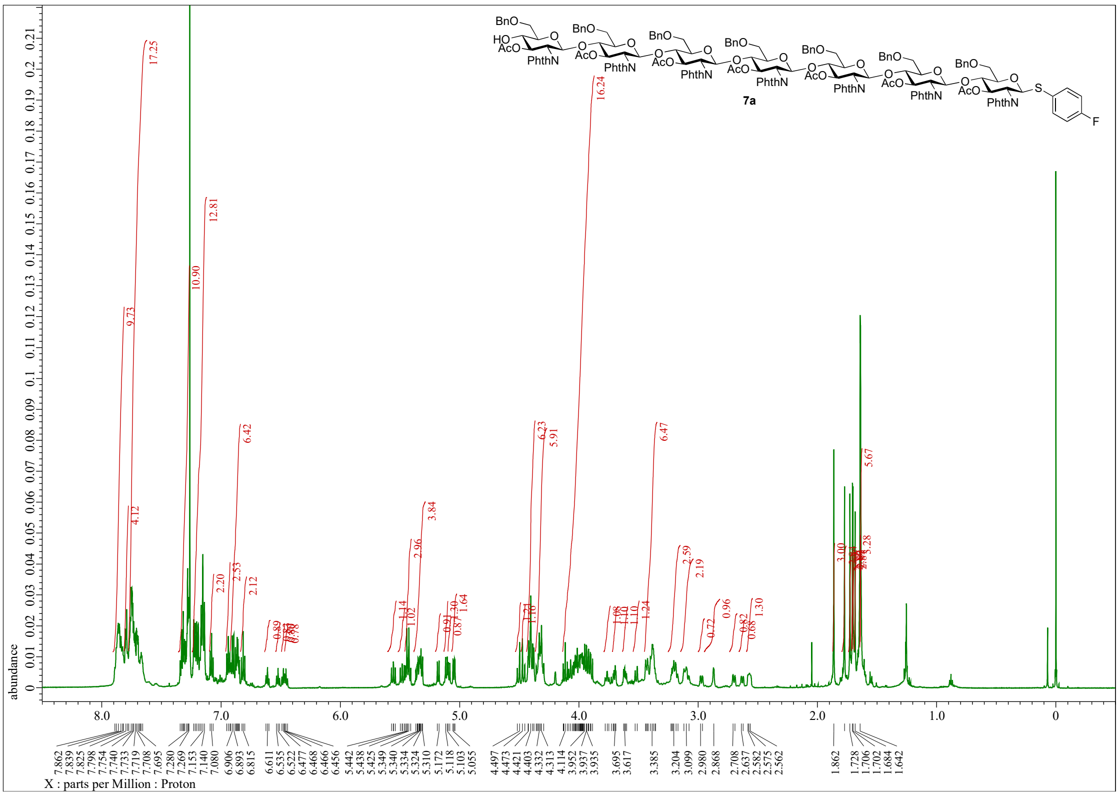

^{13}C NMR

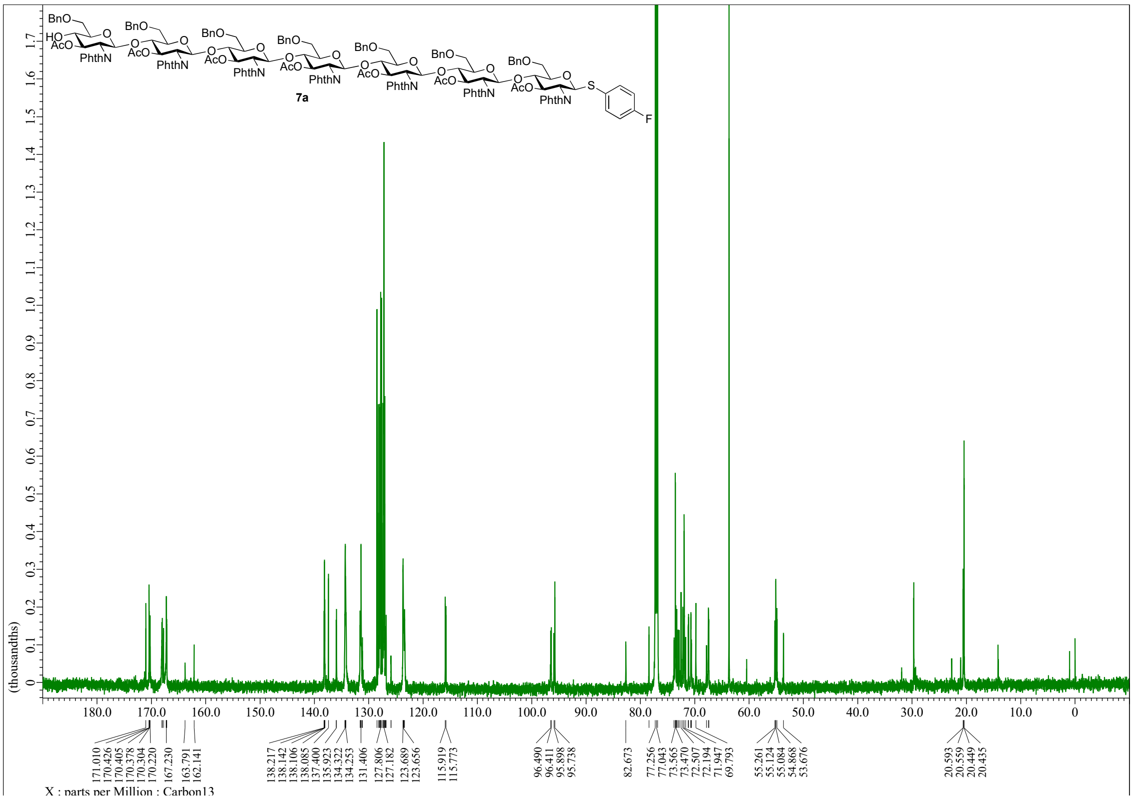

H,H-COSY

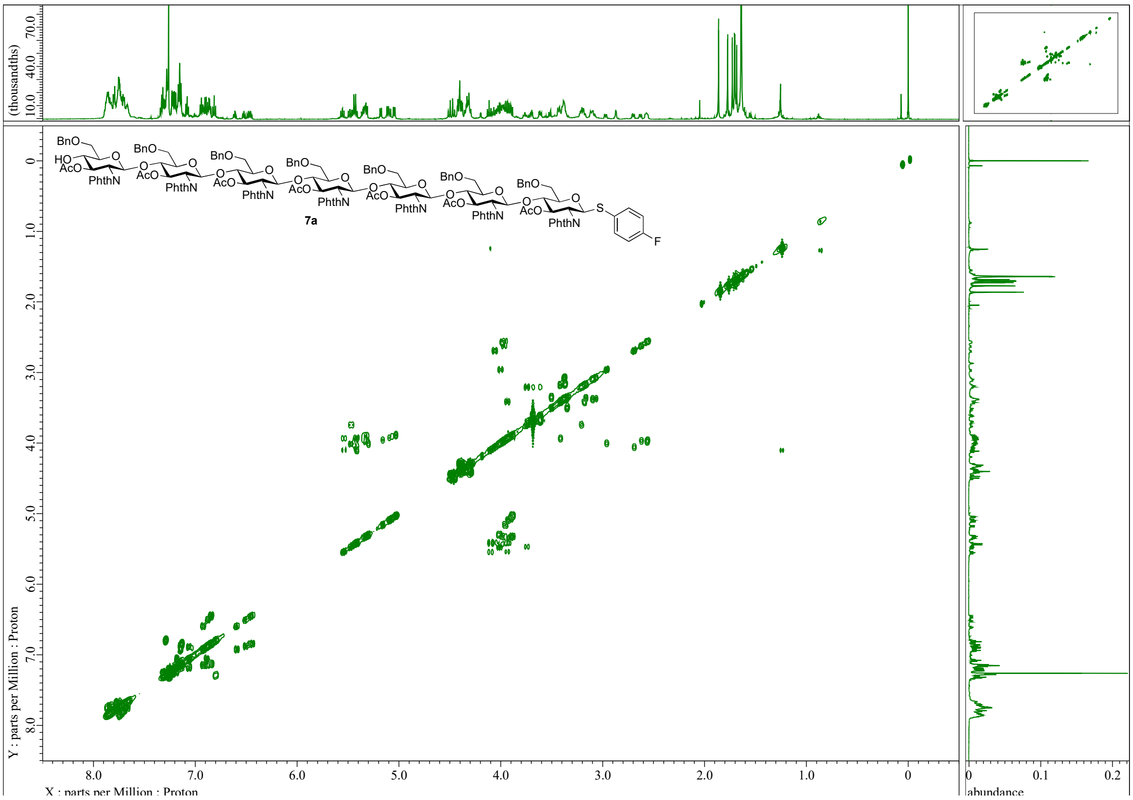

7a
X : parts per Million : Proton
Y : parts per Million : Proton
abundance

HMQC

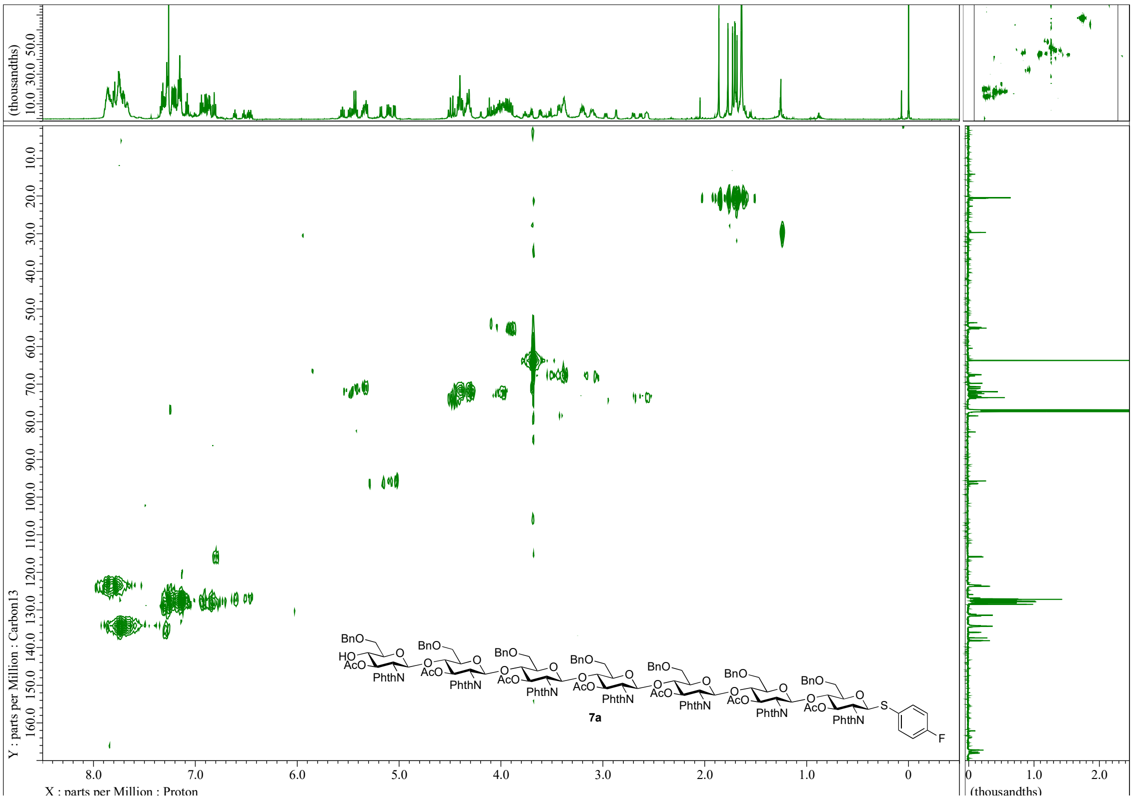

7a
X : parts per Million : Proton
Y : parts per Million : Carbon13

^{1}H NMR

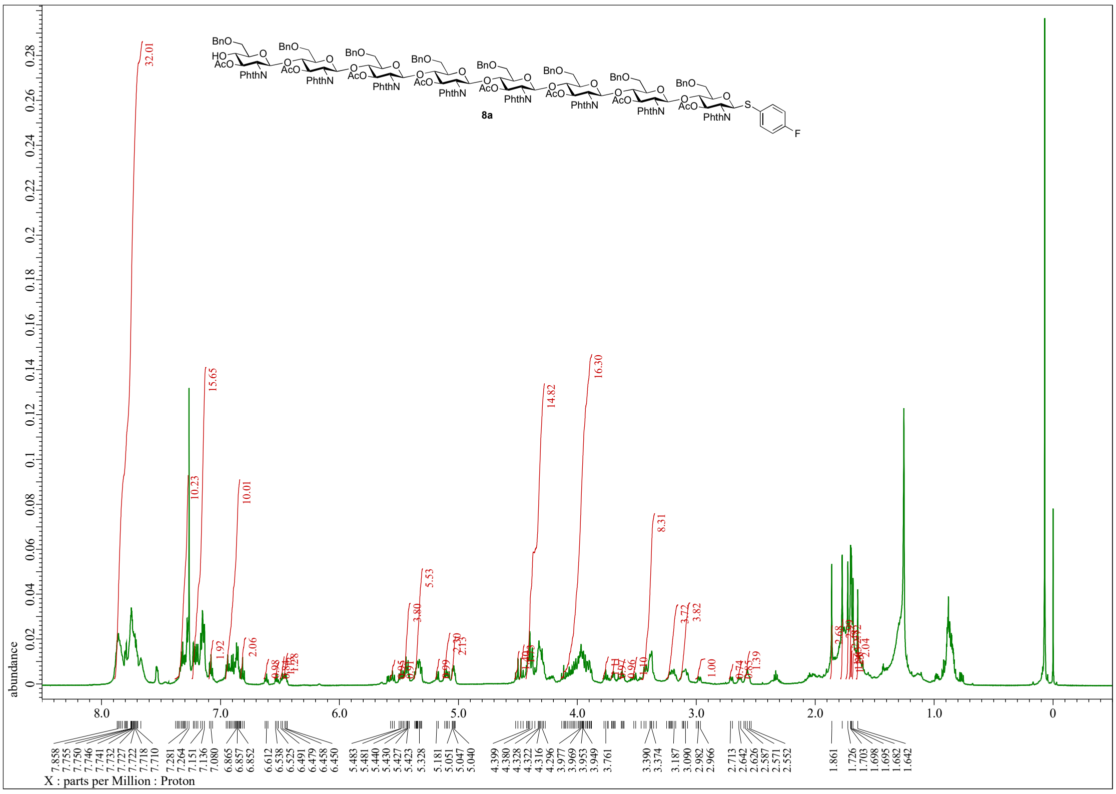

^{13}C NMR

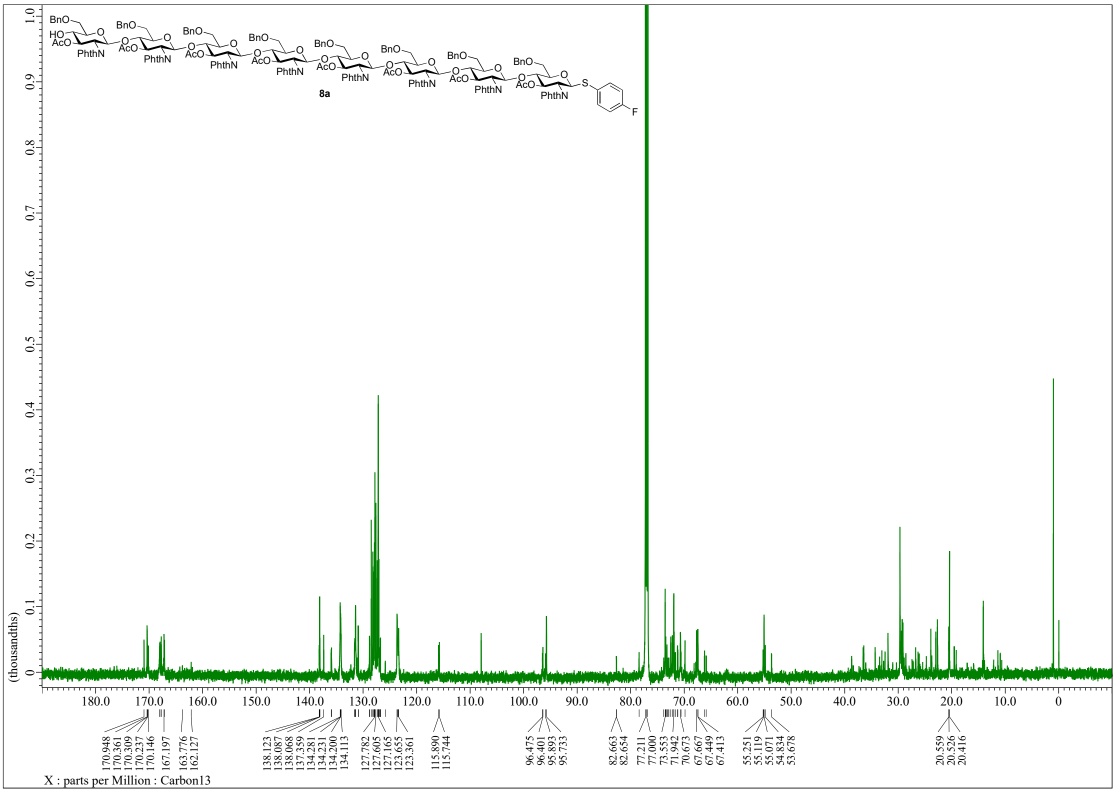

H,H-COSY

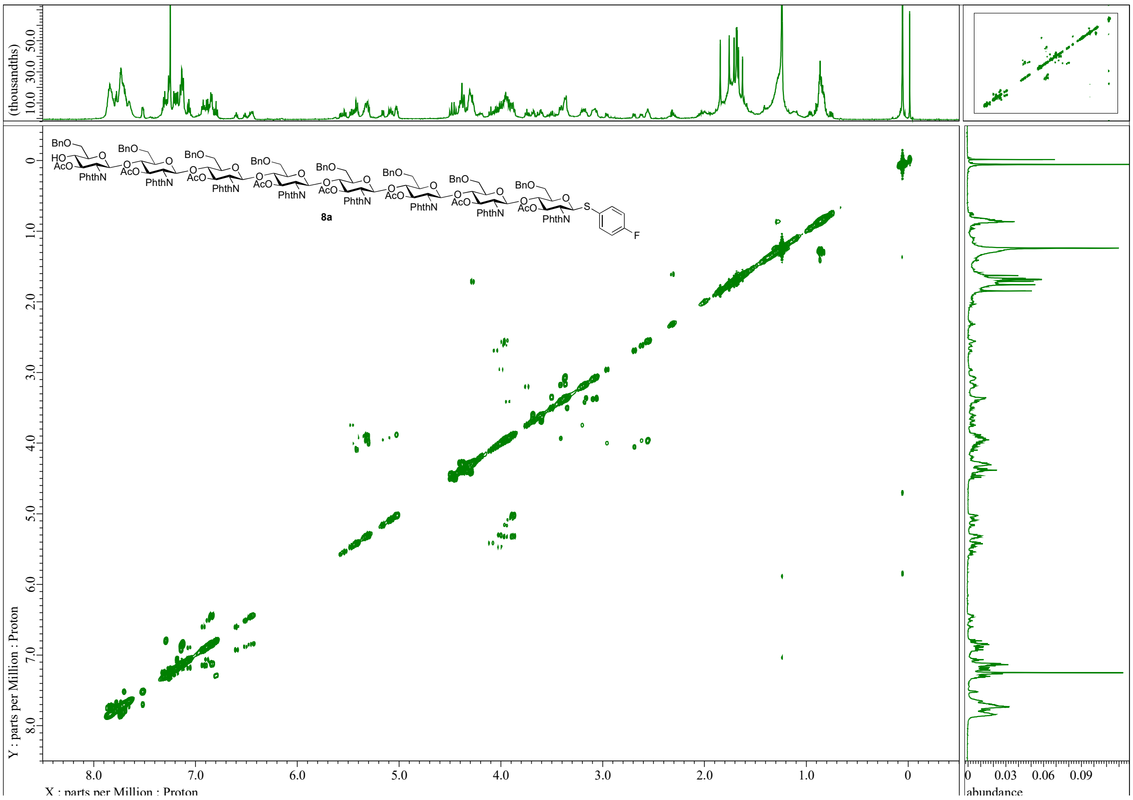

HMQC

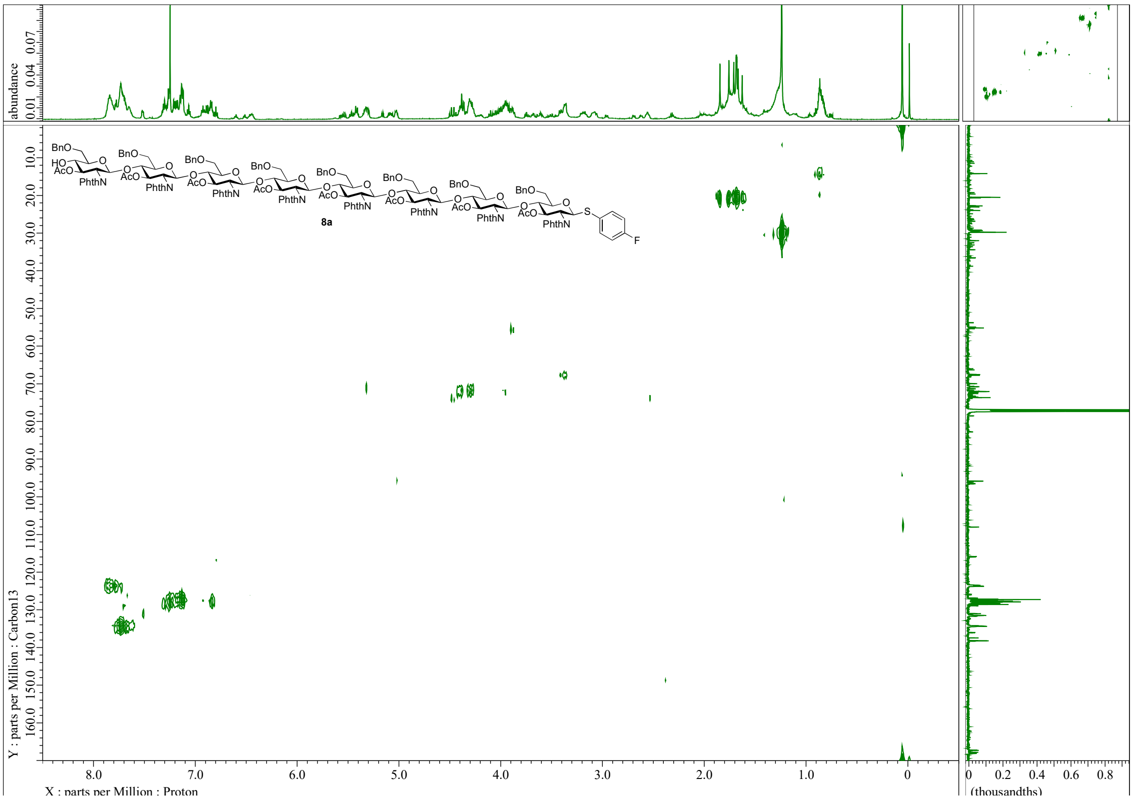

^{1}H NMR

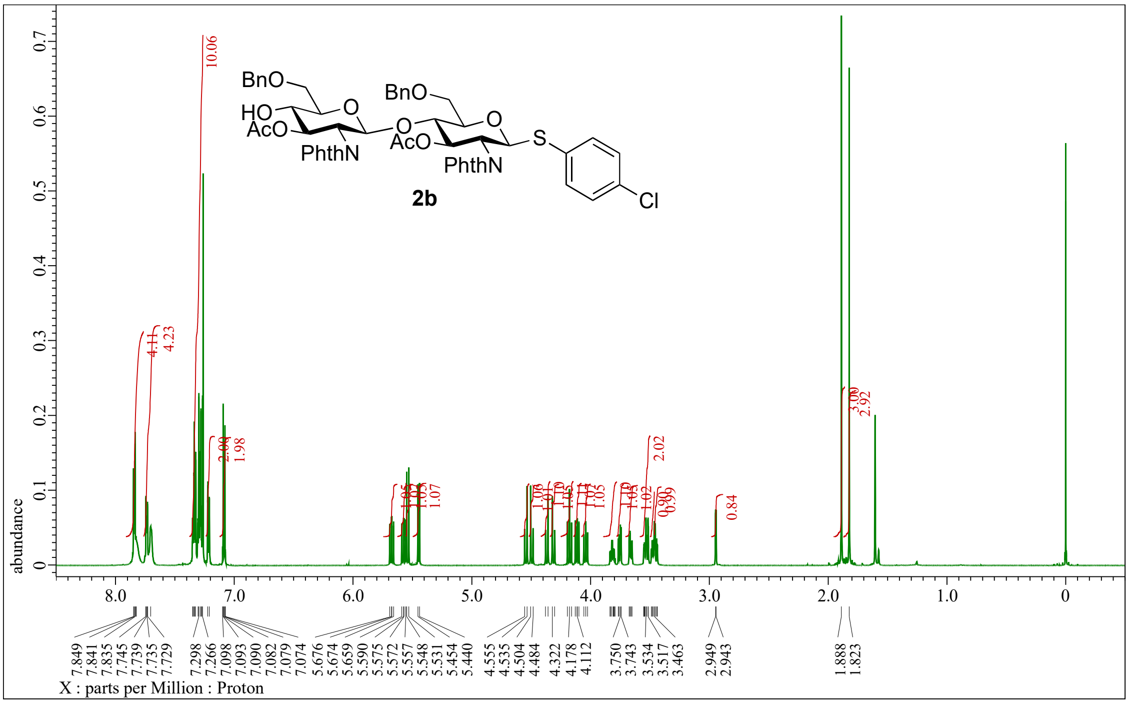

^{13}C NMR

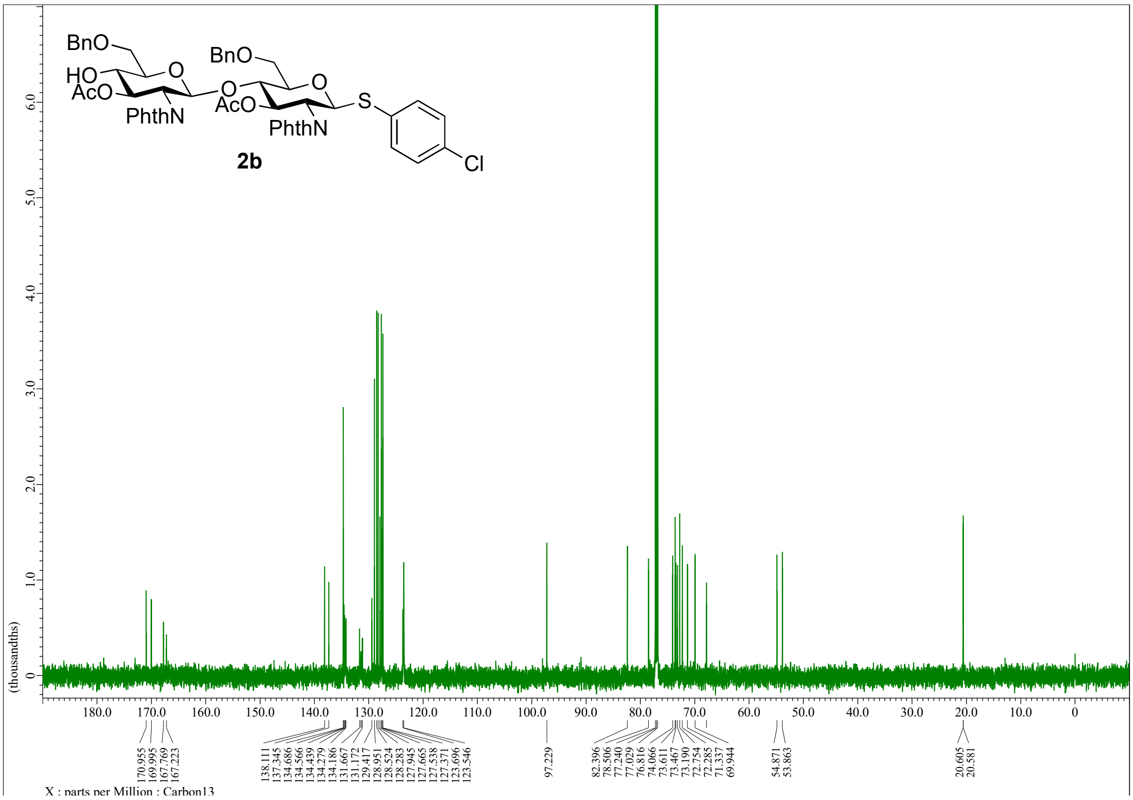

H,H-COSY

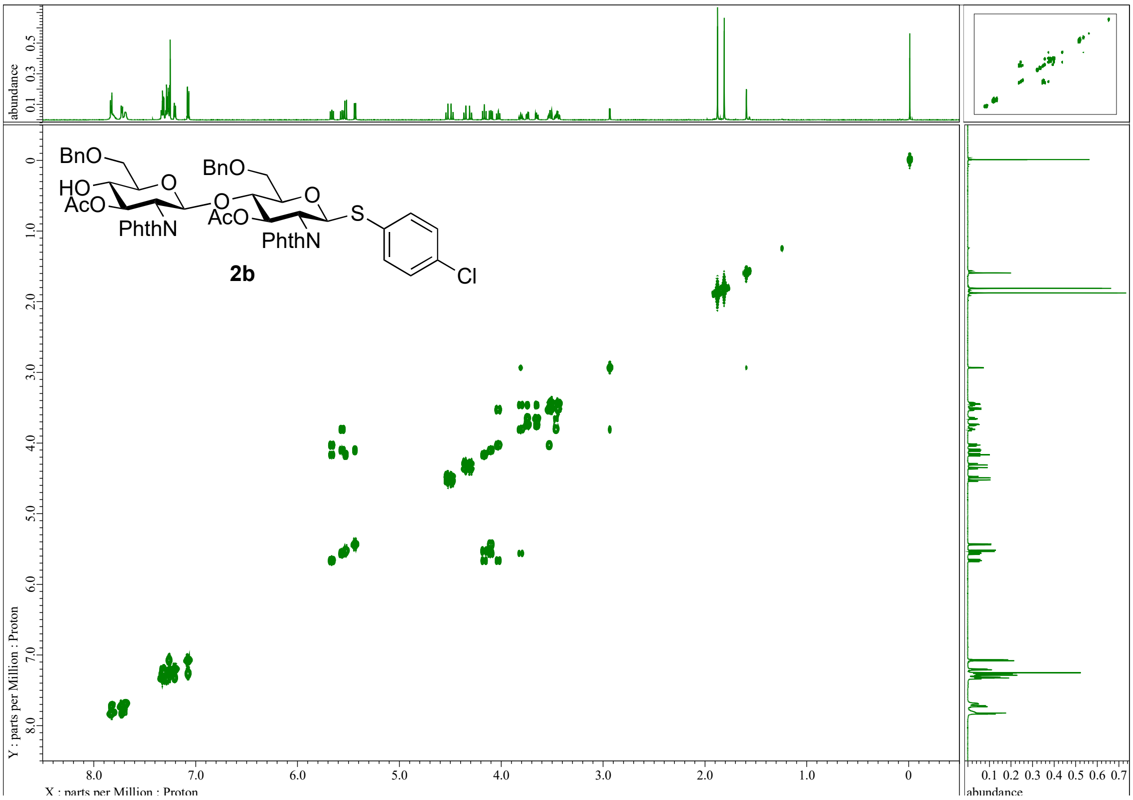
abundance
Y : parts per Million : Proton
X : parts per Million : Proton
BnO
HO
AcO
O
PhthN
AcO
S
Cl
2b
abundance

HMQC

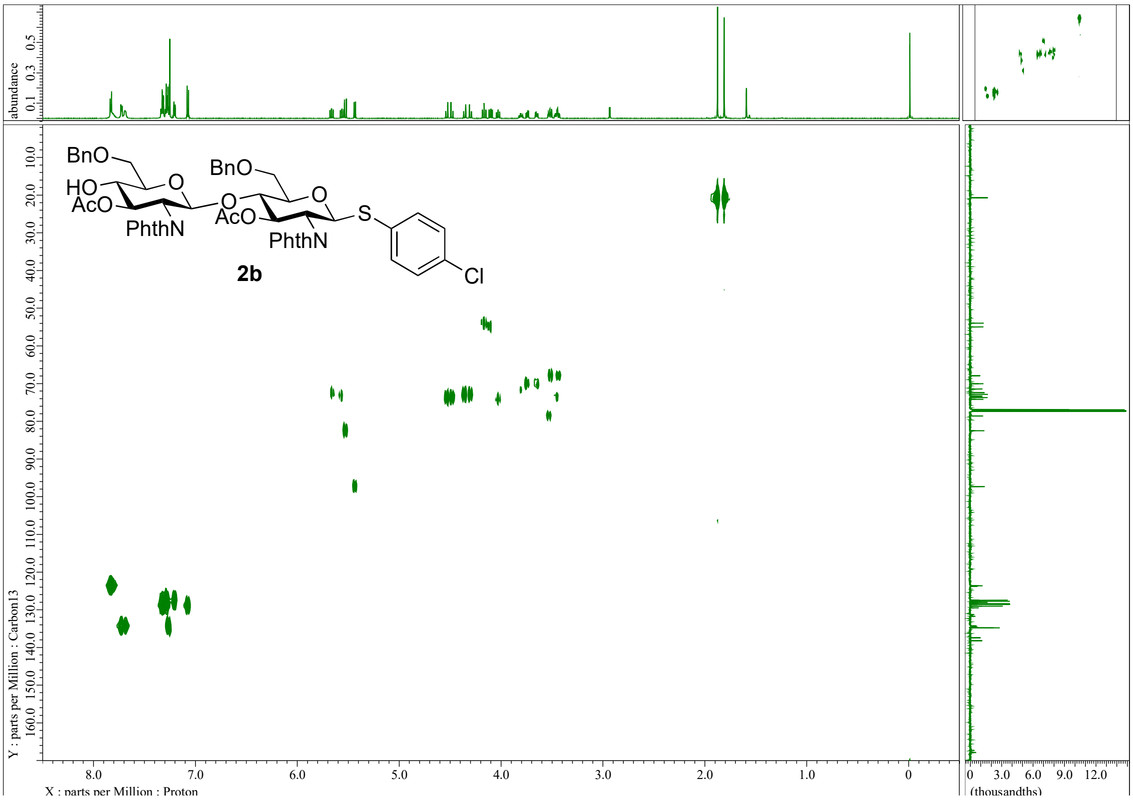
abundance
Y : parts per Million : Carbon13
X : parts per Million : Proton
BnO
HO
AcO
O
PhthN
AcO
S
Cl
2b
(thousandths)

^{1}H NMR

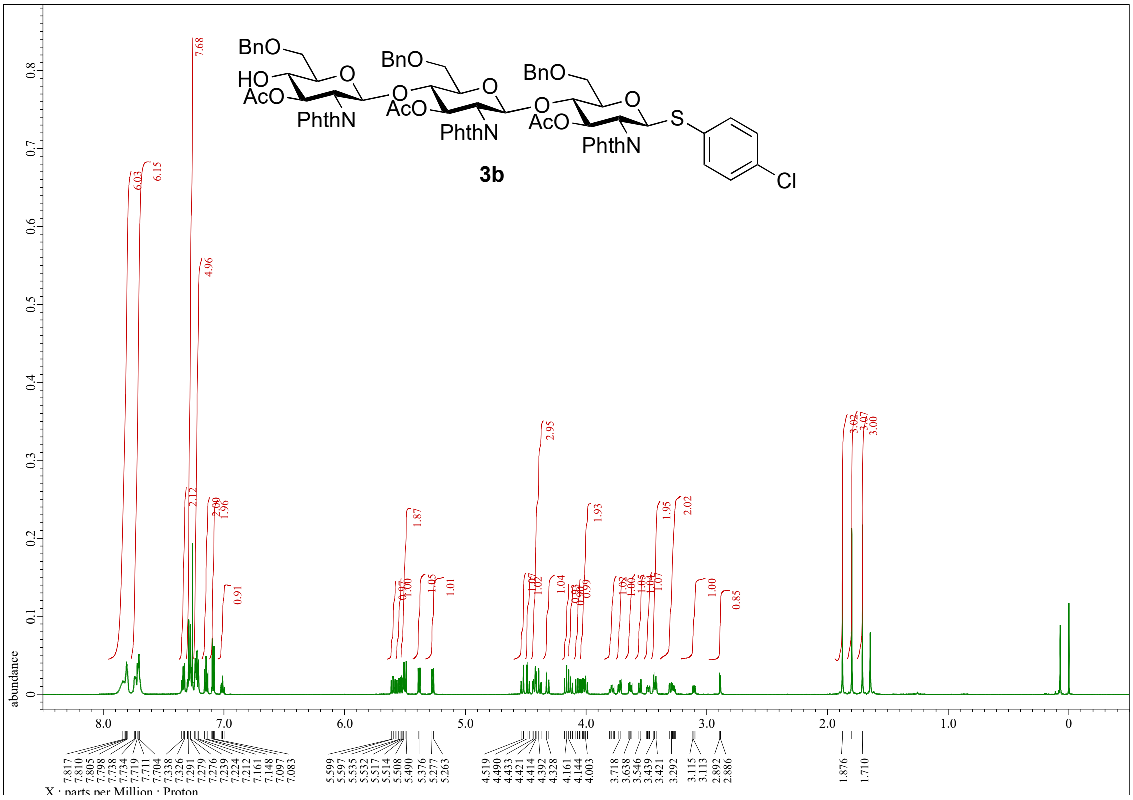

^{13}C NMR

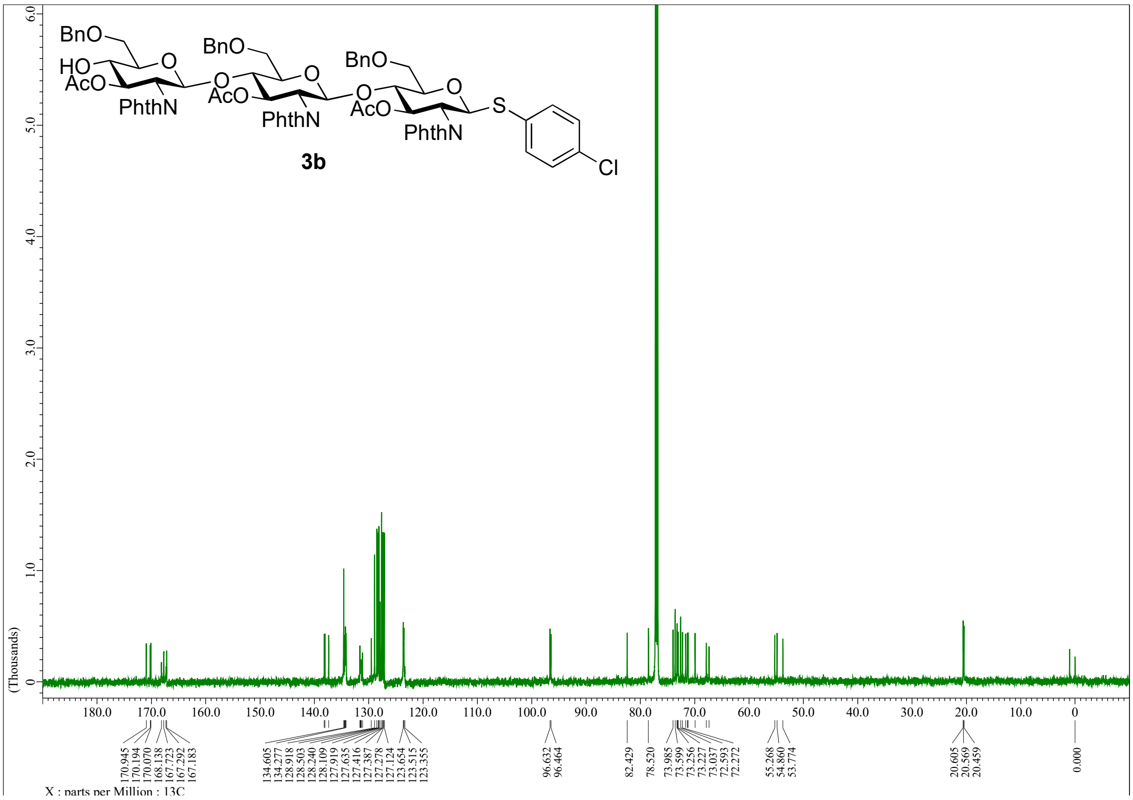

H,H-COSY

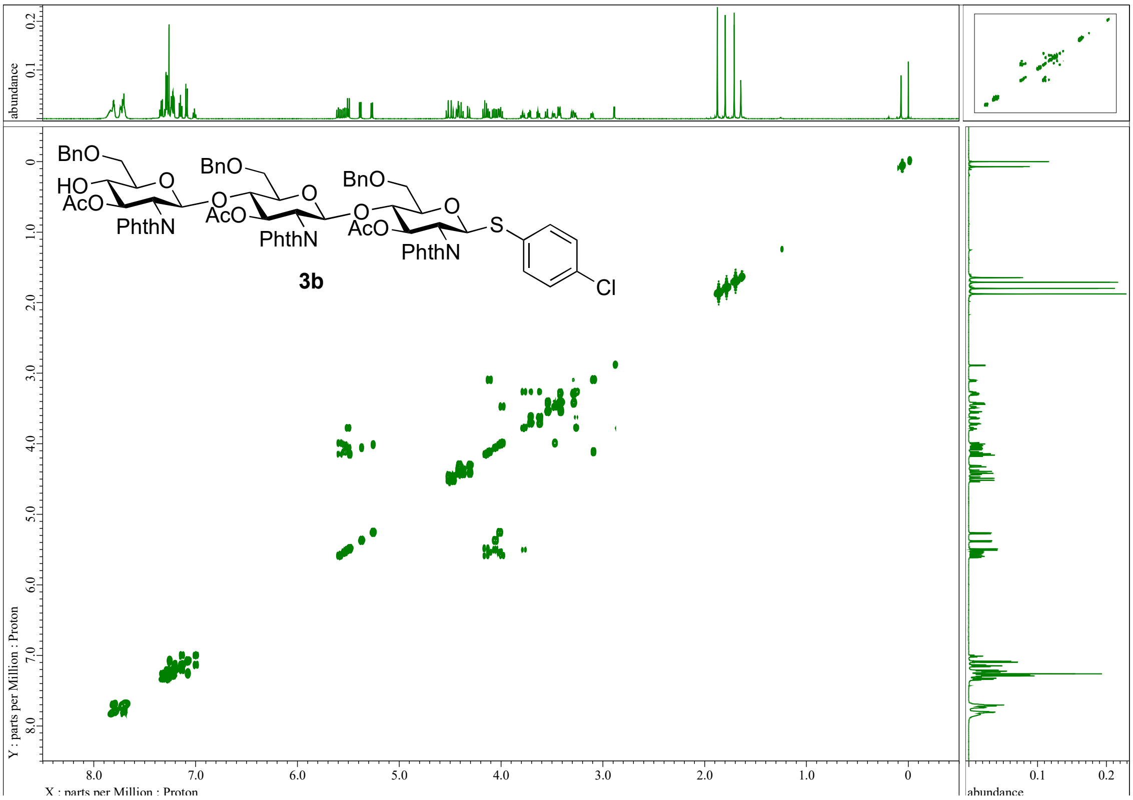
3b
X : parts per Million : Proton
Y : parts per Million : Proton
abundance

HMQC

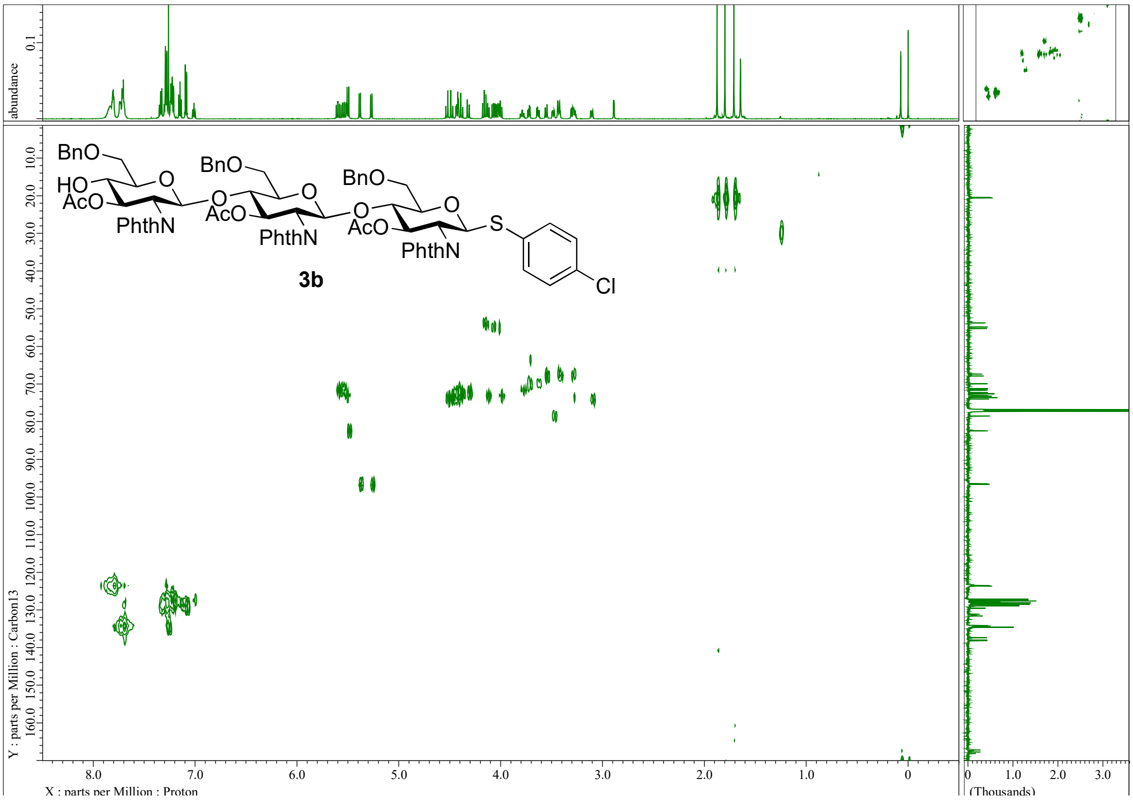
3b
X : parts per Million : Proton
Y : parts per Million : Carbon13
abundance
(Thousands)

^{1}H NMR

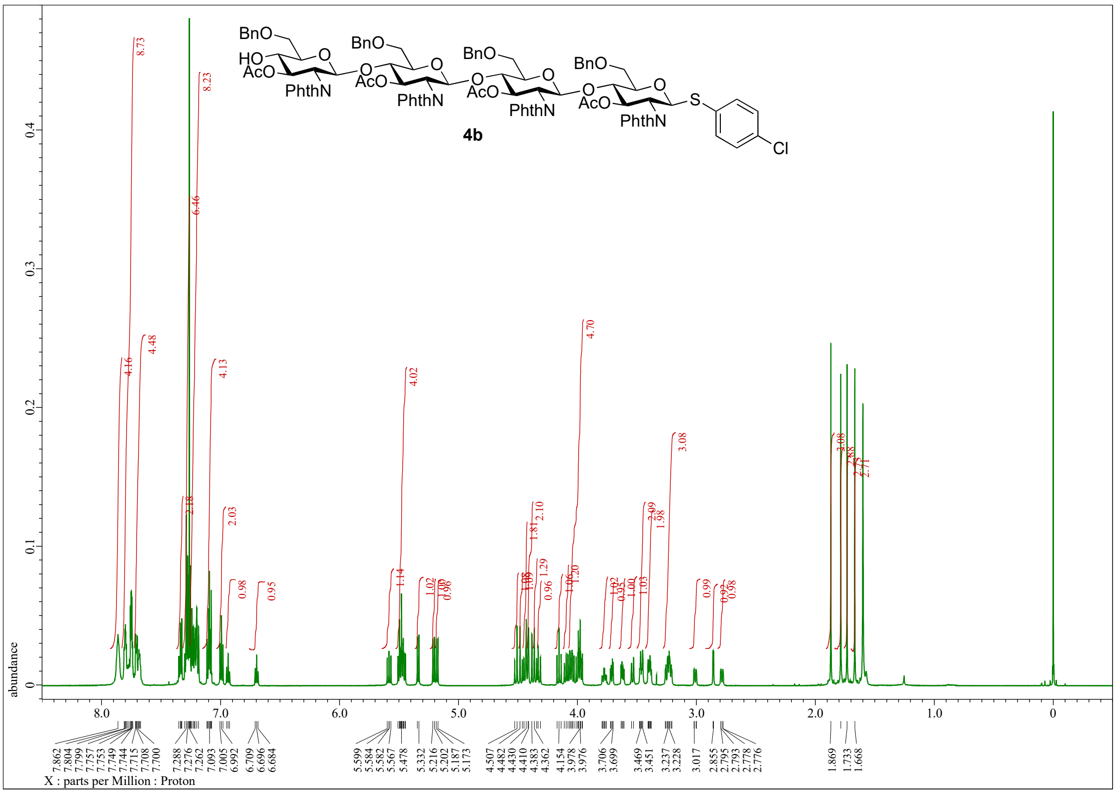

^{13}C NMR

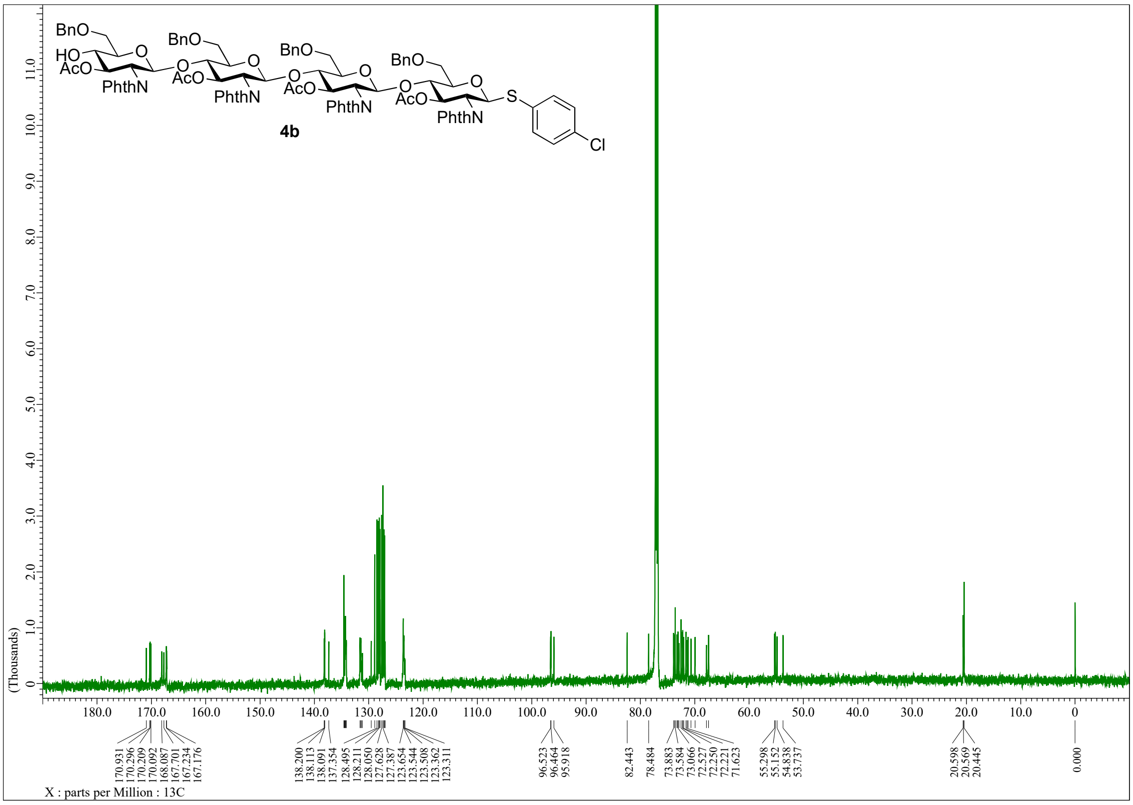

H,H-COSY

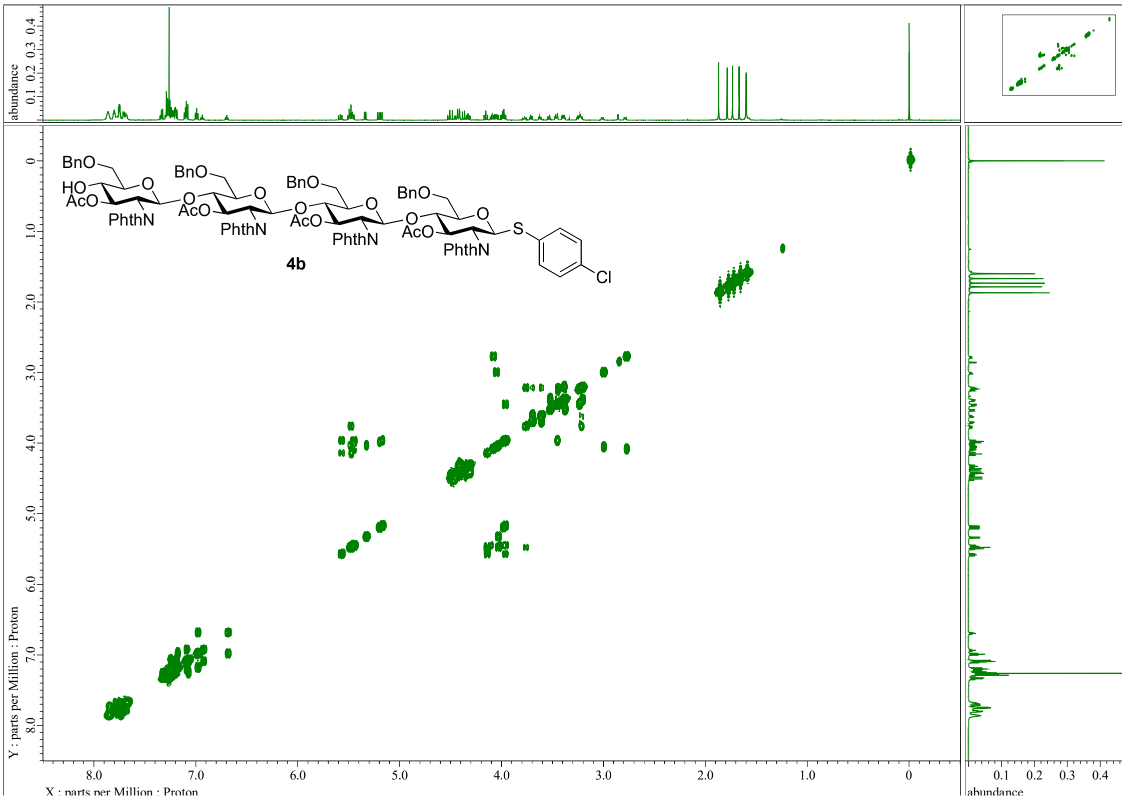
BnO
HO
AcO
PhthN
O
4b
S
Cl
X : parts per Million : Proton
Y : parts per Million : Proton
abundance

HMQC

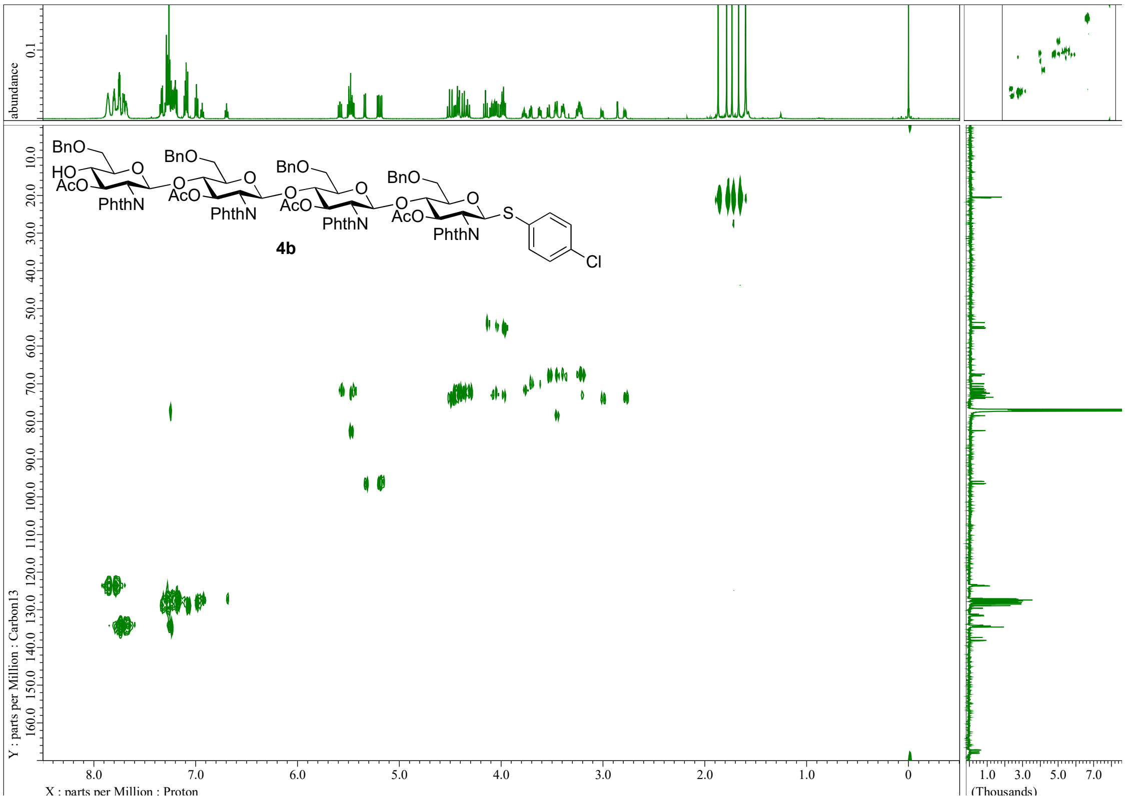
BnO
HO
AcO
PhthN
O
4b
S
Cl
X : parts per Million : Proton
Y : parts per Million : Carbon13
abundance
(Thousands)

^{1}H NMR

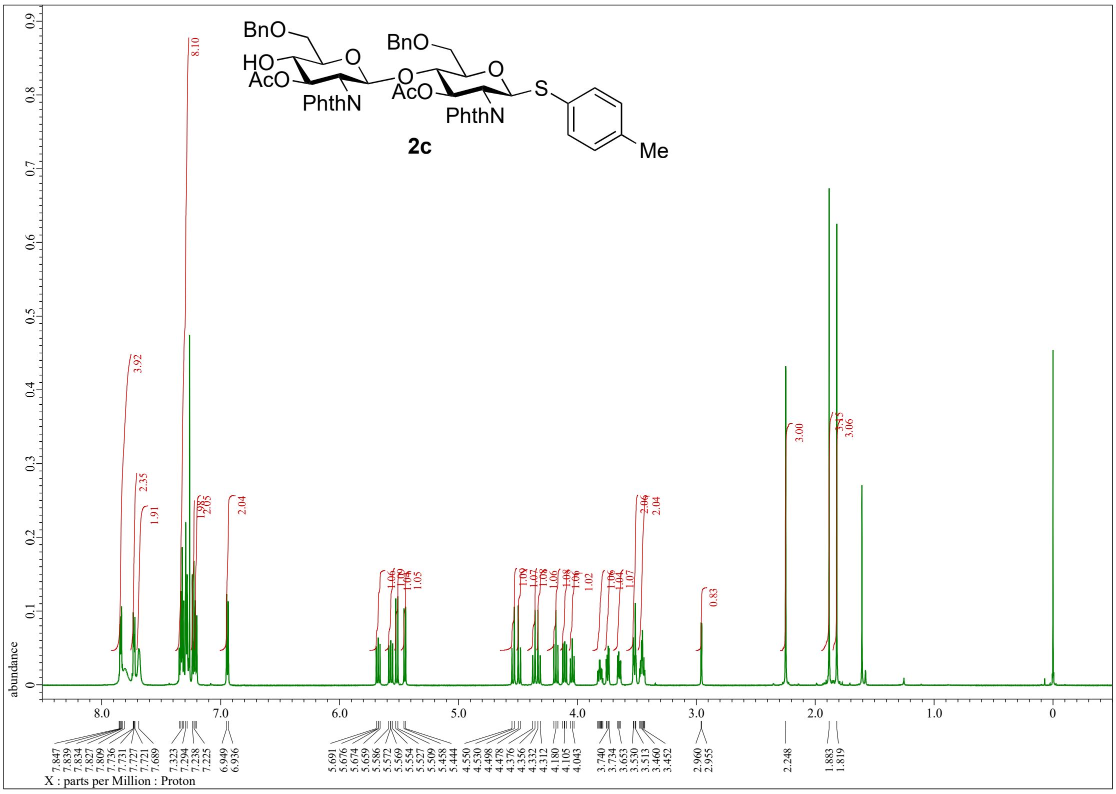

^{13}C NMR

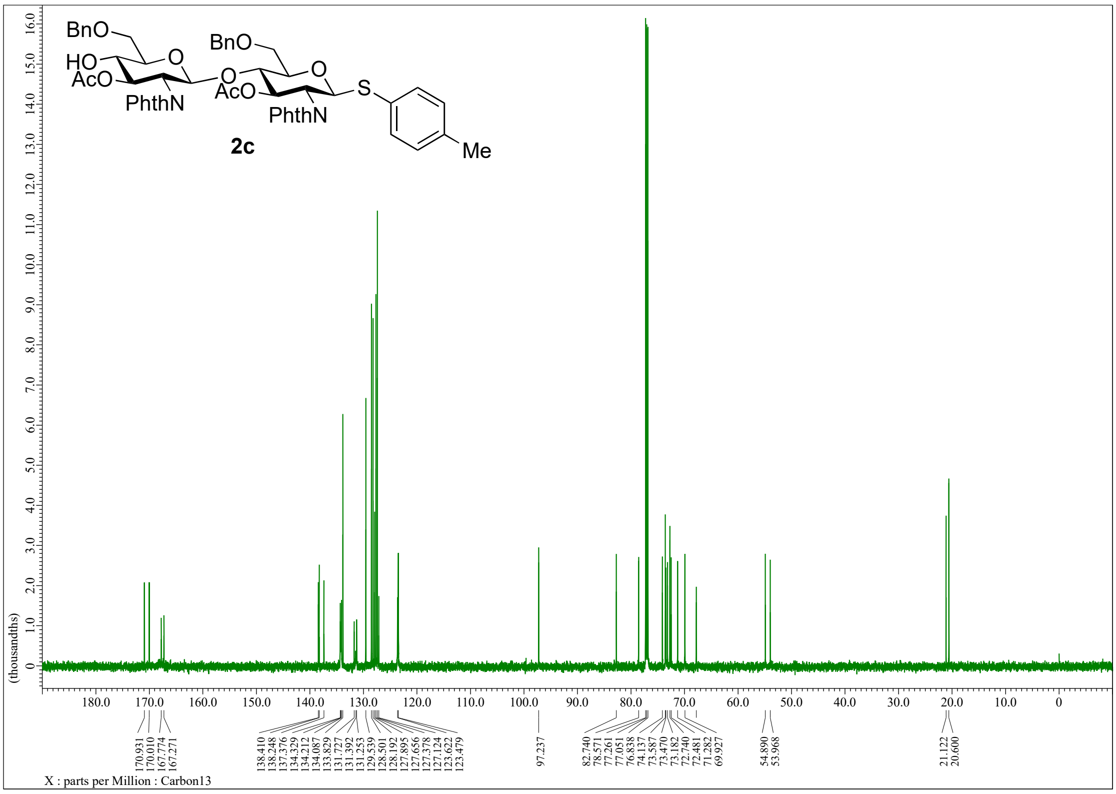

H,H-COSY

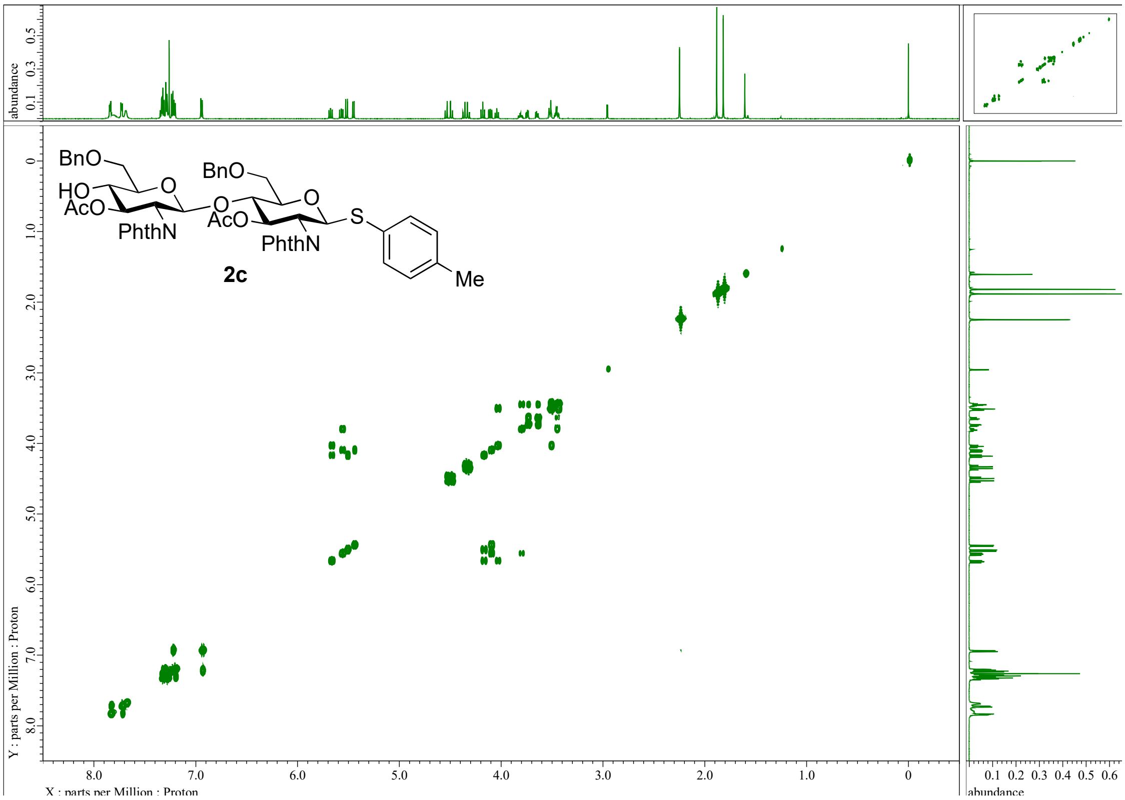
BnO
HO
AcO
O
PhthN
BnO
O
AcO
O
S
PhthN
Me
2c
abundance
Y : parts per Million : Proton
X : parts per Million : Proton
abundance

HMQC

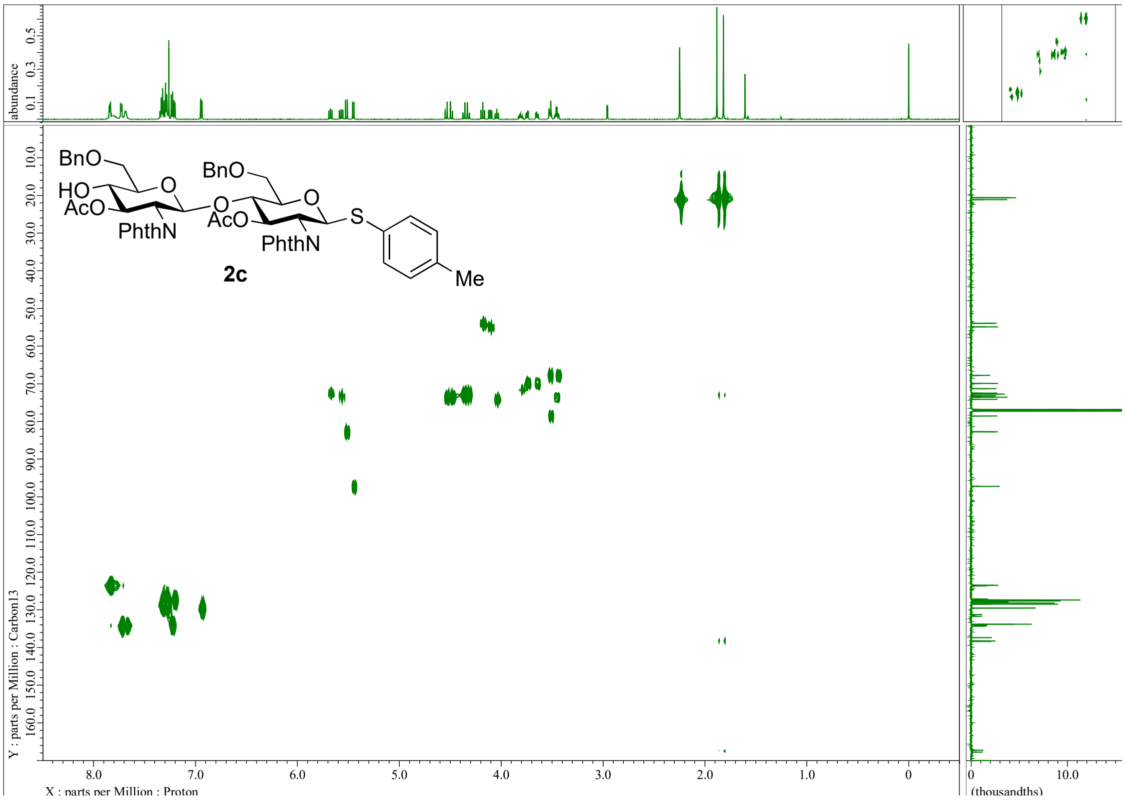
BnO
HO
AcO
O
PhthN
BnO
O
AcO
O
S
PhthN
Me
2c
abundance
Y : parts per Million : Carbon13
X : parts per Million : Proton
(thousandths)

^{1}H NMR

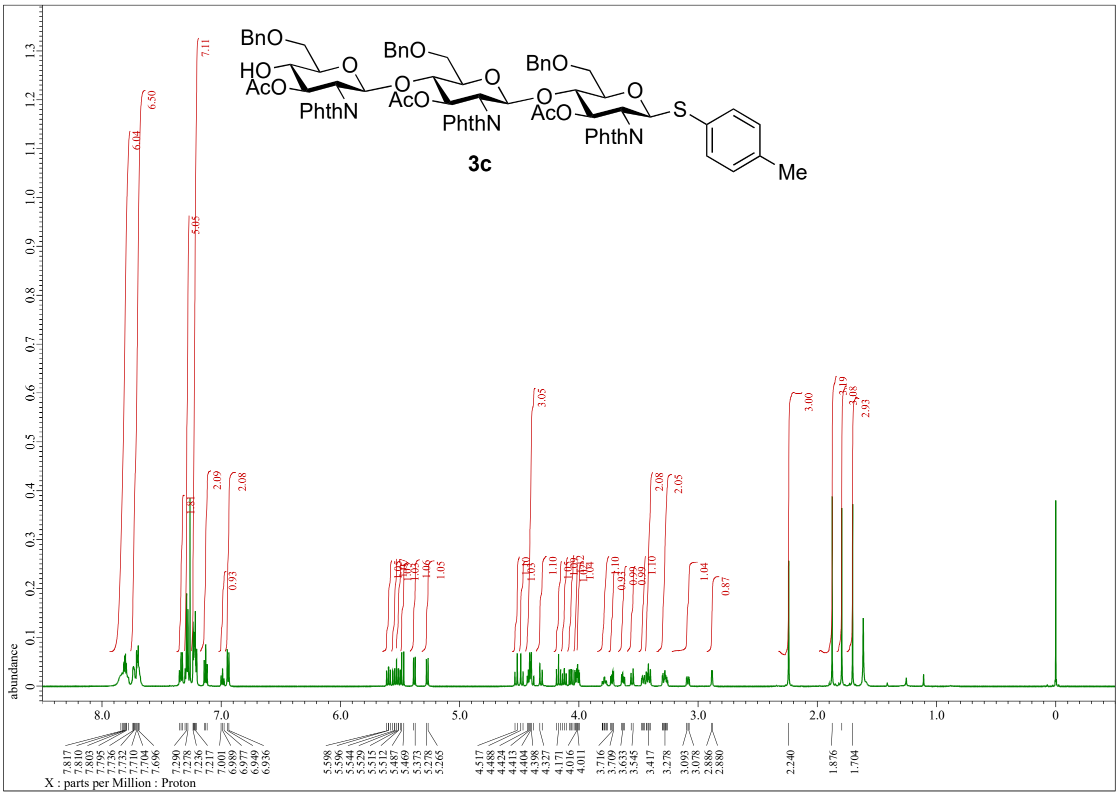

^{13}C NMR

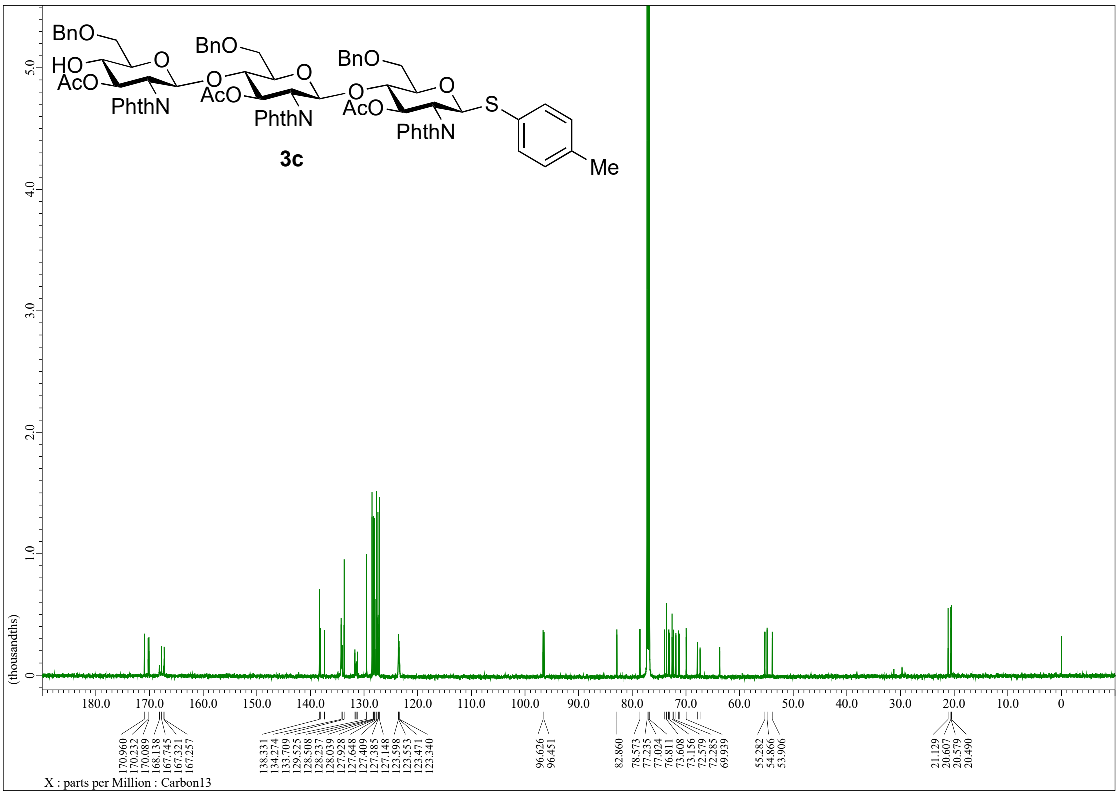

H,H-COSY

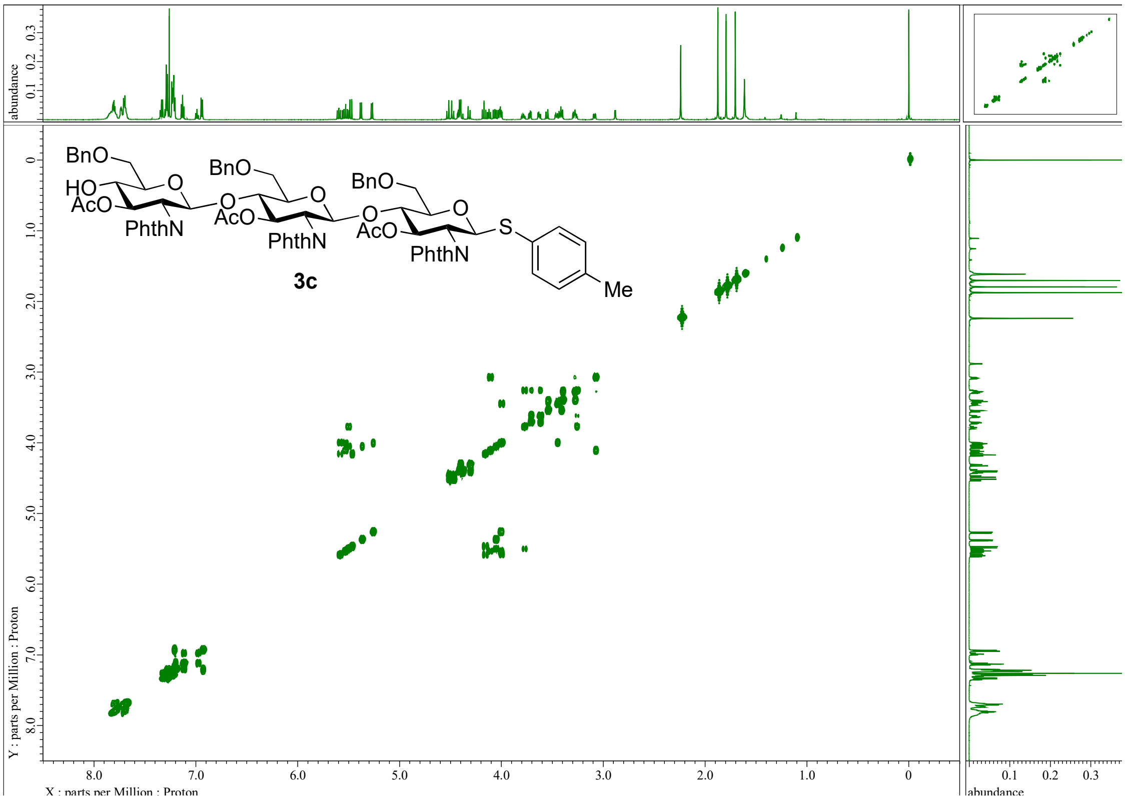
BnO
HO
AcO
PhthN
BnO
AcO
PhthN
BnO
AcO
PhthN
S
Me
3c
abundance
X : parts per Million : Proton
Y : parts per Million : Proton

HMQC

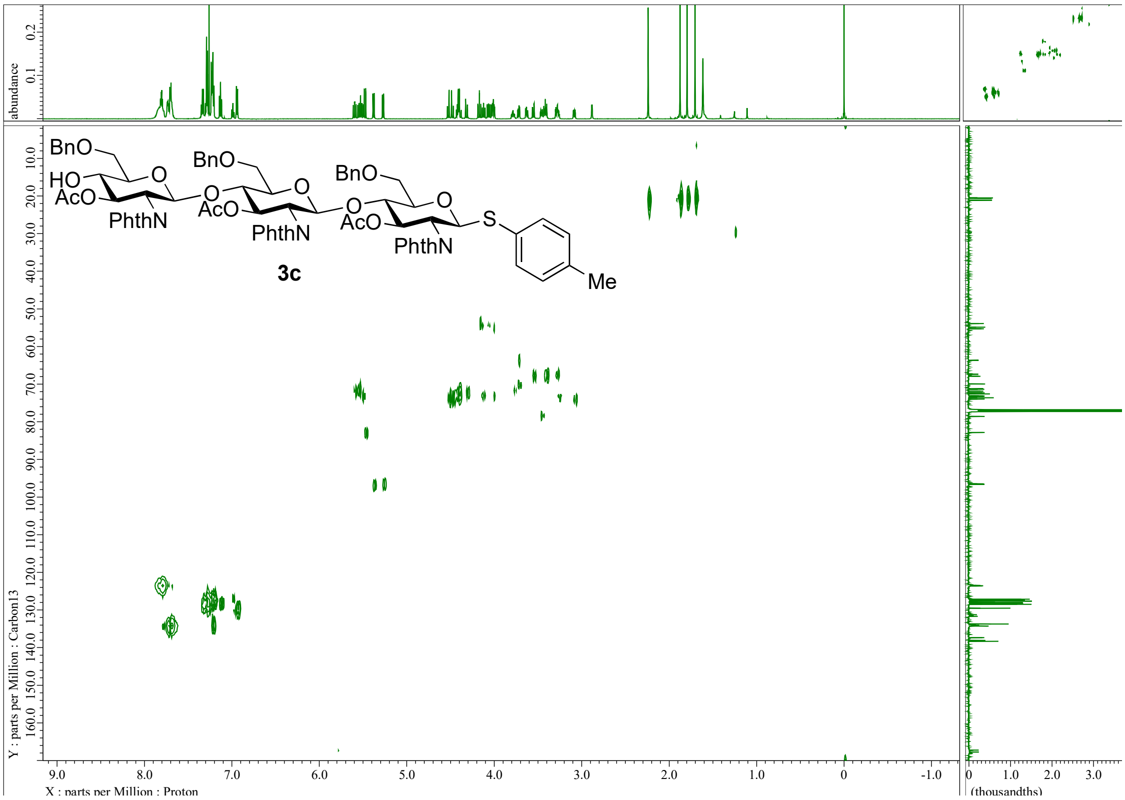
BnO
HO
AcO
PhthN
BnO
AcO
PhthN
BnO
AcO
PhthN
S
Me
3c
abundance
X : parts per Million : Proton
Y : parts per Million : Carbon13
(thousandths)

^{1}H NMR

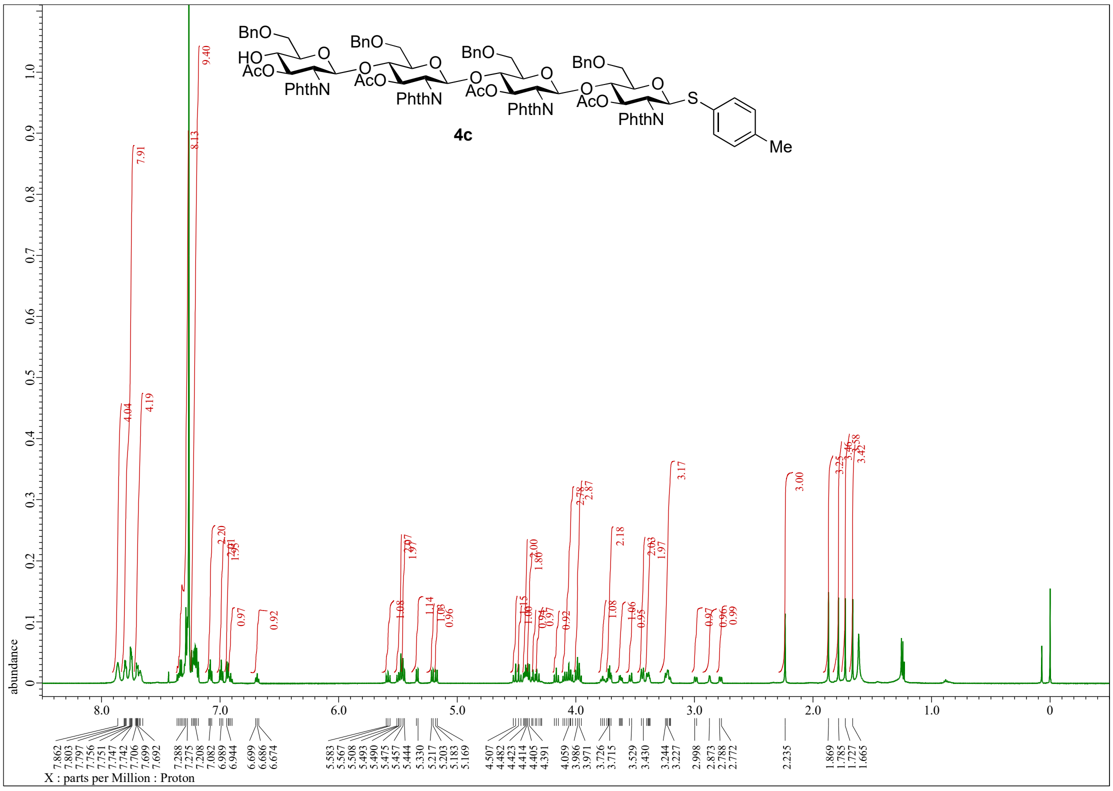

^{13}C NMR

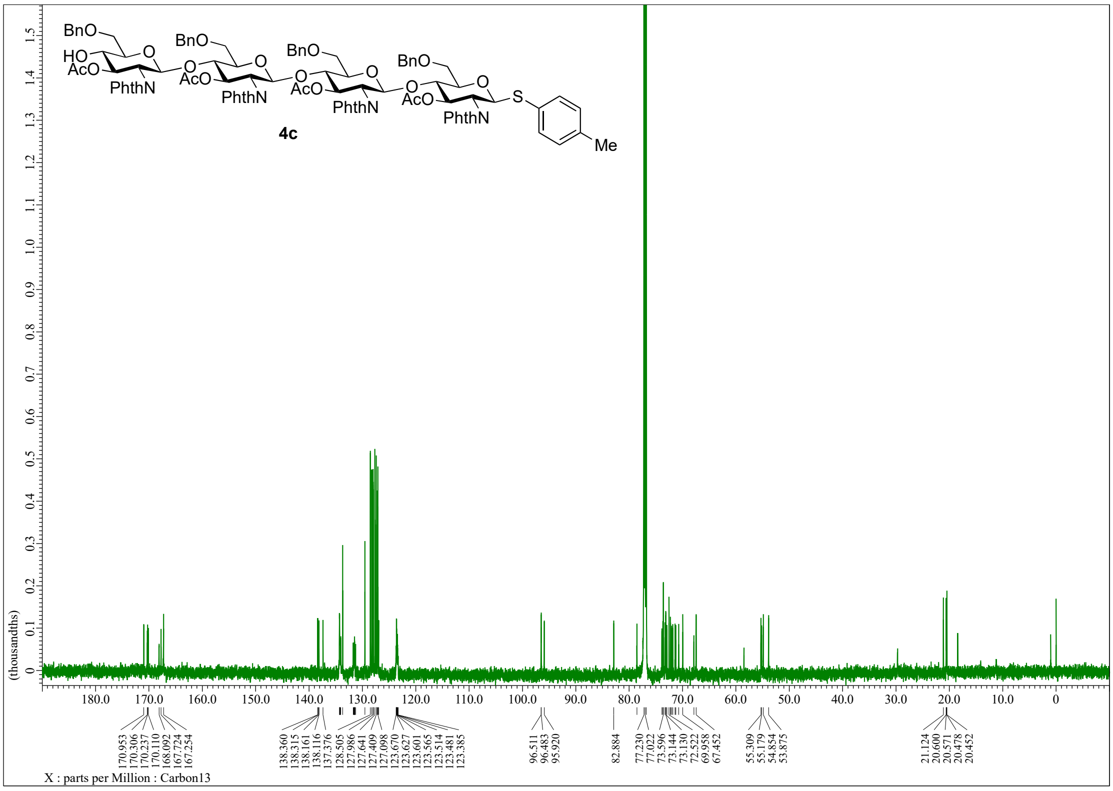

H,H-COSY

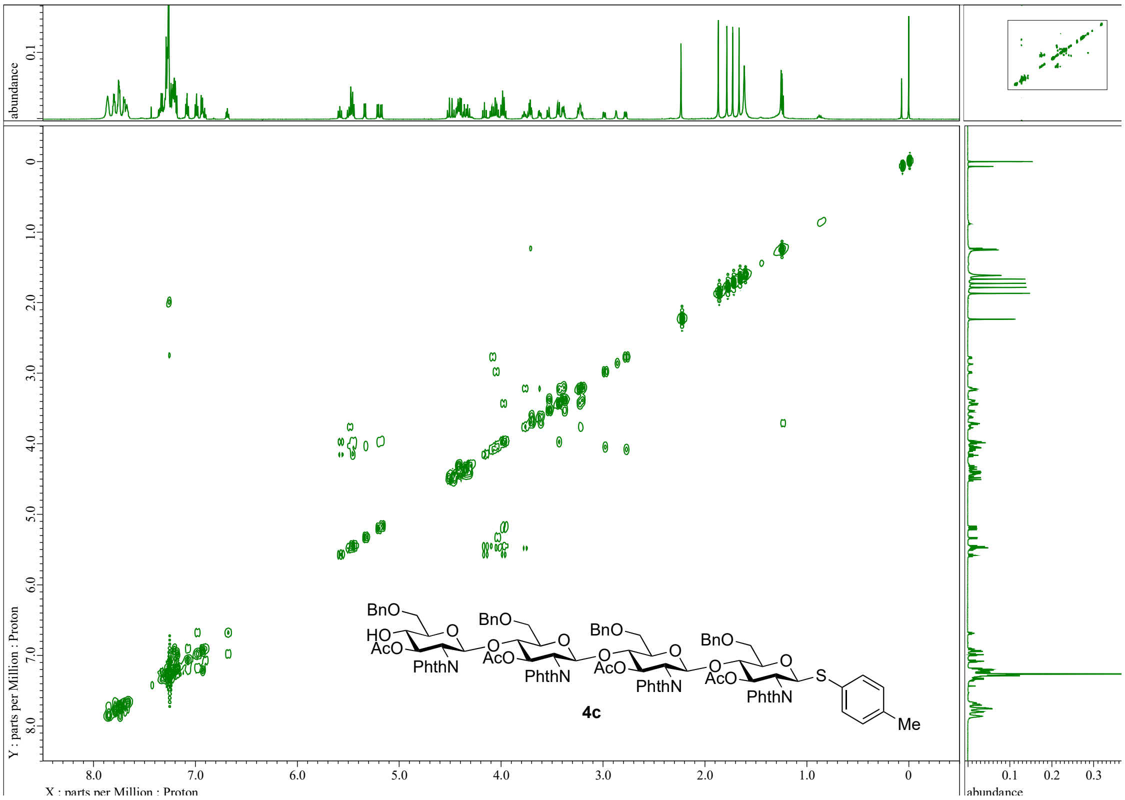
abundance
Y : parts per Million : Proton
X : parts per Million : Proton
abundance
BnO
HO
AcO
PhthN
O
S
Me
4c

HMQC

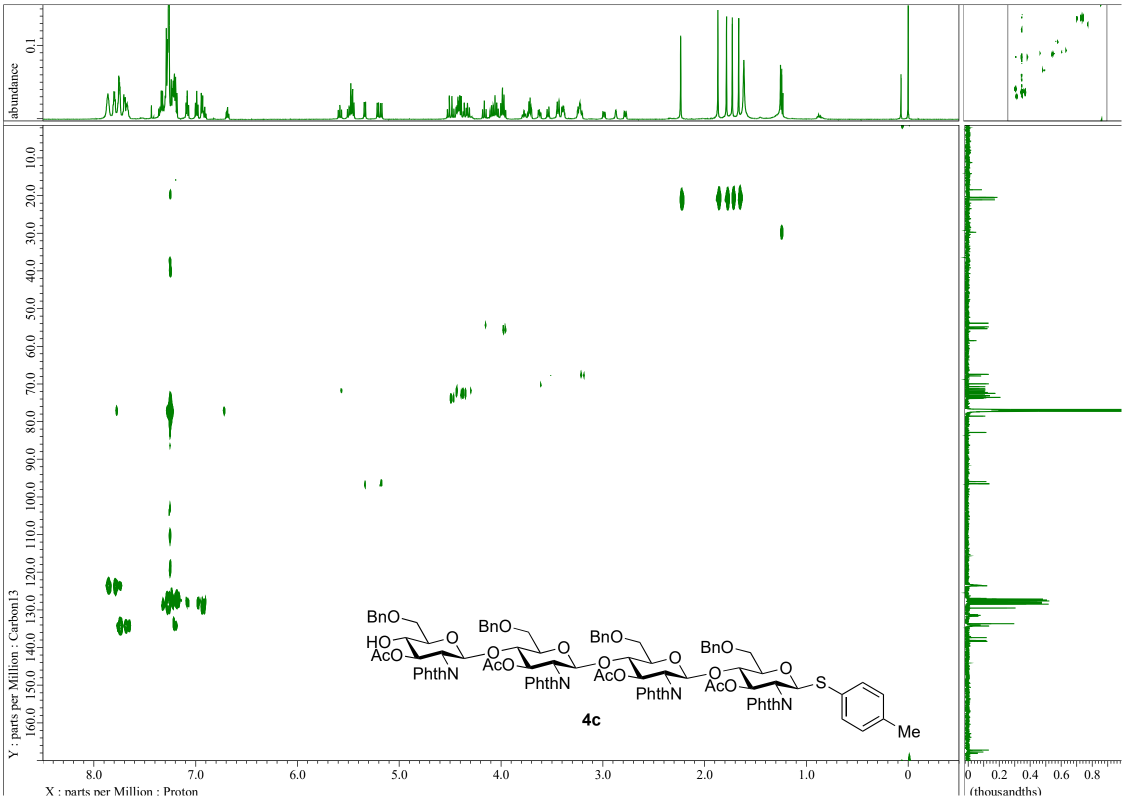
abundance
Y : parts per Million : Carbon13
X : parts per Million : Proton
(thousandths)
BnO
HO
AcO
PhthN
O
S
Me
4c

^1H NMR

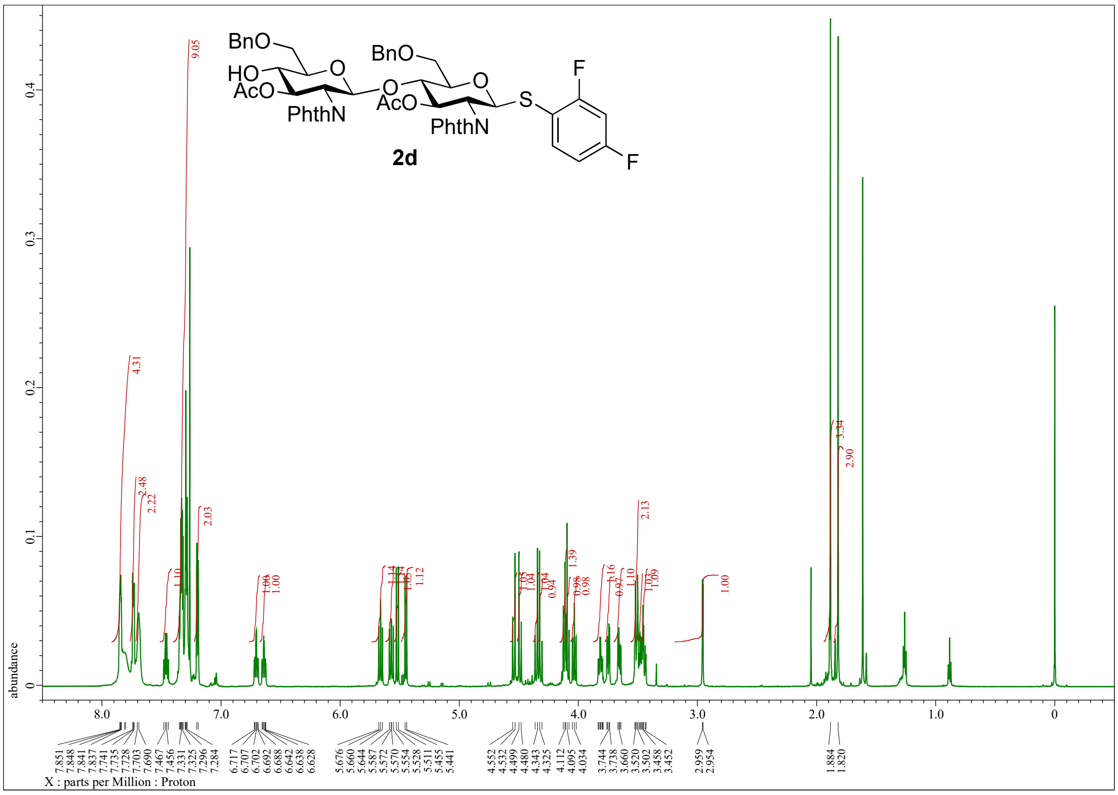

^{13}C NMR

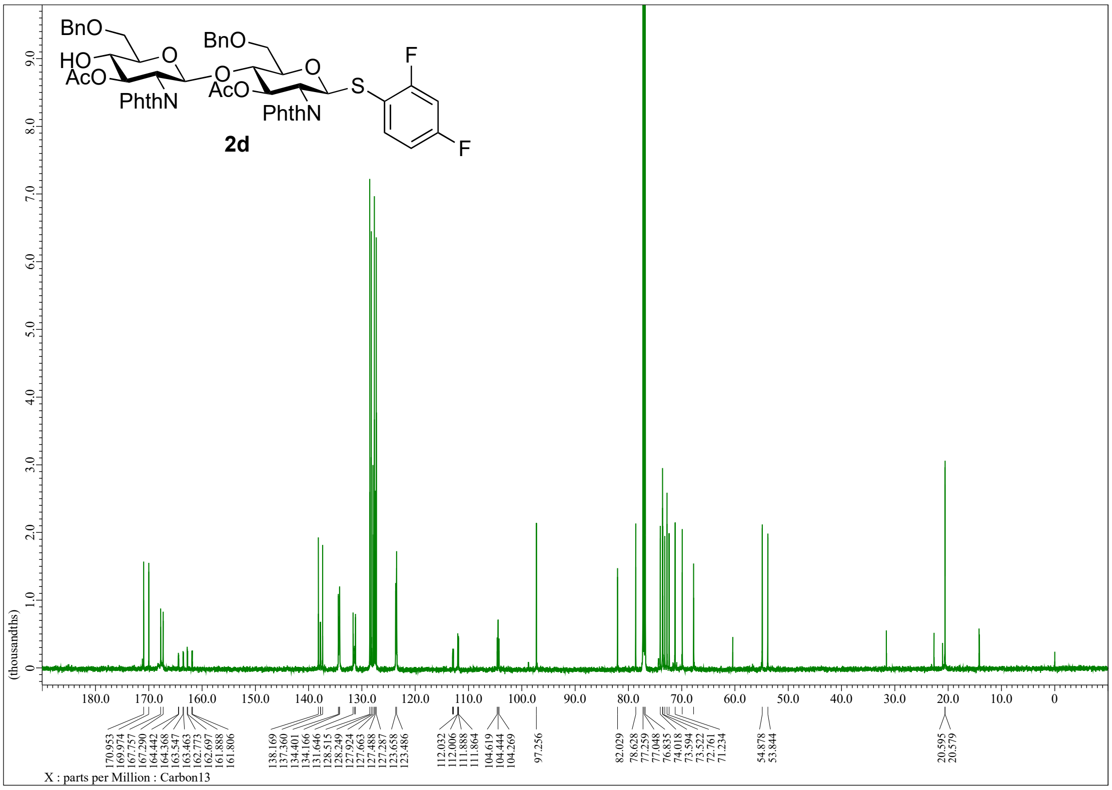

H,H-COSY

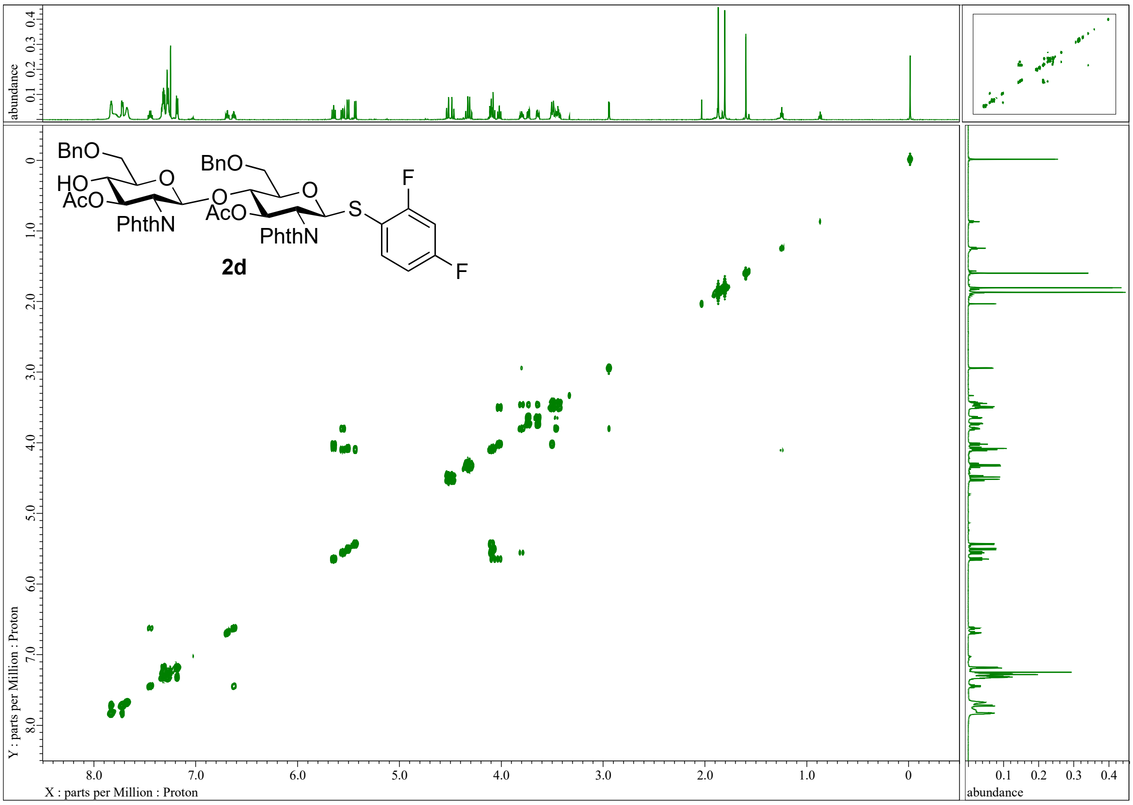
BnO
HO
AcO
O
PhthN
BnO
O
AcO
O
PhthN
S
F
F
2d
abundance
X : parts per Million : Proton
Y : parts per Million : Proton

HMQC

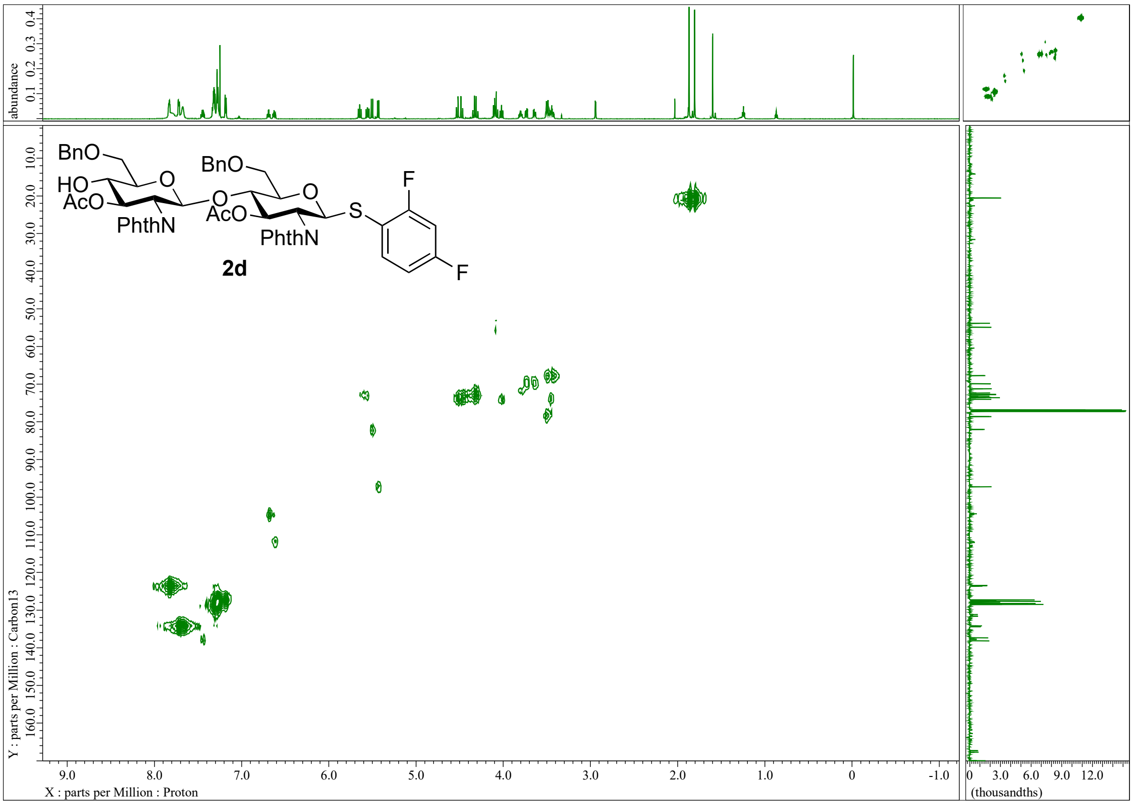
BnO
HO
AcO
O
PhthN
BnO
O
AcO
O
PhthN
S
F
F
2d
abundance
X : parts per Million : Proton
Y : parts per Million : Carbon13
(thousandths)

^{1}H NMR

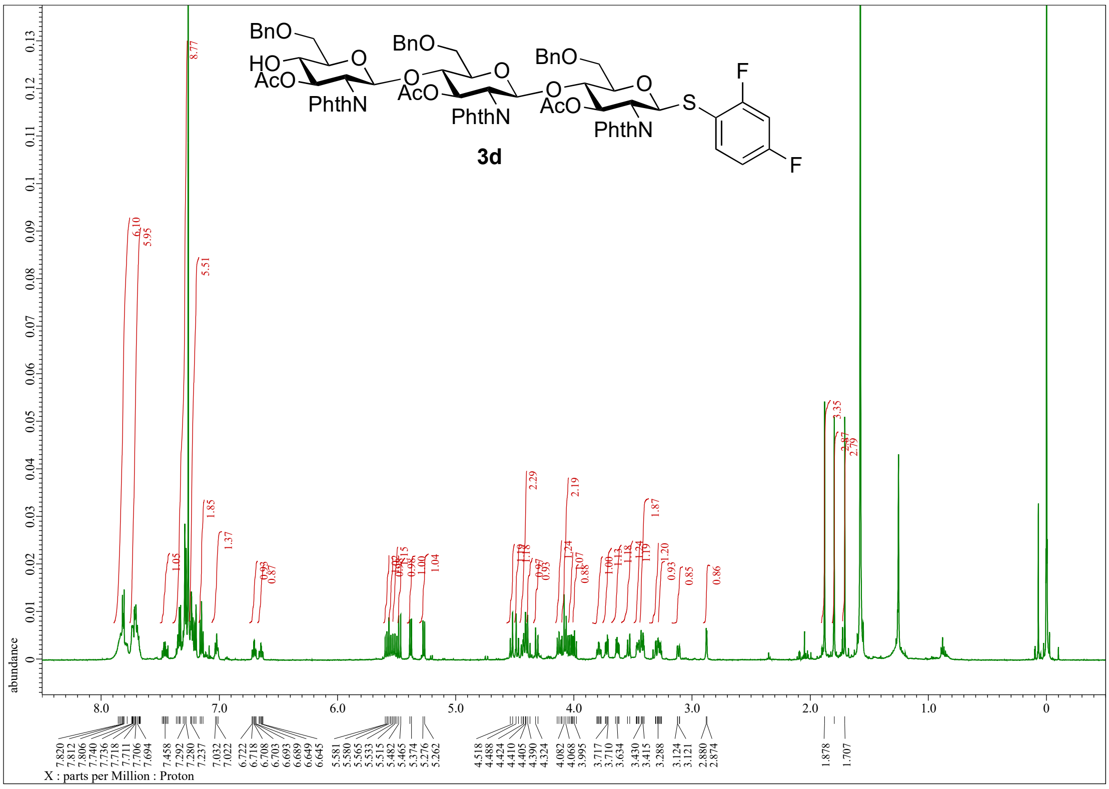

^{13}C NMR

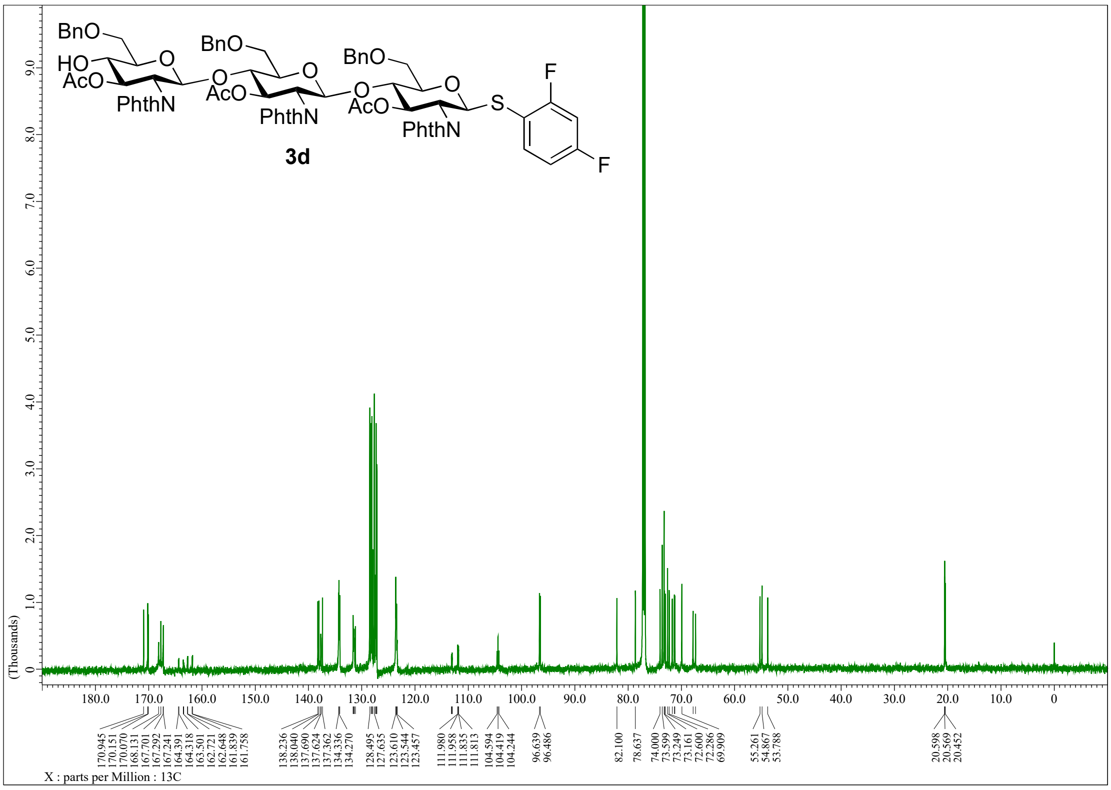

H,H-COSY

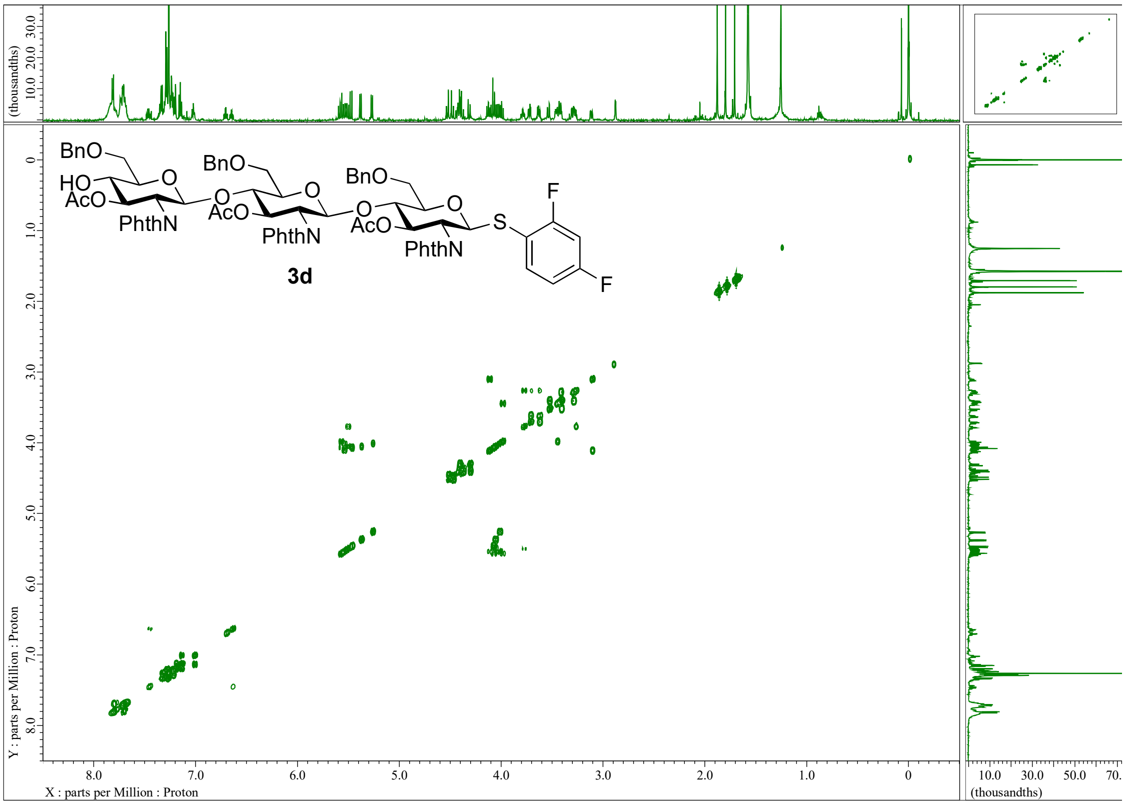
BnO
HO
AcO
O
PhthN
BnO
O
AcO
O
PhthN
BnO
O
AcO
O
PhthN
S
F
F
3d
(thousandths)
Y : parts per Million : Proton
X : parts per Million : Proton
(thousandths)

HMQC

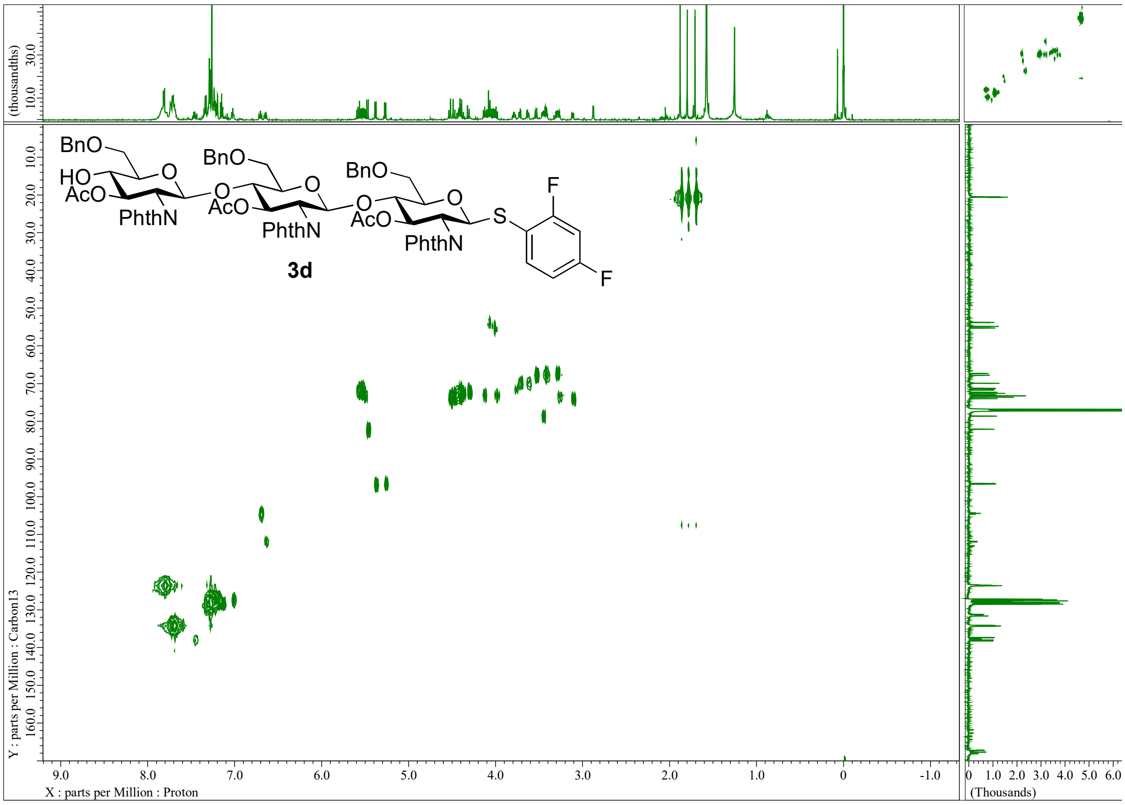
BnO
HO
AcO
O
PhthN
BnO
O
AcO
O
PhthN
BnO
O
AcO
O
PhthN
S
F
F
3d
(thousandths)
Y : parts per Million : Carbon13
X : parts per Million : Proton
(Thousands)

^{1}H NMR

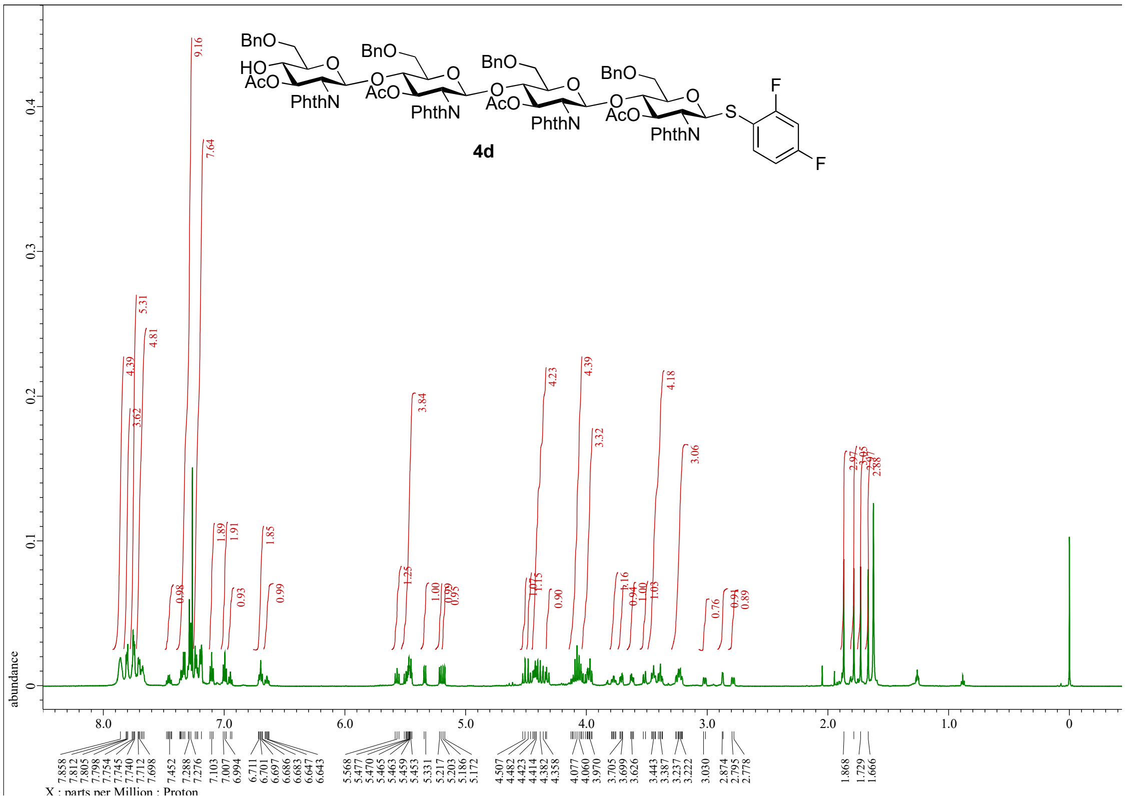

^{13}C NMR

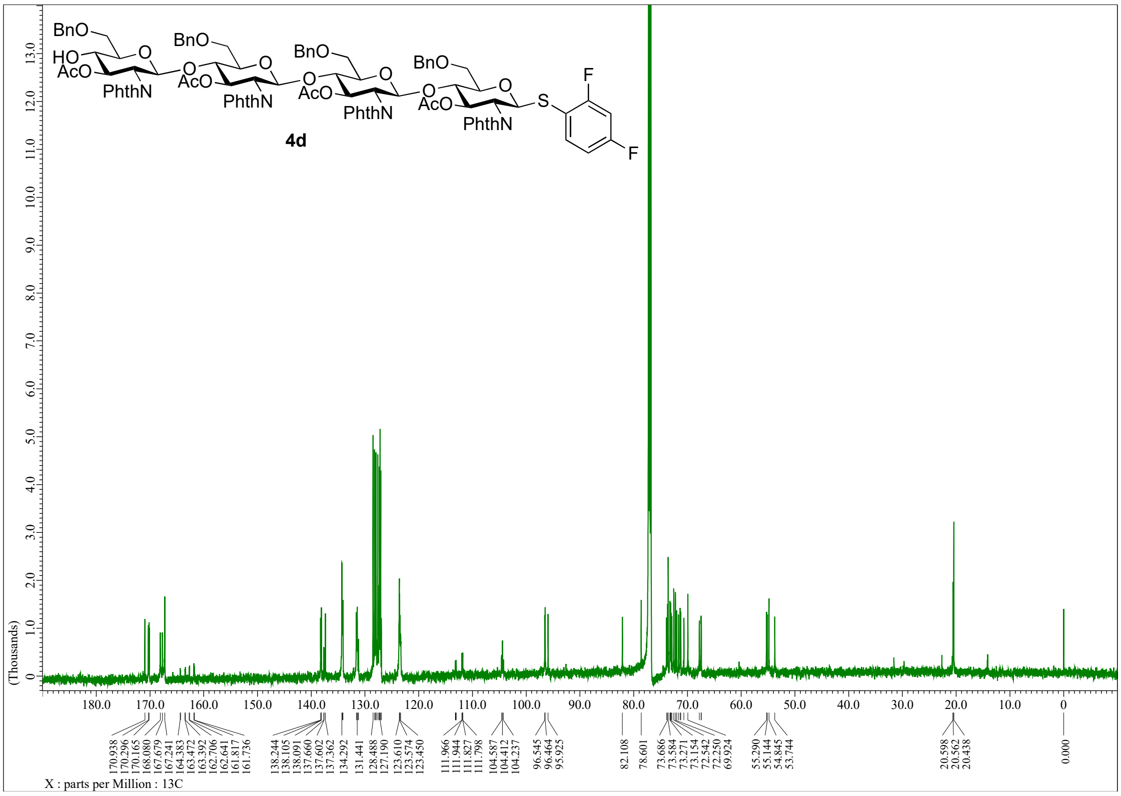

H,H-COSY

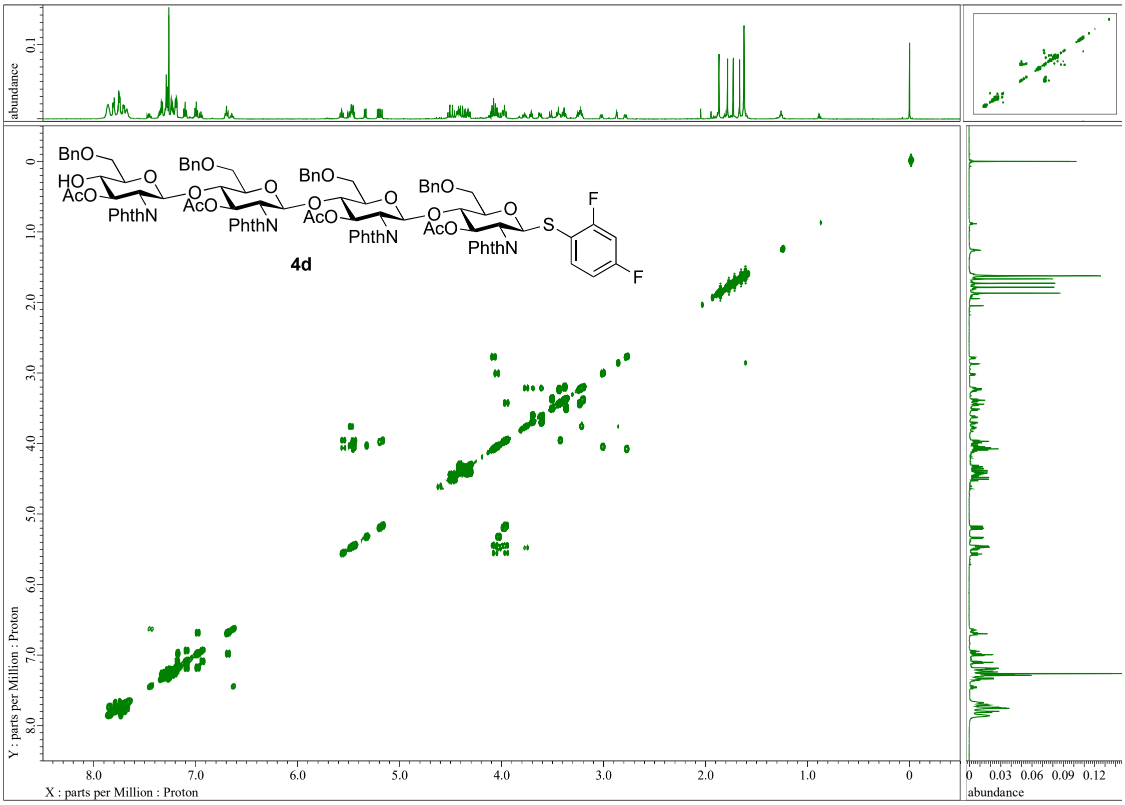
abundance
0.1
BnO
HO
AcO
O
PhthN
AcO
S
F
4d
Y : parts per Million : Proton
X : parts per Million : Proton
0.03 0.06 0.09 0.12

HMQC

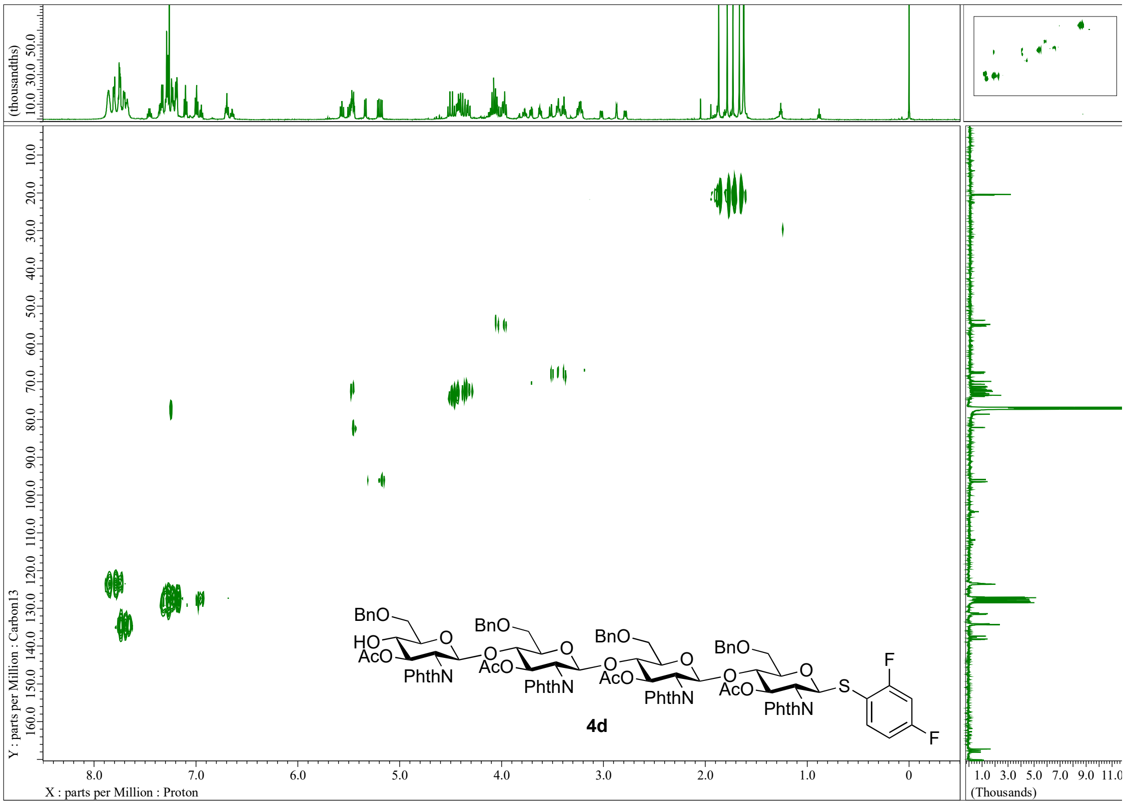
(thousandths)
BnO
HO
AcO
O
PhthN
S
F
4d
Y : parts per Million : Carbon13
X : parts per Million : Proton
(Thousands)

^{1}H NMR

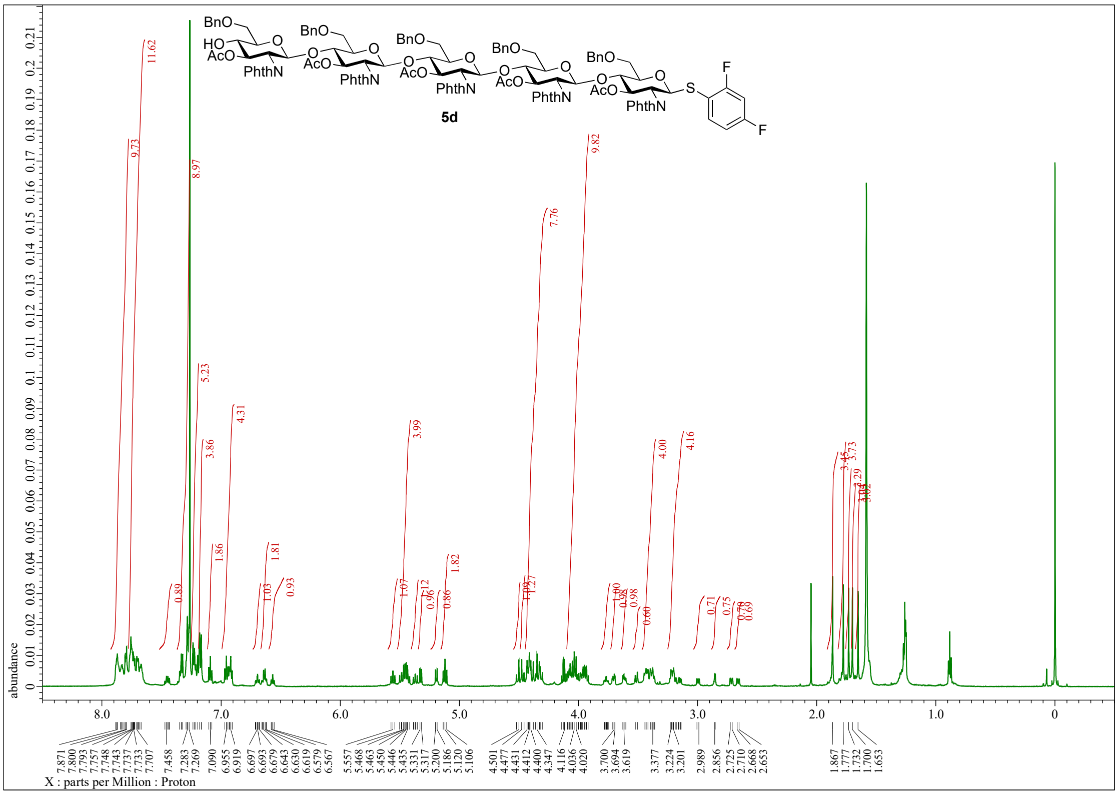

^{13}C NMR

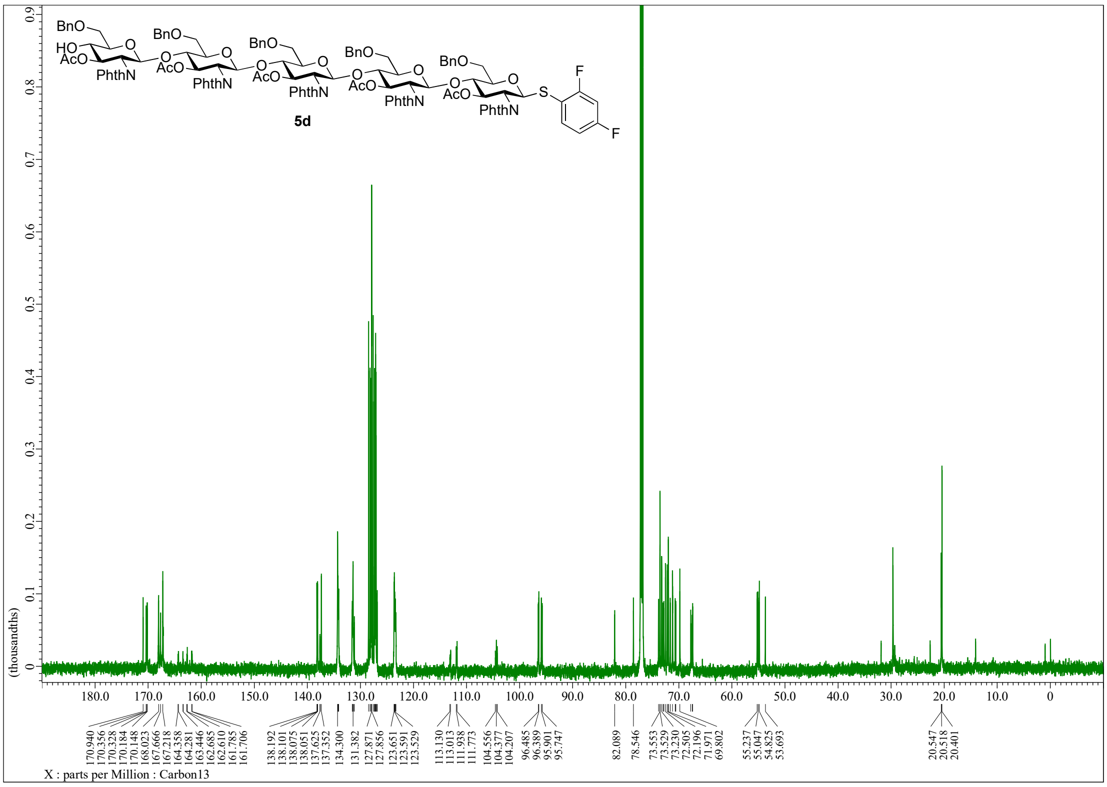

H,H-COSY

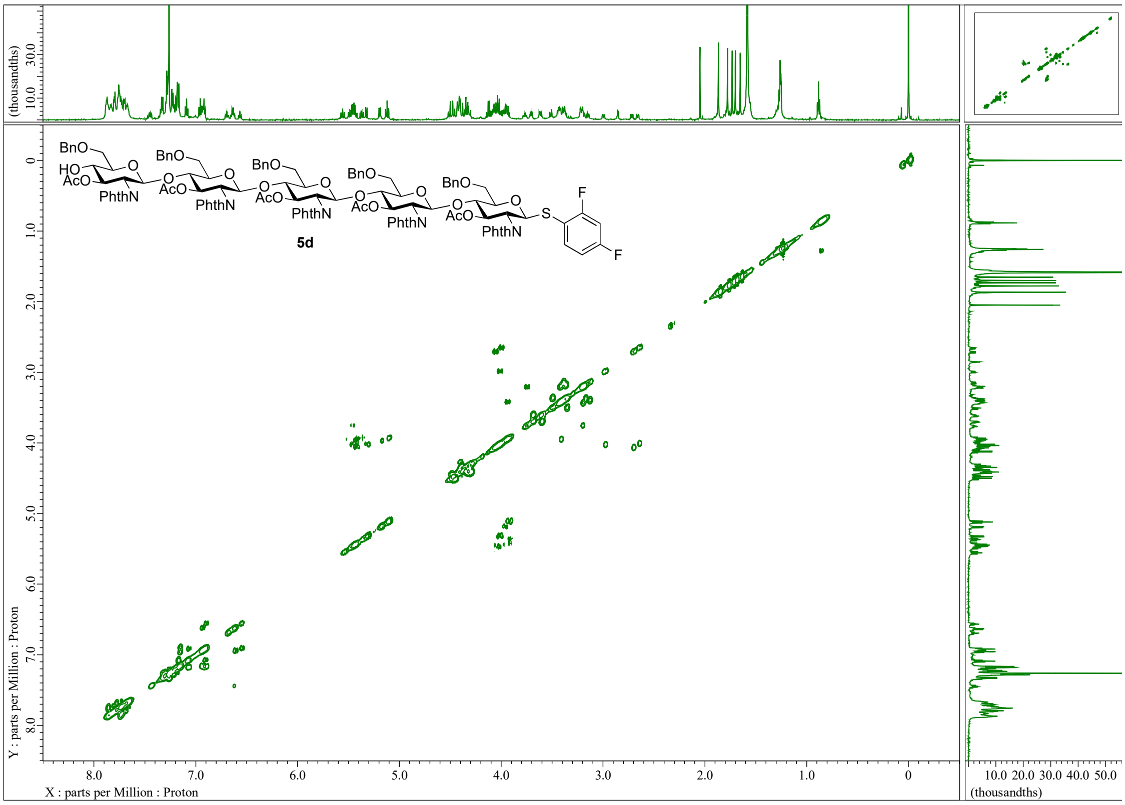

BnO
HO
AcO
PhthN
O
S
F
F
5d
(thousandths)
Y : parts per Million : Proton
X : parts per Million : Proton

HMQC

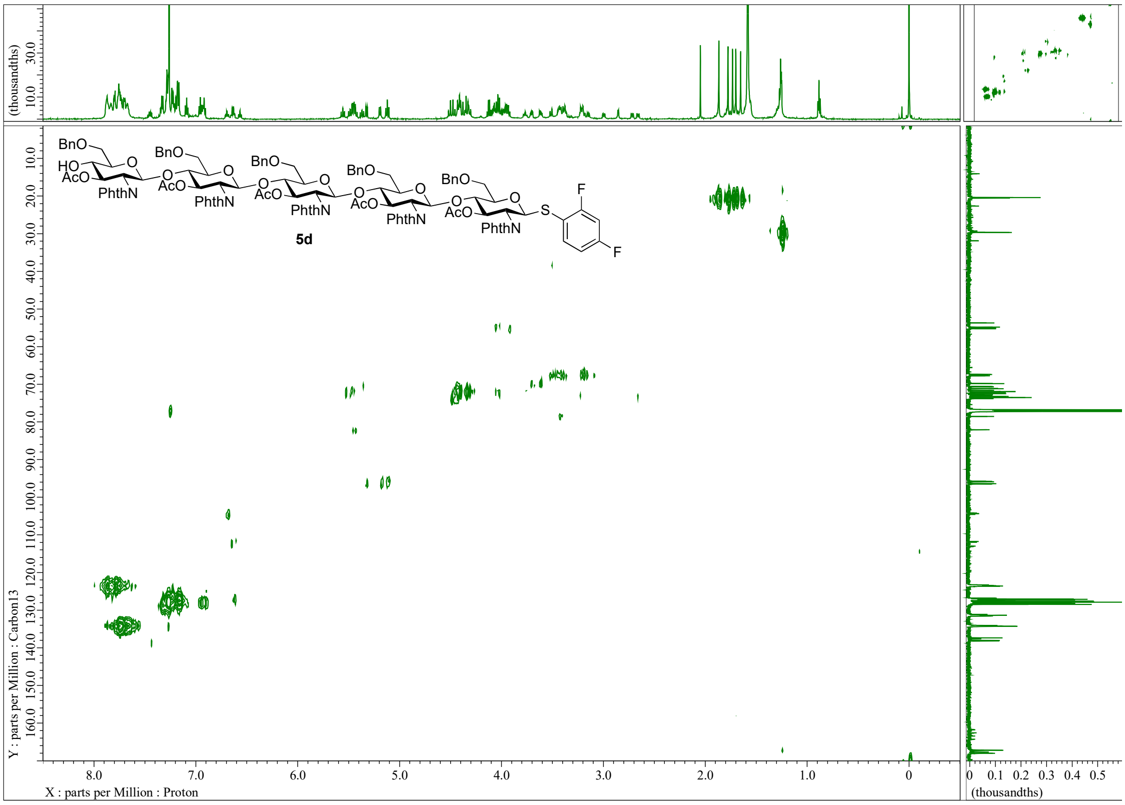

BnO
HO
AcO
PhthN
O
S
F
F
5d
(thousandths)
Y : parts per Million : Carbon13
X : parts per Million : Proton
